# Organocatalytic asymmetric nitroso aldol reaction of α*-*substituted malonamates

**DOI:** 10.3762/bjoc.18.25

**Published:** 2022-02-21

**Authors:** Ekta Gupta, Narendra Kumar Vaishanv, Sandeep Kumar, Raja Krishnan Purshottam, Ruchir Kant, Kishor Mohanan

**Affiliations:** 1 Medicinal & Process Chemistry Division, CSIR-Central Drug Research Institute, BS-10/1, Sector 10, Jankipuram extension, Sitapur Road, P.O. Box 173, Lucknow 226031, India; 2 Sophisticated Analytical Instrument Facility CSIR-Central Drug Research Institute, BS-10/1, Sector 10, Jankipuram extension, Sitapur Road, P.O. Box 173, Lucknow 226031, India; 3 Molecular and Structural Biology Division, CSIR-Central Drug Research Institute, BS-10/1, Sector 10, Jankipuram extension, Sitapur Road, P.O. Box 173, Lucknow 226031, India; 4 Academy of Scientific and Innovative Research, Ghaziabad, 201002, India

**Keywords:** enantioselective, malonamate, nitroso aldol reaction, N-selectivity, Takemoto catalyst

## Abstract

A practical enantioselective N-selective nitroso aldol reaction of α-methylmalonamates with a nitrosoarene is reported. The reaction employs the Takemoto thiourea catalyst for the induction of enantioselectivity, and the corresponding optically active oxyaminated malonamates were obtained in reasonably good yields.

## Introduction

Nitrosoarenes are versatile building blocks frequently encountered in organic synthesis as precursors for the synthesis of nitrogen and oxygen-containing molecules [1–5]. The high reactivity caused by the polarization of the N–O bond enables the nitrosoarenes to undergo a wide range of transformations in a chemo- and regioselective manner [6–8]. The noteworthy and widely explored transformations of nitrosoarenes include nitroso ene reactions [9–11], Diels–Alder cycloadditions [12–18], and nitroso aldol reactions [19–23]. Among the various applications of nitrosoarenes, the asymmetric nitroso aldol reaction to achieve optically active α-aminoxy and α-hydroxyamino carbonyl compounds has received considerable attention in the past decades [24]. In 2003, the Yamamoto group demonstrated for the first time that nitrosobenzene could be used as a practical reagent for the catalytic enantioselective α-aminoxylation using a silver-BINAP catalyst combination [25]. Later, the same group could successfully tune the catalytic system to control the regioselectivity in the addition of metal enolate to nitrosoarenes to achieve an α-hydroxyamination [26]. Since then, several groups have shown the use of metal-catalyzed nitroso aldol reactions as a practical tool for the selective introduction of amino or hydroxy moieties at the α-position of a carbonyl function [27–30].

**Scheme 1:**
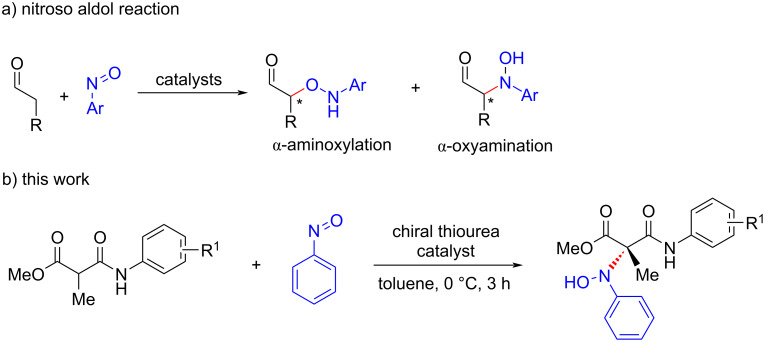
Catalytic asymmetric nitroso aldol reaction.

The last two decades have witnessed an upsurge of interest in the development of organocatalyzed nitroso aldol reactions in addition to the metal-catalyzed reactions [31–36]. The most successful among them are the ʟ-proline-catalyzed reactions of enolizable aldehydes with nitrosoarenes [37–43]. Besides ʟ-proline and its derivatives, various secondary amines derived from BINOL and cinchona alkaloids were also found useful in catalyzing the nitroso aldol reaction [44–48]. Surprisingly, the utility of thiourea catalysts in nitroso aldol reactions remains far less developed. The scattered reports where bifunctional thiourea catalysis was found useful for this type of reaction, describe the hydroxyamination of oxindoles and β-ketoamides [49–54]. Recently, it has been shown that malonate derivatives such as malonate half thioesters and malonamides could be effectively used in various enantioselecive addition reactions [55–60]. In this context, Chen and co-workers reported a squaramide-catalyzed asymmetric nitroso aldol reaction of cyclic β-ketoesters and malonamate [61]. Inspired by this, we decided to investigate the use of malonamate in the asymmetric nitroso aldol reaction using thiourea catalysis. Herein, we report a novel nitroso aldol reaction of malonamates with nitrosoarene which provides facile access to chiral hydroxyamino malonamates having a quaternary carbon stereocenter (Scheme 1).

## Results and Discussion

Initially, we chose the Takemoto catalyst to promote the model reaction between methyl *N*-bromophenyl-α-methylmalonamate (**1a**) and nitrosobenzene (**2a**) in toluene at 25 °C. Pleasingly, the hydroxyamination reaction proceeded smoothly to give the nitroso aldol product **4a** in 80% yield and 60% ee (Table 1, entry 1). The influence of temperature was evaluated subsequently, and the reaction conducted at 0 °C without altering other parameters gave a better result furnishing the product in 90% yield and 90% enantiomeric excess (Table 1, entry 2). Further lowering of the reaction temperature did not improve the enantioselectivity and slowed down the reaction (Table 1, entries 3 and 4). Our next attempts on the improvement of enantioselectivity focused on the screening of various bifunctional H-bonding catalysts, and in this regard, the reaction catalyzed by quinine-derived thiourea catalyst **3b** furnished the product **4a** in 55% yield and 71% ee (Table 1, entry 5). The other enantiomer was obtained when the reaction was carried out using the squaramide catalyst **3c**, however, with low enantioselectivity (Table 1, entry 6). Disappointingly, the reaction catalyzed by ʟ-proline-derived catalysts gave very low enantioselectivity (Table 1, entries 7 and 8). Having identified Takemoto’s catalyst as the most efficient one for this transformation, our attempts to enhance the enantioselectivity centered on the variation of solvents. The reaction was screened using various polar and nonpolar solvents, and toluene was found suitable in terms of the reaction rate, yield, and enantioselectivity (Table 1, entries 9–13). Other solvents such as EtOAc, DCM, chloroform, and hexane provided **4a** with moderate enantioselectivities, and the reaction failed when DMF was used as the solvent. When the catalyst loading was reduced to 10 and 5 mol %, the enantioselectivity remained reasonably good, but the reaction yield was substantially affected.

**Table 1:** Optimization of the reaction conditions.^a^

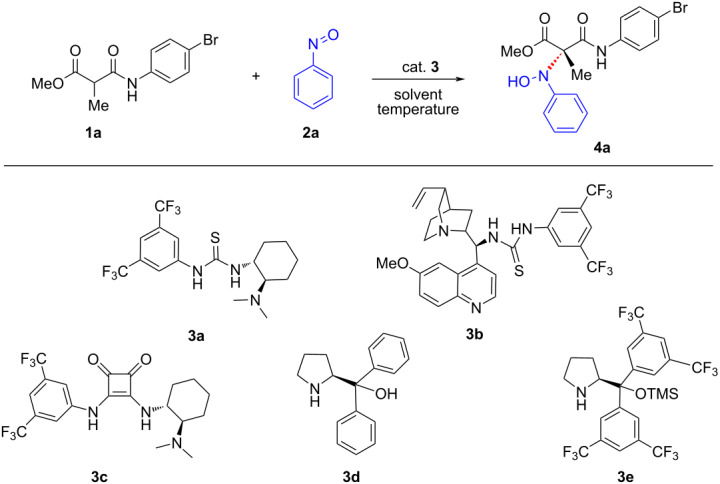						

entry	cat. **3**	solvent	temperature (°C)	time (h)	yield (%)^b^	ee (%)^c^

1	**3a**	toluene	25	3	80	60
2	**3a**	toluene	0	3	90	90
3	**3a**	toluene	−10	12	80	86
4	**3a**	toluene	−20	24	70	78
5	**3b**	toluene	0	4	55	71
6	**3c**	toluene	0	12	58	−41
7	**3d**	toluene	0	24	42	15
8	**3e**	toluene	0	24	35	13
9	**3a**	DCM	0	3	90	77
10	**3a**	EtOAc	0	3	72	78
11	**3a**	CHCl_3_	0	3	72	66
12	**3a**	hexane	0	3	54	73
13	**3a**	DMF	0	–	–	–
14^d^	**3a**	toluene	0	5	71	84
15^e^	**3a**	toluene	0	8	70	86

^a^General conditions: **1a** (0.20 mmol), **2a** (0.24 mmol), **3** (0.04 mmol), solvent (3.0 mL). ^b^Isolated yield after silica gel column chromatography. ^c^Determined by chiral HPLC analysis. ^d^The reaction conducted using 10 mol % of the catalyst. ^e^The reaction conducted using 5 mol % of the catalyst.

**Scheme 2:**
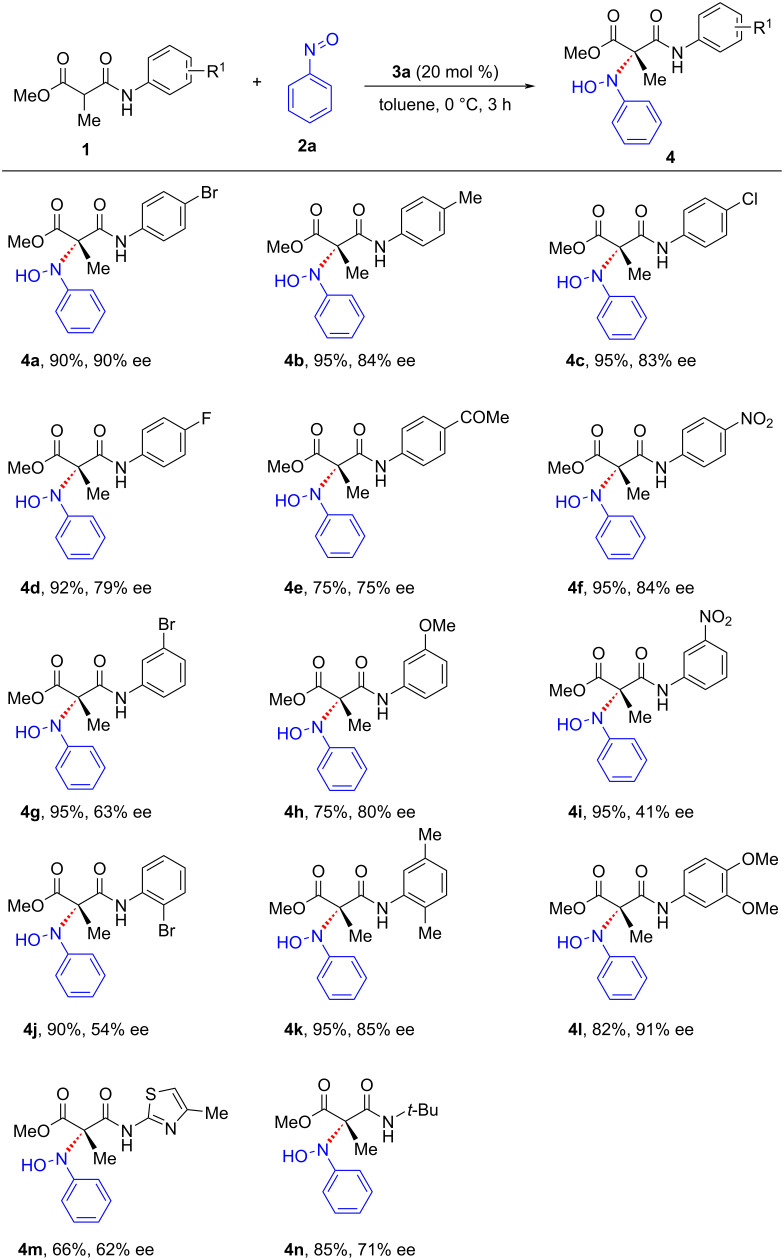
Variation of the amide moiety of malonamates. General conditions: **1** (0.20 mmol), **2a** (0.24 mmol), **3a** (0.04 mmol), toluene (3.0 mL). Yields refer to isolated yields after silica gel column chromatography. Enantioselectivities were determined by chiral HPLC analysis.

**Figure 1:**
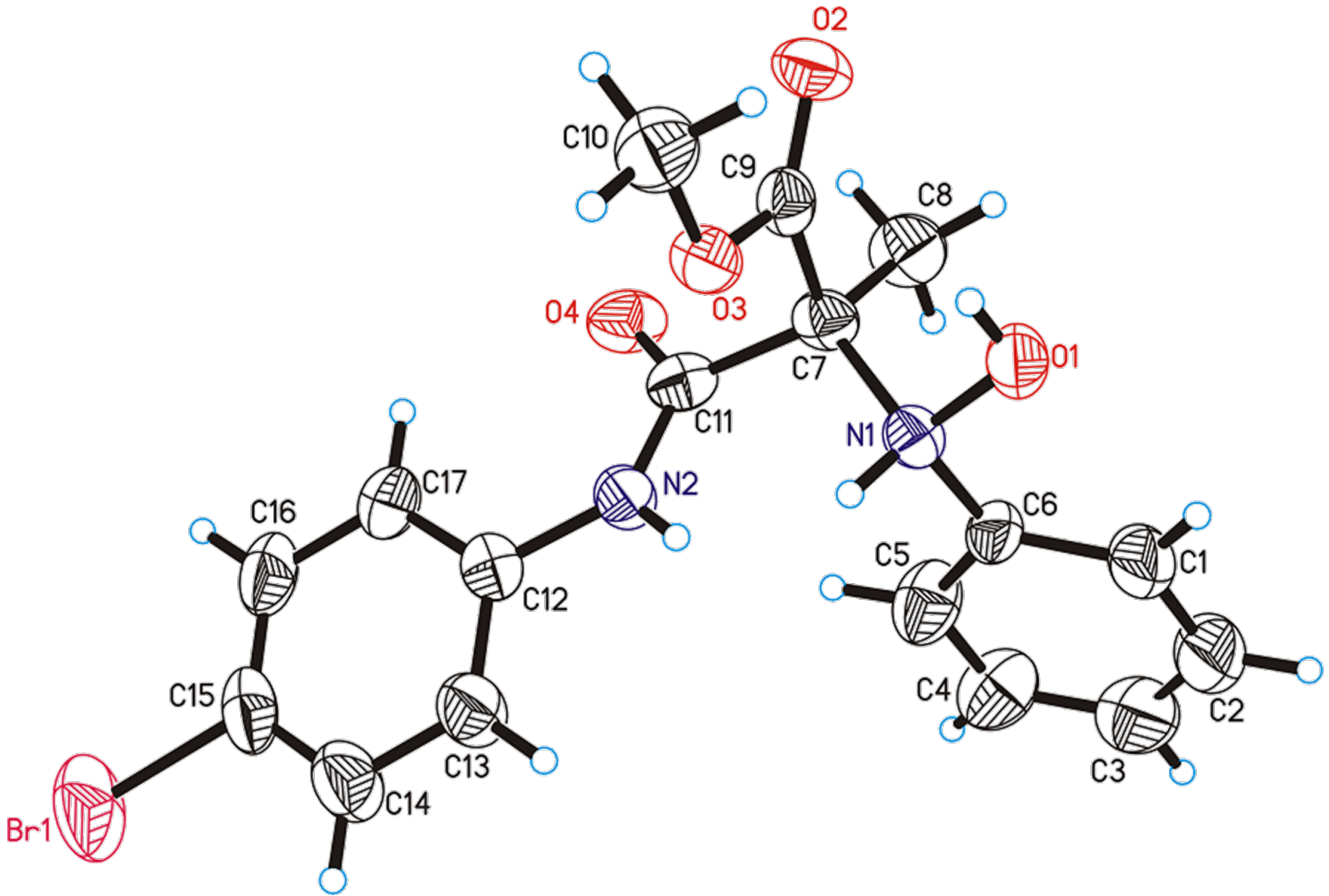
ORTEP diagram drawn with 30% ellipsoid probability for non-H atoms of the crystal structure of chiral compound **4a** determined at 293 K. The absolute configuration of C7 is *S*.

Having identified the optimal reaction conditions, we proceeded to evaluate the generality of this nitroso aldol reaction with respect to the amide component of malonamate (Scheme 2). Pleasingly, our strategy was found to be operational with malonamates bearing electronically different substituents such as halo, nitro, acetyl, and alkyl at the *para*-position of the phenyl ring and the corresponding oxyaminated products were obtained in excellent yields and good enantioselectivities (**4a**–**f**). The single crystal X-ray analysis of the product **4a** established the absolute stereochemistry which was found to be *S* (Figure 1) [62]. There was a significant drop in the enantioselectivity when malonamates bearing substitutions at *meta-* and *ortho-*positions were used, except for the reaction carried out using methoxy substitution at the 3-position where the corresponding oxyaminated product was obtained in 75% yield and 80% ee (**4g**–**j**). The disubstituted malonamates underwent facile oxyamination, and the products were obtained in excellent yields and enantioselectivities (**4k**, **4l**). Of note, the reaction was also feasible with heterocyclic malonamate, albeit with moderate yield and enantioselectivity (**4m**). Aliphatic malonamate was also found viable for this transformation, giving the oxyaminated product in good yield and moderate enantioselectivity (**4n**).

**Scheme 3:**
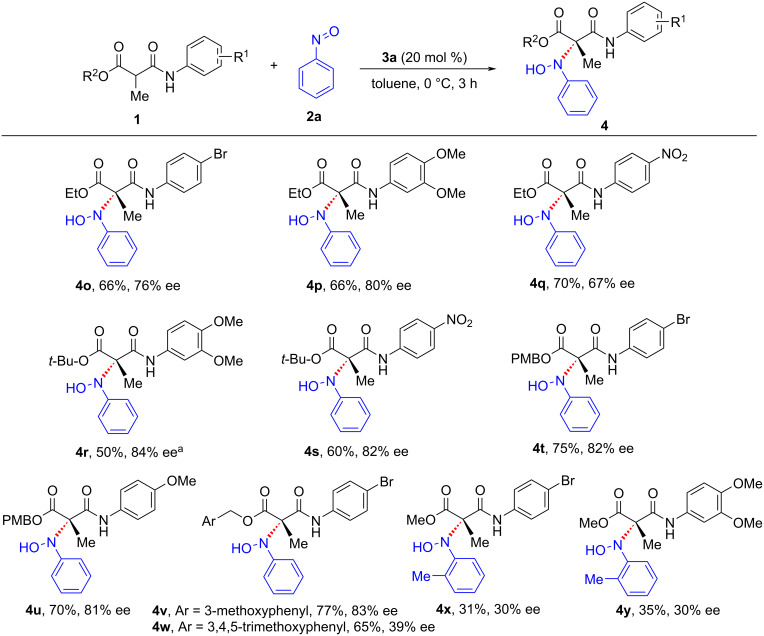
Variation of ester moiety of malonamates and nitrosoarenes. General conditions: **1** (0.20 mmol), **2a** (0.24 mmol), **3a** (0.04 mmol), toluene (3.0 mL). Yields refer to isolated yields after silica gel column chromatography. Enantioselectivities were determined by chiral HPLC analysis. ^a^Reaction run for 6 h.

Subsequently, the scope of the transformation was investigated with various alkyl esters of malonamate (Scheme 3). In addition to the methyl ester, the reaction was found to proceed smoothly with ethyl, *tert*-butyl, and *p*-methoxybenzyl esters of malonamate to furnish the oxyaminated products in good yields and moderate to good enantioselectivities (**4o**–**u**). Pleasingly, the reactions carried out using *m*-methoxybenzyl and 3,4,5-trimethoxybenzyl esters of malonamate afforded the corresponding products in good yields and enantioselectivity (**4v**, **4w**). The scope was further expanded by carrying out a reaction using *o*-methylnitrosobenzene, and in this case, the reaction proceeded smoothly to afford the corresponding oxyaminated product, albeit with low enantioselectivity (**4x**, **4y**). Disappointingly, the reactions carried out by varying the α-substitution did not afford the desired product.

**Scheme 4:**
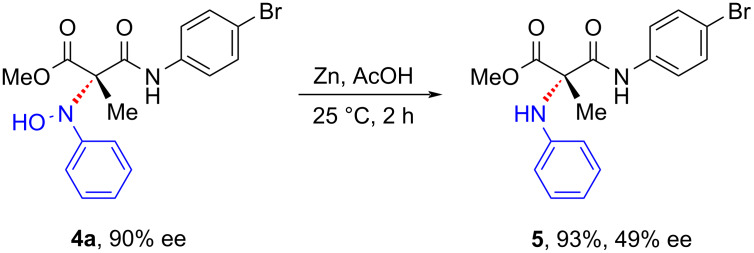
Synthetic transformation.

In order to demonstrate the synthetic utility of the oxyaminated compounds, the reductive cleavage of the N–O bond was attempted under Zn/AcOH conditions. Pleasingly, the reaction afforded the aniline derivative in good yield, albeit with a considerable diminishment in the ee (Scheme 4).

**Figure 2:**
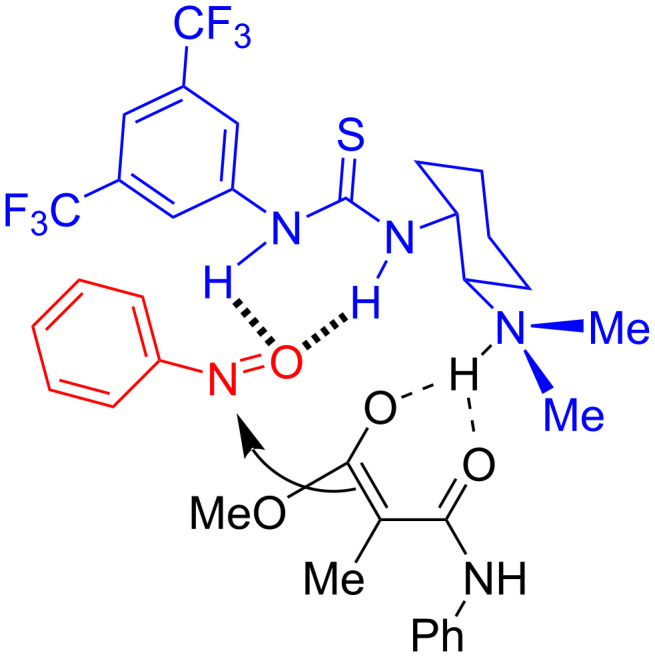
Proposed transition state for the nitroso aldol reaction.

Based on the literature reports and the stereochemical outcome, a plausible transition state is proposed, as shown in Figure 2. The activation of nitrosobenzene was achieved by the intramolecular hydrogen-bonding of the thiourea moiety with the oxygen of the nitrosobenzene. The tertiary amine, present in the catalyst acts as a base in assisting the deprotonation of the highly acidic malonamate to generate the corresponding enolate. Subsequently, a face-selective nucleophilic addition of the enolate to nitroso selective takes place to afford the nitroso aldol product.

## Conclusion

In summary, an efficient organocatalytic asymmetric nitroso aldol reaction of α-methylmalonamate has been reported. The reaction utilizes the well-known Takemoto catalyst, and this protocol demonstrates for the first time the use of malonamate as a pro-nucleophile in an enantioselective addition reaction. The mild reaction conditions allow the use of various functionalized malonamates. Given the importance of highly functionalized α-amino acid derivatives, the present strategy could be useful in generating a wide range of α-oxyamino malonamates which may serve as a potential platform for the synthesis of medicinally relevant structural units.

## Experimental

**General experimental procedure for the thiourea-catalyzed nitroso aldol reaction of malonamates:** To an oven-dried round-bottomed flask equipped with a magnetic stirring bar were added α-methylmalonamate **1a** (57 mg, 0.20 mmol, 1 equiv), nitrosobenzene **2a** (26 mg, 0.24 mmol, 1.2 equiv) and (*R*,*R*)-TUC **3a** (17 mg, 0.04 mmol, 0.2 equiv). Then, the round-bottomed flask was sealed, evacuated, and backfilled with nitrogen. The mixture was dissolved in 3 mL of anhydrous toluene and was kept stirring at 0 °C for the specified time. After the completion of the reaction, as indicated by TLC, the solvent was evaporated and the residue extracted using ethyl acetate and water. The organic layer was dried over Na_2_SO_4_ and evaporated under reduced pressure. The residue was purified using column chromatography (100–200 mesh silica gel) using EtOAc/hexane as the eluent to afford product **4a** as white solid (71 mg, 90%). *R*_f_ 0.20 (EtOAc/hexane 3:7); mp 115–117 °C; ^1^H NMR (400 MHz, CDCl_3_) δ 9.04 (s, 1H), 7.47–7.44 (m, 5H), 7.31–7.26 (m, 2H), 7.20–7.14 (m, 3H), 3.81 (s, 3H), 1.59 (s, 3H) ppm; ^13^C NMR (100 MHz, CDCl_3_) δ 171.5 (C), 167.0 (C), 147.1 (C), 136.5 (C), 132.1 (CH), 132.1(CH), 128.9 (CH), 128.9 (CH), 126.1 (CH), 122.2 (CH), 122.2 (CH), 121.6 (CH), 121.6 (CH), 117.4 (C), 76.8 (C), 53.5 (CH_3_), 17.9 (CH_3_) ppm. The enantiomeric excess was determined by HPLC on a Chiralpak IC column (hexane/ethanol 90:10 v/v, flow rate 1.0 mL/min, 254 nm, τ_minor_ = 13.0 min, τ_major_ = 13.9 min, 90% ee). HRMS (*m*/*z*): [M + Na]^+^ calcd for C_17_H_17_BrN_2_NaO_4_^+^, 415.0264; found, 415.0278.

## Supporting Information

File 1 Detailed experimental procedures, complete characterization data for all compounds, single-crystal X-ray data of **4a**, copies of NMR spectra, and HPLC chromatograms.
