# Supplementary material for: Organocatalytic asymmetric nitroso aldol reaction of α-substituted malonamates
Source: Beilstein J Org Chem. 2022 Feb 21;18:217–24. doi: 10.3762/bjoc.18.25 (PMC8895028; doi:10.3762/bjoc.18.25)
Supplement: File 1 — Detailed experimental procedures, complete characterization data for all compounds, single-crystal X-ray data of 4a, copies of NMR spectra, and HPLC chromatograms. [file Beilstein_J_Org_Chem-18-217-s001.pdf]

BEILSTEIN JOURNAL OF ORGANIC CHEMISTRY

Supporting Information

for

Organocatalytic asymmetric nitroso aldol reaction of α-substituted malonamates

Ekta Gupta, Narendra Kumar Vaishanv, Sandeep Kumar, Raja Krishnan Purshottam, Ruchir Kant and Kishor Mohanan

Beilstein J. Org. Chem. **2022,** *18,* 217–224. doi:10.3762/bjoc.18.25

Detailed experimental procedures, complete characterization data for all compounds, single-crystal X-ray data of 4a, copies of NMR spectra, and HPLC chromatograms

License and Terms: This is a supporting information file under the terms of the Creative Commons Attribution License (https://creativecommons.org/licenses/by/4.0). Please note that the reuse, redistribution and reproduction in particular requires that the author(s) and source are credited and that individual graphics may be subject to special legal provisions.
The license is subject to the *Beilstein Journal of Organic Chemistry* terms and conditions: (https://www.beilstein-journals.org/bjoc/terms)

Table of contents

1. General experimental information S2
2. Characterization data for compounds **4a**–**y** and **5** S3
3. X-ray data of compound **4a** S16
4. References S17
5. Copies of ^{1}H and ^{13}C NMR spectra for all compounds S18
6. HPLC Chromatograms of all compounds S44

General experimental information:

Unless otherwise specified, all reactions were carried out under air atmosphere in oven-dried round-bottomed flasks. The reactions were monitored by TLC visualized by UV (254 nm) and/or with iodine. Flash chromatography was performed on 100–200 mesh silica gel using the gradient system ethyl acetate/hexane. NMR data were recorded with a Bruker AV 400 MHz spectrometer in $CDCl_3$ using as internal standards the residual $CHCl_3$ signal for ^{1}H NMR (δ = 7.26 ppm) and the deuterated solvent signal for ^{13}C NMR (δ = 77.16 ppm). Coupling constants are given in hertz (Hz) and the standard abbreviations are used to describe the signal multiplicities. Melting points were measured with a Büchi B-540 melting point apparatus and are uncorrected. High resolution mass spectra were obtained using a Q-TOF mass spectrometer. The ee values were determined on a Waters Standard HPLC System using chiral column with hexane and ethanol as eluent, wavelength = 254 nm. All commercially available reagents were used as received. α-Methylmalonamates **1a–u** were synthesized following a literature procedure.[1]

Characterization data for compounds 4a–y and 5

(*S*)-Methyl 3-((4-bromophenyl)amino)-2-(hydroxy(phenyl)amino)-2-methyl-3-oxopropanoate (4a)

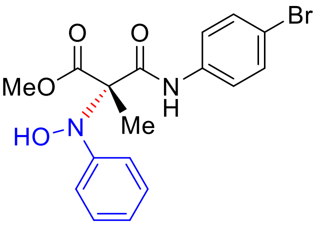

$C_{17}H_{17}BrN_2O_4$
Mw: 392.03

Following the general procedure, treatment of methyl 3-((4-bromophenyl)amino)-2-methyl-3-oxopropanoate (**1a**, 57 mg, 0.20 mmol) with nitrosobenzene (**2a**, 26 mg, 0.24 mmol) in the presence of (*R*,*R*)-TUC (**3a**, 17 mg, 0.04 mmol) in toluene (3 mL) at 0 °C for 3 h followed by column chromatography afforded the product **4a** as white solid (71 mg, 90%). **R*f*** (EtOAc/hexane 3:7) = 0.20. **Mp** 115-117 °C. **^{13}C NMR** (100 MHz, δ ppm/$CDCl_3$): 171.5 (C), 167.0 (C), 147.1 (C), 136.5 (C), 132.1 (CH), 132.1(CH), 128.9 (CH), 128.9 (CH), 126.1 (CH), 122.2 (CH), 122.2 (CH), 121.6 (CH), 121.6 (CH), 117.4 (C), 76.8 (C), 53.5 (CH_3), 17.9 (CH_3). **^{1}H NMR** (400 MHz, δ ppm/$CDCl_3$): 9.04 (s, 1H), 7.47-7.44 (m, 5H), 7.31-7.26 (m, 2H), 7.20-7.14 (m, 3H), 3.81 (s, 3H), 1.59 (s, 3H). Enantiomeric excess was determined by HPLC on Chiralpak IC column (Hexane/Ethanol 90:10 *V*/*V*, flow rate 1.0 mL/min, 254 nm, τ_{minor} = 13.0 min, τ_{major} = 13.9 min, 90% ee). **HRMS** for $C_{17}H_{17}BrN_2NaO_4^+$: calcd. $[M+Na]^+$: 415.0264, found: 415.0278, $[M+2+Na]^+$: 417.0257.

(*S*)-Methyl 2-(hydroxy(phenyl)amino)-2-methyl-3-(*p*-tolylamino)propanoate (4b)

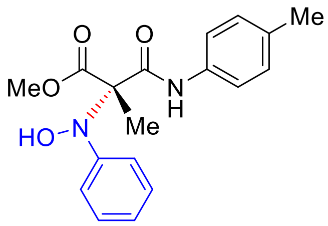

$C_{18}H_{20}N_2O_4$
Mw: 328.14

Following the general procedure, treatment of methyl 2-methyl-3-oxo-3-(*p*-tolylamino)propanoate (**1b**, 44 mg, 0.20 mmol) with nitrosobenzene (**2a**, 26 mg, 0.24 mmol) in the presence of (*R*,*R*)-TUC (**3a**, 17 mg, 0.04 mmol) in toluene (3 mL) at 0 °C for 3 h followed by column chromatography afforded the product **4b** as white solid (62 mg, 95%). **R*f*** (EtOAc/hexane 3:7) = 0.28. **Mp** 149-151 °C. **^{13}C NMR** (100 MHz, δ ppm/$CDCl_3$): 171.7 (C), 166.5 (C), 147.3 (C), 134.9 (C), 134.4 (C), 129.6 (CH), 129.6 (CH), 128.8 (CH), 128.8 (CH), 125.7 (CH), 121.9 (CH), 121.9 (CH), 120.0 (CH), 120.0 (CH), 77.2 (C), 53.3 (CH_3), 20.9 (CH_3), 17.6 (CH_3). **^{1}H NMR** (400 MHz, δ ppm/$CDCl_3$): 9.00 (s, 1H), 7.48 (d, *J* = 8.4 Hz, 2H), 7.45 (s, 1H), 7.34-7.26 (m, 4H), 7.17 (d, *J*= 8.0, 3H), 3.85 (s, 3H), 2.36 (s, 3H), 1.65 (s, 3H). Enantiomeric excess was determined by HPLC on Chiralpak IC column (Hexane/Ethanol 90:10 *V*/*V*, flow rate 1.0 mL/min, 254 nm, τ_{minor} = 28.9 min, τ_{major} = 32.8 min, 84% ee). **HRMS** for $C_{18}H_{20}N_2NaO_4^+$: calcd. $[M+Na]^+$: 351.1315, found: 351.1323.

(*S*)-Methyl 3-((4-chlorophenyl)amino)-2-(hydroxy(phenyl)amino)-2-methyl-3-oxopropanoate (4c)

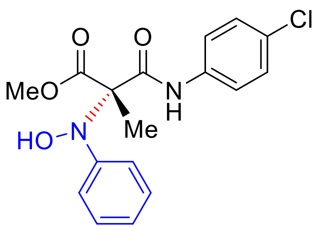

$C_{17}H_{17}ClN_2O_4$
Mw: 348.09

Following the general procedure, treatment of methyl 3-((4-chlorophenyl)amino)-2-methyl-3-oxopropanoate (**1c**, 48 mg, 0.20 mmol) with nitrosobenzene (**2a**, 26 mg, 0.24 mmol) in the presence of (*R*,*R*)-TUC (**3a**, 17 mg, 0.04 mmol) in toluene (3 mL) at 0 °C for 3 h followed by column chromatography afforded the product **4c** as white solid (66 mg, 95%). **R*f*** (EtOAc/hexane 3:7) = 0.5. **Mp** 134-136 °C. **^{13}C NMR** (100 MHz, δ ppm/$CDCl_3$): 171.6 (C), 167.0 (C), 147.1 (C), 136.0 (C), 129.8 (C), 129.2 (CH), 129.2(CH), 129.0 (CH), 129.0 (CH), 126.1 (CH), 122.2 (CH), 122.2 (CH), 121.3 (CH), 121.3 (CH), 76.8 (C), 53.5 (CH_3), 18.1 (CH_3).**^{1}H NMR** (400 MHz, δ ppm/$CDCl_3$): 9.03 (s, 1H), 7.51 (d, *J* = 8.8 Hz, 2H), 7.39 (s, 1H), 7.31-7.28 (m, 4H), 7.21-7.15 (m, 3H), 3.82 (s, 3H), 1.60 (s, 3H). Enantiomeric excess was determined by HPLC on Chiralpak IC column (Hexane/Ethanol 90:10 *V*/*V*, flow rate 1.0 mL/min, 254 nm, τ_{minor} = 6.7 min, τ_{major} = 7.2 min, 83% ee). **HRMS** for $C_{17}H_{18}ClN_2O_4^+$: calcd. $[M+H]^+$: 349.0950, found: 349.0940, $[M+2+H]^+$: 351.0910.

(*S*)-Methyl 3-((4-fluorophenyl)amino)-2-(hydroxy(phenyl)aminomethyl-3-oxopropanoate (4d)

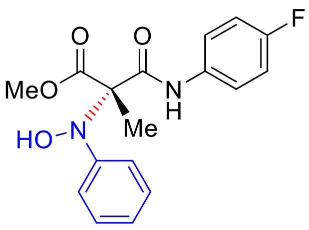

$C_{17}H_{17}FN_2O_4$
Mw: 332.12

Following the general procedure, treatment of methyl 3-((4-fluorophenyl)amino)-2-methyl-3-oxopropanoate (**1d**, 45 mg, 0.20 mmol) with nitrosobenzene (**2a**, 26 mg, 0.24 mmol) in the presence of (*R*,*R*)-TUC (**3a**, 17 mg, 0.04 mmol) in toluene (3 mL) at 0 °C for 3 h followed by column chromatography afforded the product **4d** as white solid (61 mg, 92%). **R*f*** (EtOAc/hexane 3:7) = 0.45. **Mp** 116-118°C. **^{13}C NMR** (100 MHz, δ ppm/$CDCl_3$): 171.7 (C), 167.0 (C), 159.7 (d, *J* = 242.7 Hz, C), 147.1 (C), 133.5 (C), 128.9 (CH), 128.9 (CH), 126.0 (CH), 122.1 (CH), 122.1 (CH), 121.9 (d, *J* = 7.8 Hz, CH), 121.9 (d, *J* = 7.8 Hz, CH), 115.8 (d, *J* = 22.4 Hz, CH), 115.8 (d, *J* = 22.4 Hz, CH), 76.7 (C), 53.4 (CH_3), 18.0 (CH_3).**^{1}HNMR** (400 MHz, δ ppm/$CDCl_3$): 8.98 (s, 1H), 7.55-7.51 (m, 2H), 7.33-7.20 (m, 5H), 7.17 (t, *J* = 7.2 Hz, 1H), 7.04 (t, *J* = 8.8 Hz, 2H), 3.84 (s, 3H), 1.61 (s, 3H). Enantiomeric excess was determined by HPLC on Chiralpak IC column (Hexane/Ethanol 90:10 *V*/*V*, flow rate 1.0 mL/min, 254 nm, τ_{minor} = 27.4 min, τ_{major} = 30.9 min, 79% ee). **HRMS** for $C_{17}H_{17}FN_2NaO_4^+$: calcd. $[M+Na]^+$: 355.1065, found: 355.1063.

(*S*)-Methyl 3-((4-acetylphenyl)amino)-2-(hydroxy(phenyl)amino)-2-methyl-3-oxopropan-oate (4e)

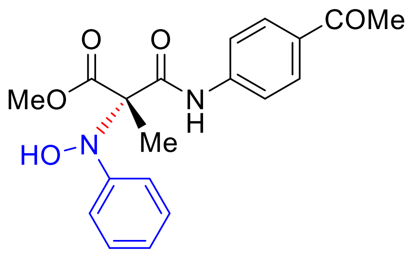

$C_{19}H_{20}N_2O_5$
Mw: 356.14

Following the general procedure, treatment of methyl 3-((4-acetylphenyl)amino)-2-methyl-3-oxopropanoate (**1e**, 50 mg, 0.20 mmol) with nitrosobenzene (**2a**, 26 mg, 0.24 mmol) in the presence of (*R*,*R*)-TUC (**3a**, 17 mg, 0.04 mmol) in toluene (3 mL) at 0 ˚C for 3 h followed by column chromatography afforded the product **4e** as white white solid (53 mg, 75%). **R*f*** (EtOAc/hexane 3:7) = 0.39. **Mp** 130-132 ˚C. **^{13}C NMR** (100 MHz, δ ppm/CDCl$_3$): 197.4 (C), 171.1 (C), 167.4 (C), 147.1 (C), 141.9 (C), 133.2 (C), 129.9 (CH), 129.9 (CH), 128.9 (CH), 128.9 (CH), 126.2 (CH), 122.4 (CH), 122.4 (CH), 119.3 (CH), 119.3 (CH), 77.1 (C), 53.4 (CH$_3$), 26.5 (CH$_3$), 17.7 (CH$_3$).**^{1}H NMR** (400 MHz, δ ppm/CDCl$_3$): 9.27 (s, 1H), 7.88 (d, *J* = 8.0 Hz, 2H), 7.62 (d, *J* = 8.4 Hz, 3H), 7.28-7.18 (m, 4H), 7.13 (t, *J* = 7.0 Hz, 1H), 3.77 (s, 3H), 2.52 (s, 3H), 1.57 (s, 3H). Enantiomeric excess was determined by HPLC on Chiralpak IC column (Hexane/Ethanol 90:10 *V*/*V*, flow rate 1.0 mL/min, 254 nm, τ_{minor} = 19.0 min, τ_{major} = 21.1 min, 75% ee). **HRMS** for $C_{19}H_{20}N_2NaO_5^+$: calcd. [M+Na]$^+$: 379.1264, found: 379.1288.

(*S*)-Methyl 2-(hydroxy(phenyl)amino)-2-methyl-3-((4-nitrophenyl)amino)-3-oxopropan-oate (4f)

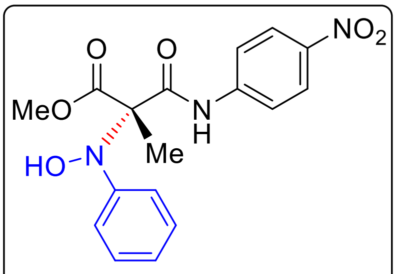

$C_{17}H_{17}N_3O_6$
Mw: 359.11

Following the general procedure, treatment of methyl 2-methyl-3-((4-nitrophenyl)amino)-3-oxopropanoate (**1f**, 51 mg, 0.20 mmol) with nitrosobenzene (**2a**, 26 mg, 0.24 mmol) in the presence of (*R*,*R*)-TUC (**3a**, 17 mg, 0.04 mmol) in toluene (3 mL) at 0 °C for 3 h followed by column chromatography afforded the product **4f** as a yellow solid (68 mg, 95%). **R*f*** (EtOAc/hexane 3:7) = 0.33. **Mp** 161-163 °C. **^{13}C NMR** (100 MHz, δ ppm/CDCl$_3$): 170.8 (C), 167.7 (C), 146.7 (C), 144.0 (C), 143.3 (C), 129.1 (CH), 129.1 (CH), 126.6 (CH), 125.3 (CH), 125.3 (CH), 122.5 (CH), 122.5 (CH), 119.6 (CH), 119.6 (CH), 76.8 (C), 53.6 (CH$_3$), 18.4 (CH$_3$).**^{1}H NMR** (400 MHz, δ ppm CDCl$_3$): 9.39 (s, 1H), 8.23 (d, *J* = 9.2 Hz, 2H), 7.77 (d, *J* = 8.8 Hz, 2H), 7.35-7.26 (m, 3H), 7.22 (d, *J* = 8.4 Hz, 3H), 3.85 (s, 3H), 1.62 (s, 3H). Enantiomeric excess was determined by HPLC on Chiralpak IC column (Hexane/Ethanol 90:10 *V*/*V*, flow rate 1.0 mL/min, 254 nm, τ_{minor} = 10.3 min, τ_{major} = 11.5 min, 85% ee). **HRMS** for$C_{17}H_{17}N_3NaO_6^+$: calcd. [M+Na]$^+$: 382.1010, found: 382.1002.

(*S*)-Methyl 3-((3-bromophenyl)amino)-2-(hydroxy(phenyl)amino)-2-methyl-3-oxopropanoate (4g)

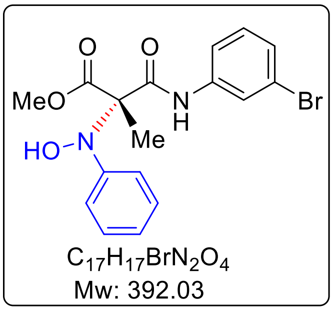

Following the general procedure, treatment of methyl 3-((3-bromophenyl)amino)-2-methyl-3-oxopropanoate (**1g**, 57 mg, 0.20 mmol) with nitrosobenzene (**2a**, 26 mg, 0.24 mmol) in the presence of (*R*,*R*)-TUC (**3a**, 17 mg, 0.04 mmol) in toluene (3 mL) at 0 °C for 3 h followed by column chromatography afforded the product **4g** as white solid (74 mg, 95%). **R*f*** (EtOAc/hexane 3:7) = 0.26. **Mp** 145-147 °C.**^{13}C NMR** (100 MHz, δ ppm/$CDCl_3$): 171.5 (C), 167.1 (C), 147.0 (C), 138.7 (C), 130.5 (CH), 129.0 (CH), 129.0 (CH), 127.8 (CH), 126.1 (CH), 123.0 (CH), 122.8 (C), 122.2 (CH), 122.2 (CH), 118.5 (CH), 76.8 (C), 53.5 (CH_3), 18.0 (CH_3). **^{1}H NMR** (400 MHz, δ ppm/$CDCl_3$): 9.05 (s, 1H), 7.86 (s, 1H), 7.47 (d, *J* = 7.6 Hz, 1H), 7.33-7.28 (m, 3H), 7.22-7.16 (m, 5H), 3.83 (s, 3H), 1.60 (s, 3H). Enantiomeric excess was determined by HPLC on Chiralpak IC column (Hexane/Ethanol 90:10 *V*/*V*, flow rate 1.0 mL/min, 254 nm, τ_{minor} = 24.8 min, τ_{major} = 27.6 min, 63% ee). **HRMS** for $C_{17}H_{17}BrN_2NaO_4^+$: calcd. $[M+Na]^+$: 415.0264, found: 415.0252, $[M+2+Na]^+$: 417.0229.

(*S*)-Methyl 2-(hydroxy(phenyl)amino)-3-((3-methoxyphenyl)amino)-2-methyl-3-oxo-propanoate (4h)

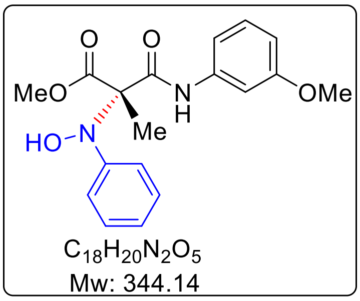

Following the general procedure, treatment of methyl 3-((3-methoxyphenyl)amino)-2-methyl-3-oxopropanoate (**1h**, 47 mg, 0.20 mmol) with nitrosobenzene (**2a**, 26 mg, 0.24 mmol) in the presence of (*R*,*R*)-TUC (**3a**, 17 mg, 0.04 mmol) in toluene (3 mL) at 0 °C for 3 h followed by column chromatography afforded the product **4h** as a red solid (52 mg, 75%). **R*f*** (EtOAc/hexane 3:7) = 0.30. **Mp** 135-137 °C. **^{13}C NMR** (100 MHz, δ ppm/$CDCl_3$): 171.7 (C), 166.8 (C), 160.4 (C), 147.1 (C), 138.7 (C), 129.8 (CH), 128.9 (CH), 126.0 (CH), 122.1 (CH), 122.1 (CH), 112.1 (CH), 111.0 (CH), 111.0 (CH), 105.5 (CH), 77.2 (C), 55.5 (CH_3), 53.5 (CH_3), 17.9 (CH_3). **^{1}H NMR** (400 MHz, δ ppm/$CDCl_3$): 9.01 (s, 1H), 7.36-7.16 (m, 8H), 7.03 (d, *J* = 7 .6 Hz, 1H), 6.70 (d, *J* = 7.6 Hz, 1H), 3.83 (s, 3H), 3.80 (s, 3H), 1.61 (s, 3H). Enantiomeric excess was determined by HPLC on Chiralpak IC column (Hexane/Ethanol 90:10 *V*/*V*, flow rate 1.0 mL/min, 254 nm, τ_{minor} = 35.3 min, τ_{major} = 37.5 min, 80% ee). **HRMS** for $C_{18}H_{20}N_2NaO_5^+$: calcd. $[M+Na]^+$: 367.1264, found: 367.1269.

(S)-Methyl 2-(hydroxy(phenyl)amino)-2-methyl-3-((3-nitrophenyl)amino)-3-oxopropan-oate (4i)

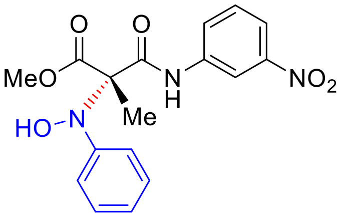

$C_{17}H_{17}N_3O_6$
Mw: 359.11

Following the general procedure, treatment of methyl 2-methyl-3-((3-nitrophenyl)amino)-3-oxopropanoate (**1i**, 51 mg, 0.20 mmol) with nitrosobenzene (**2a**, 26 mg, 0.24 mmol) in the presence of (R,R)-TUC (**3a**, 17 mg, 0.04 mmol) in toluene (3 mL) at 0 °C for 3 h followed by column chromatography afforded the product **4i** as a yellow solid (68 mg, 95%). **R_f** (EtOAc/hexane 3:7) = 0.33. **Mp** 155-157 °C. **^{13}C NMR** (100 MHz, δ ppm/$CDCl_3$): 171.3 (C), 167.7 (C), 148.8 (C), 146.8 (C), 138.6 (C), 130.0 (CH), 129.0 (CH), 129.0 (CH), 126.4 (CH), 125.7 (CH), 122.5 (CH), 122.5 (CH), 119.3 (CH), 114.9 (CH), 76.8 (C), 53.6 (CH_3), 18.4 (CH_3). **^{1}H NMR** (400 MHz, δ ppm/$CDCl_3$): 9.30 (s, 1H), 8.48 (s, 1H), 7.97 (t, J = 8.8 Hz, 2H), 7.51 (t, J= 8.0 Hz, 1H), 7.43 (s, 1H), 7.33-7.17 (m, 5H), 3.84 (s, 3H), 1.62 (s, 3H). Enantiomeric excess was determined by HPLC on Chiralpak IC column (Hexane/Ethanol 90:10 V/V, flow rate 1.0 mL/min, 254 nm, τ_{minor} = 19.6 min, τ_{major} = 20.3 min, 41% ee). **HRMS** for $C_{17}H_{17}N_3NaO_6{}^+$: calcd. [M+Na]$^+$: 382.1010, found: 382.1032.

(S)-Methyl 3-((2-bromophenyl)amino)-2-(hydroxy(phenyl)amino)-2-methyl-3-oxopropan-oate (4j)

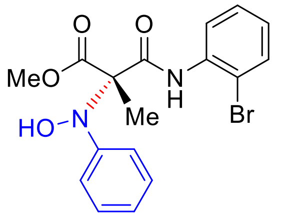

$C_{17}H_{17}BrN_2O_4$
Mw: 392.03

Following the general procedure, treatment of methyl 3-((2-bromophenyl)amino)-2-methyl-3-oxopropanoate (**1j**, 57 mg, 0.20 mmol) with nitrosobenzene (**2a**, 26 mg, 0.24 mmol) in the presence of (R,R)-TUC (**3a**, 17 mg, 0.04 mmol) in toluene (3 mL) at 0 °C for 3 h followed by column chromatography afforded the product **4j** as white solid (71 mg, 90%). **R_f** (EtOAc/hexane 3:7) = 0.34. **Mp** 125-127 °C. **^{13}C NMR** (100 MHz, δ ppm/$CDCl_3$): 171.1 (C), 167.1 (C), 147.1 (C), 135.6 (C), 132.5 (CH), 128.9 (CH), 128.9 (CH), 128.5 (CH), 126.3 (CH), 125.6 (CH), 122.8 (CH), 122.8 (CH), 122.0 (CH), 114.0 (C), 76.8 (C), 53.4 (CH_3), 18.0 (CH_3). **^{1}HNMR** (400 MHz, δ ppm/$CDCl_3$): 9.67 (s, 1H), 8.32 (d, J = 8 Hz, 1H), 7.57 (d, J = 8 Hz, 1H), 7.33-7.18 (m, 6H), 7.10 (s, 1H), 7.01 (t, J = 7.2 Hz, 1H), 3.84 (s, 3H), 1.63 (s, 3H). Enantiomeric excess was determined by HPLC on Chiralpak IC column (Hexane/Ethanol 90:10 V/V, flow rate 1.0 mL/min, 254 nm, τ_{minor} = 14.0 min, τ_{major} = 15.1 min, 54% ee). **HRMS** for $C_{17}H_{17}BrN_2NaO_4{}^+$: calcd. [M+Na]$^+$: 415.0264, found: 415.0242, [M+2+Na]$^+$: 417.0226.

(*S*)-Methyl 3-((2,5-dimethylphenyl)amino)-2-(hydroxy(phenyl)amino)-2-methyl-3-oxo-propanoate (1C less) (4k)

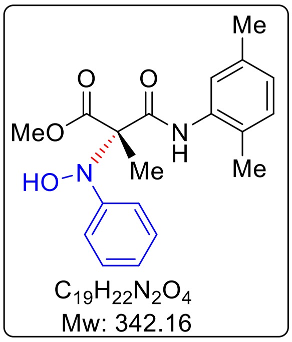

Following the general procedure, treatment of methyl 3-((2,5-dimethylphenyl)amino)-2-methyl-3-oxopropanoate (**1k**, 47 mg, 0.20 mmol) with nitrosobenzene (**2a**, 26 mg, 0.24 mmol) in the presence of (*R*,*R*)-TUC (**3a**, 17 mg, 0.04 mmol) in toluene (3 mL) at 0 °C for 3 h followed by column chromatography afforded the product **4k** as white solid (65 mg, 95%). **R*f*** (EtOAc/hexane 3:7) = 0.50. **Mp** 123-125 °C. **^{13}C NMR** (100 MHz, δ ppm/$CDCl_3$): 171.9 (C), 166.7 (C), 147.3 (C), 136.6 (C), 135.2 (C), 130.3 (CH), 128.8 (CH), 128.8 (CH), 125.9 (CH), 125.6 (CH), 122.7 (CH), 121.8 (CH), 121.8 (CH), 76.6 (C), 53.3 (CH_3), 21.2 (CH_3), 17.9 (CH_3), 17.1 (CH_3). **^{1}H NMR** (400 MHz, δ ppm/$CDCl_3$): 9.01 (s, 1H), 7.77 (s, 1H), 7.51 (s, 1H), 7.32-7.22 (m, 4H), 7.15 (t, *J* = 7.2 Hz, 1H), 7.08 (d, *J* = 7.6 Hz, 1H), 6.90 (d, *J* = 7.6 Hz, 1H), 3.82 (s, 3H), 2.32 (s, 3H), 2.23 (s, 3H), 1.66 (s, 3H). Enantiomeric excess was determined by HPLC on Chiralpak IC column (Hexane/Ethanol 95:5 *V*/*V*, flow rate 1.0 mL/min, 254 nm, τ_{minor} = 26.6 min, τ_{major} = 27.9 min, 85% ee). **HRMS** for $C_{19}H_{22}N_2NaO_4^+$: calcd. $[M+Na]^+$: 365.1472, found: 365.1445.

(*S*)-Methyl 3-((3,4-dimethoxyphenyl)amino)-2-(hydroxy(phenyl)amino)-2-methyl-3-oxo-propanoate (4l)

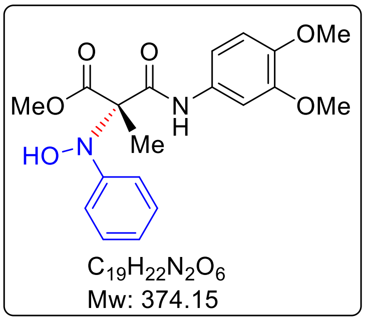

Following the general procedure, treatment of methyl 3-((3,4-dimethoxyphenyl)amino)-2-methyl-3-oxopropanoate (**1l**, 50 mg, 0.20 mmol) with nitrosobenzene (**2a**, 26 mg, 0.24 mmol) in the presence of (*R*,*R*)-TUC (**3a**, 17 mg, 0.04 mmol) in toluene (3 mL) at 0 °C for 4 h followed by column chromatography afforded the product **4l** as white solid (61 mg, 82%). **R*f*** (EtOAc/hexane 3:7) = 0.16. **Mp** 143-145 °C. **^{13}C NMR** (100 MHz, δ ppm/$CDCl_3$): 171.8 (C), 166.6 (C), 149.3 (C), 147.2 (C), 146.2 (C), 131.2 (C), 128.9 (CH), 128.9 (CH), 125.9 (CH), 122.1 (CH), 122.1 (CH), 111.8 (CH), 111.5 (CH), 104.7 (CH), 76.7 (C), 56.3 (CH_3), 56.1 (CH_3), 53.4 (CH_3), 17.9 (CH_3). **^{1}HNMR** (400 MHz, δ ppm/$CDCl_3$): 8.93 (s, 1H), 7.40-7.28 (m, 4H), 7.23-7.21 (m, 2H), 7.15 (t, *J* = 7.2 Hz, 1H), 6.94 (dd, *J*= 8.8 Hz, 2.4 Hz, 1H), 6.82 (d, *J*= 8.8 Hz, 1H), 3.88 (s, 3H), 3.86 (s, 3H), 3.83 (s, 3H), 1.61 (s, 3H). Enantiomeric excess was determined by HPLC on Chiralpak IC column (Hexane/Ethanol 90:10 *V*/*V*, flow rate 1.0 mL/min, 254 nm, τ_{minor} = 16.3 min, τ_{major} = 17.5 min, 91% ee). **HRMS** for $C_{19}H_{23}N_2O_6{}^+$: calcd. $[M+H]^+$: 375.1551, found: 375.1554.

(*S*)-Methyl 2-(hydroxy(phenyl)amino)-2-methyl-3-((4-methylthiazol-2-yl)amino)-3-oxo-propanoate (4m)

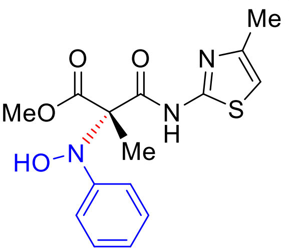

$C_{15}H_{17}N_3O_4S$
Mw: 335.09

Following the general procedure, treatment of methyl 2-methyl-3-((4-methylthiazol-2-yl)amino)-3-oxopropanoate (**1m**, 45 mg, 0.20 mmol) with nitrosobenzene (**2a**, 26 mg, 0.24 mmol) in the presence of (*R*,*R*)-TUC (**3a**, 17 mg, 0.04 mmol) in toluene (3 mL) at 0 °C for 3 h followed by column chromatography afforded the product **4m** as white solid (44 mg, 66%). **R*f*** (EtOAc/hexane 3:7) = 0.50. **Mp** 133-135 °C. **^{13}C NMR** (100 MHz, δ ppm/$CDCl_3$): 170.2 (C), 167.2 (C), 157.3 (C), 147.4 (C), 147.4 (C), 128.8 (CH), 128.8 (CH), 126.3 (CH), 122.9 (CH), 122.9 (CH), 108.7 (CH), 76.8 (C), 53.4 (CH_3), 17.5 (CH_3), 17.0 (CH_3). **^{1}H NMR** (400 MHz, δ ppm/$CDCl_3$): 7.26-7.20 (m, 3H), 7.16-7.15 (m, 2H), 7.10 (t, *J* = 5.8 Hz, 1H), 6.50 (s, 1H), 3.70 (s, 3H), 2.30 (s, 3H), 1.57 (s, 3H). Enantiomeric excess was determined by HPLC on Chiralpak IC column (Hexane/Ethanol 90:10 *V*/*V*, flow rate 1.0 mL/min, 254 nm, τ_{minor} = 9.1 min, τ_{major} = 9.8 min, 62% ee). **HRMS** for $C_{15}H_{18}N_3O_4S^+$: calcd. $[M+H]^+$: 336.1013, found: 336.1010.

(*S*)-Methyl 3-(*tert*-butylamino)-2-(hydroxy(phenyl)amino)-2-methyl-3-oxopropanoate (4n)

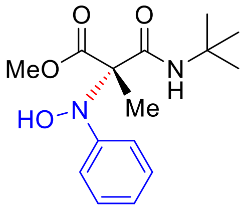

$C_{15}H_{22}N_2O_4$
Mw: 294.16

Following the general procedure, treatment of methyl 3-(*tert*-butylamino)-2-methyl-3-oxopropanoate (**1n**, 34 mg, 0.20 mmol) with nitrosobenzene (**2a**, 26 mg, 0.24 mmol) in the presence of (*R*,*R*)-TUC (**3a**, 17 mg, 0.04 mmol) in toluene (3 mL) at 0 °C for 3 h followed by column chromatography afforded the product **4n** as white solid (50 mg, 85%). **R*f*** (EtOAc/hexane 3:7) = 0.50. **Mp** 144-146 °C. **^{13}C NMR** (100 MHz, δ ppm/$CDCl_3$): 172.4 (C), 167.9 (C), 147.5 (C), 128.6 (CH), 128.6 (CH), 125.0 (CH), 121.3 (CH), 121.3 (CH), 76.1 (C), 53.1 (CH_3), 51.5 (C), 28.5 (CH_3), 28.5 (CH_3), 28.5 (CH_3), 18.2 (CH_3). **^{1}H NMR** (400 MHz, δ ppm/$CDCl_3$): 7.42 (s, 1H), 7.30-7.26 (m, 2H), 7.18-7.16 (m, 2H), 7.11 (t, *J* = 7.2 Hz, 1H), 6.98 (s, 1H), 3.80 (s, 3H), 1.56 (s, 3H), 1.36 (s, 9H). Enantiomeric excess was determined by HPLC on Chiralpak IC column (Hexane/Ethanol 90:10 *V*/*V*, flow rate 1.0 mL/min, 254 nm, τ_{minor} = 13.6 min, τ_{major} = 14.5 min, 71% ee). **HRMS** for $C_{15}H_{23}N_2O_4^+$: calcd. $[M+H]^+$: 295.1652, found: 295.1653.

(*S*)-Ethyl 3-((4-bromophenyl)amino)-2-(hydroxy(phenyl)amino)-2-methyl-3-oxopropan-oate (4o)

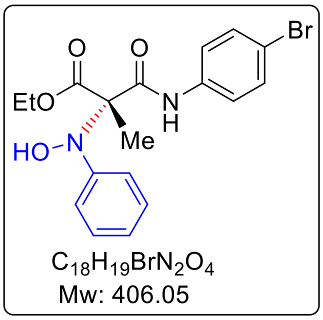

Following the general procedure, treatment of ethyl 3-((4-bromophenyl)amino)-2-methyl-3-oxopropanoate (**1o**, 60 mg, 0.20 mmol) with nitrosobenzene (**2a**, 26 mg, 0.24 mmol) in the presence of (*R*,*R*)-TUC (**3a**, 17 mg, 0.04 mmol) in toluene (3 mL) at 0 °C for 3 h followed by column chromatography afforded the product **4o** as white solid (54 mg, 66%). $\mathbf{R_f}$ (EtOAc/hexane 3:7) = 0.22. **Mp** 122-124 °C. ^{13}**C NMR** (100 MHz, δ ppm/$CDCl_3$): 171.0 (C), 167.0 (C), 147.2 (C), 136.5 (C), 132.1 (CH), 132.1 (CH), 128.8 (CH), 128.8 (CH), 126.0 (CH), 122.1 (CH), 122.1 (CH), 121.6 (CH), 121.6 (CH), 117.3 (C), 76.8 (C), 62.8 (CH_2), 17.6 (CH_3), 14.0 (CH_3). ^{1}**H NMR** (400 MHz, δ ppm/$CDCl_3$): 9.02 (s, 1H), 7.50-7.44 (m, 4H), 7.32-7.26 (m, 2H), 7.22-7.16 (m, 3H), 4.31 (q, *J* = 7.2 Hz, 2H), 1.60 (s, 3H), 1.29 (t, *J* = 7.2 Hz, 3H). Enantiomeric excess was determined by HPLC on Chiralpak IC column (Hexane/Ethanol 95:5 *V*/*V*, flow rate 1.0 mL/min, 254 nm, τ_{minor} = 10.6 min, τ_{major} = 13.2 min, 76% ee). **HRMS** for $C_{18}H_{19}BrN_2NaO_4^+$: calcd. $[M+Na]^+$: 429.0420, found: 429.0476, $[M+2+Na]^+$: 431.0422.

(*S*)-Ethyl 3-((3,4-dimethoxyphenyl)amino)-2-(hydroxy(phenyl)amino)-2-methyl-3-oxo-propanoate (4p)

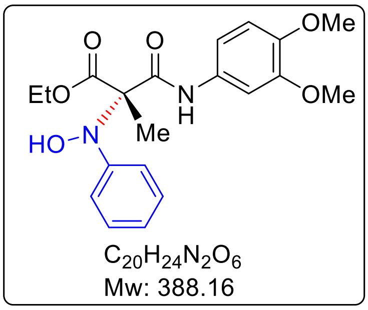

Following the general procedure, treatment of ethyl 3-((3,4-dimethoxyphenyl)amino)-2-methyl-3-oxopropanoate (**1p**, 56 mg, 0.20 mmol) with nitrosobenzene (**2a**, 26 mg, 0.24 mmol) in the presence of (*R*,*R*)-TUC (**3a**, 17 mg, 0.04 mmol) in toluene (3 mL) at 0 °C for 3 h followed by column chromatography afforded the product **4p** as white solid (51 mg, 66%). $\mathbf{R_f}$ (EtOAc/hexane 3:7) = 0.22. **Mp** 132-134 °C. ^{13}**C NMR** (100 MHz, δ ppm/$CDCl_3$): 171.3 (C), 166.7 (C), 149.2 (C), 147.3 (C), 146.2 (C), 131.2 (C), 128.8 (CH), 128.8 (CH), 125.8 (CH), 122.0 (CH), 122.0 (CH), 111.8 (CH) 111.5 (CH), 104.7 (CH), 76.6 (C), 62.8 (CH_2), 56.2 (CH_3), 56.0 (CH_3), 17.7 (CH_3), 14.0 (CH_3). ^{1}**H NMR** (400 MHz, δ ppm/$CDCl_3$): 8.91 (s, 1H), 7.39 (d, *J* = 2.0 Hz, 1H), 7.34-7.29 (m, 3H), 7.24-7.22 (m, 2H), 7.16 (t, *J* = 5.8 Hz, 1H), 6.96 (dd, *J* = 6.8, 2.0 Hz, 1H), 6.83 (d, *J* = 6.8 Hz, 1H), 4.32-4.30 (m, 2H), 3.90 (s, 3H), 3.87 (s, 3H), 1.62 (s, 3H), 1.30 (t, *J* = 5.8 Hz, 3H). Enantiomeric excess was determined by HPLC on Chiralpak IC column (Hexane/Ethanol 90:10 *V*/*V*, flow rate 1.0 mL/min, 254 nm, τ_{minor} = 36.3 min, τ_{major} = 40.1 min, 80% ee). **HRMS** for $C_{20}H_{24}N_2NaO_6^+$: calcd. $[M+Na]^+$: 411.1527, found: 411.1540.

(*S*)-Ethyl 2-(hydroxy(phenyl)amino)-2-methyl-3-((4-nitrophenyl)amino)-3-oxopropanoate (4q)

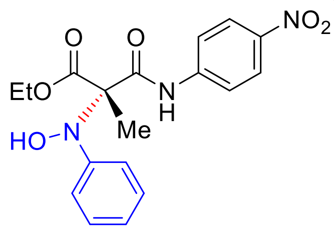

$C_{18}H_{19}N_3O_6$
Mw: 373.13

Following the general procedure, treatment of ethyl 2-methyl-3-((4-nitrophenyl)amino)-3-oxopropanoate (**1q**, 53 mg, 0.20 mmol) with nitrosobenzene (**2a**, 26 mg, 0.24 mmol) in the presence of (*R*,*R*)-TUC (**3a**, 17 mg, 0.04 mmol) in toluene (3 mL) at 0 °C for 3 h followed by column chromatography afforded the product **4q** as white solid (52 mg, 70%). **R*f*** (EtOAc/hexane 3:7) = 0.22. **Mp** 115-117 °C. **^{13}C NMR** (100 MHz, δ ppm/$CDCl_3$): 170.7 (C), 167.8 (C), 146.9 (C), 143.9 (C), 143.3 (C), 129.0 (CH), 129.0 (CH), 126.4 (CH), 125.2 (CH), 125.2 (CH), 122.5 (CH), 122.5 (CH), 119.5 (CH) 119.5 (CH), 76.8 (C), 63.0 (CH_2), 18.0 (CH_3), 14.0 (CH_3). **^{1}H NMR** (400 MHz, δ ppm/$CDCl_3$): 9.42 (s, 1H), 8.20 (d, *J* =8.8 Hz, 2H), 7.74 (d, *J* = 8.8 Hz, 2H), 7.60 (s, 1H), 7.32-7.26 (m, 2H), 7.22-7.16 (m, 3H), 4.29 (Q, *J* = 7.0 Hz, 2H), 1.60 (s, 3H), 1.28 (t, *J* = 7.2 Hz, 3H). Enantiomeric excess was determined by HPLC on Chiralpak IC column (Hexane/Ethanol 90:10 *V*/*V*, flow rate 1.0 mL/min, 254 nm, τ_{minor} = 12.8 min, τ_{major} = 24.8 min, 67% ee). **HRMS** for $C_{18}H_{19}N_3NaO_6^+$: calcd. $[M+Na]^+$: 396.1166, found: 396.1168.

(*S*)-*tert*-Butyl 3-((3,4-dimethoxyphenyl)amino)-2-(hydroxy(phenyl)amino)-2-methyl-3-oxopropanoate (4r)

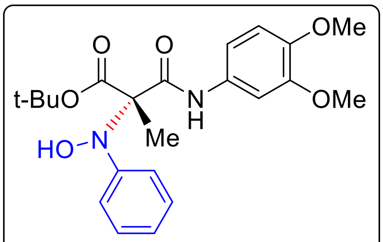

$C_{22}H_{28}N_2O_6$
Mw: 416.19

Following the general procedure, treatment of *t*-butyl 3-((3,4-dimethoxyphenyl)amino)-2-methyl-3-oxopropanoate (**1r**, 62 mg, 0.20 mmol) with nitrosobenzene (**2a**, 26 mg, 0.24 mmol) in the presence of (*R*,*R*)-TUC (**3a**, 17 mg, 0.04 mmol) in toluene (3 mL) at 0 °C for 6 h followed by column chromatography afforded the product **4r** as a red solid (42 mg, 50%). **R*f*** (EtOAc/hexane 3:7) = 0.28. **Mp** 96-98 °C. **^{13}C NMR** (100 MHz, δ ppm/$CDCl_3$): 170.6 (C), 167.2 (C), 149.3 (C), 147.5 (C), 146.2 (C), 131.3 (C), 128.8 (CH), 128.8 (CH), 125.7 (CH), 122.2 (CH), 122.2 (CH), 112.0 (CH), 111.6 (CH), 104.9 (CH), 84.5 (C), 77.2 (C), 56.3 (CH_3), 56.1 (CH_3), 28.0 (CH_3), 28.0 (CH_3), 28.0 (CH_3), 18.3 (CH_3). **^{1}H NMR** (400 MHz, δ ppm/$CDCl_3$): 8.90 (s, 1H), 7.54 (s, 1H), 7.36-7.26 (m, 5H), 7.15 (t, *J* =7.2 Hz, 1H) ,6.96 (dd, *J* = 8.4, 2.4 Hz, 1H), 6.82 (d, *J* = 8.4 Hz, 1H), 3.89 (s, 3H), 3.86 (s, 3H), 1.56 (s, 3H), 1.50 (s, 9H). Enantiomeric excess was determined by HPLC on Chiralpak IC column (Hexane/Ethanol 90:10 *V*/*V*, flow rate 1.0 mL/min, 254 nm, τ_{minor} = 24.0 min, τ_{major} = 24.9 min, 84% ee). **HRMS** for $C_{22}H_{29}N_2O_6$: calcd. [M+H]+: 417.2020, found: 417.2018

(*S*)-*tert*-Butyl 2-(hydroxy(phenyl)amino)-2-methyl-3-((4-nitrophenyl)amino)-3-oxopropanoate (4s)

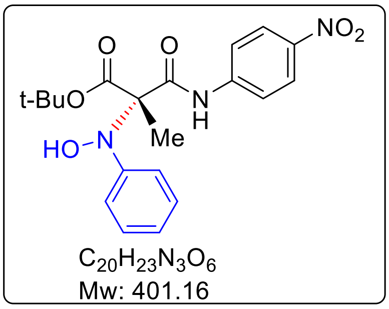

Following the general procedure, treatment of *tert*-butyl 2-methyl-3-((4-nitrophenyl)amino)-3-oxopropanoate (**1s**, 59 mg, 0.20 mmol) with nitrosobenzene (**2a**, 26 mg, 0.24 mmol) in the presence of (*R*,*R*)-TUC (**3a**, 17 mg, 0.04 mmol) in toluene (3 mL) at 0 °C for 3 h followed by column chromatography afforded the product **4s** as a yellow solid (48 mg, 60%). **R*f*** (EtOAc/hexane 3:7) = 0.23. **Mp** 115-117 °C. **^{13}C NMR** (100 MHz, δ ppm/$CDCl_3$): 170.0 (C), 168.3 (C), 146.9 (C), 143.9 (C), 143.4 (C), 128.9 (CH), 128.9 (CH), 126.4 (CH), 125.2 (CH), 125.2 (CH), 122.6 (CH), 122.6 (CH), 119.5 (CH), 119.5 (CH), 85.0 (C), 77.2 (C), 28.0 (CH_3), 28.0 (CH_3), 28.0 (CH_3), 18.8 (CH_3). **^{1}H NMR** (400 MHz, δ ppm/$CDCl_3$): 9.36 (s, 1H), 8.24 (d, *J* = 8.8 Hz, 2H), 7.76 (d, J = 9.2 Hz, 2H), 7.73 (s, 1H), 7.32 (t, *J* = 7.8 Hz, 2H*),* 7.25-7.18 (m, 3H), 1.56 (s, 3H), 1.50 (s, 9H). Enantiomeric excess was determined by HPLC on Chiralpak IC column (Hexane/Ethanol 95:5 *V*/*V*, flow rate 1.0 mL/min, 254 nm, τ_{minor} = 11.3 min, τ_{major} = 12.5 min, 82% ee). **HRMS** for $C_{20}H_{23}N_3NaO_6$: calcd. $[M+Na]^+$: 424.1479, found: 424.1486.

(*S*)-4-Methoxybenzyl 3-((4-bromophenyl)amino)-2-(hydroxy(phenyl)amino)-2-methyl-3-oxopropanoate (4t)

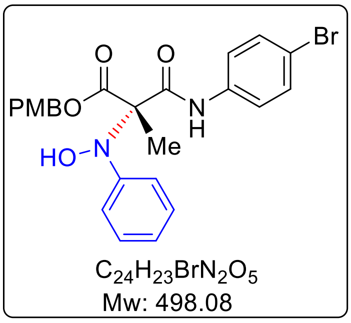

Following the general procedure, treatment of 4-methoxybenzyl 3-((4-bromophenyl)amino)-2-methyl-3-oxopropanoate (**1t**, 78 mg, 0.20 mmol) with nitrosobenzene (**2a**, 26 mg, 0.24 mmol) in the presence of (*R*,*R*)-TUC (**3a**, 17 mg, 0.04 mmol) in toluene (3 mL) at 0 °C for 3 h followed by column chromatography afforded the product **4t** as white solid (75 mg, 75%). **R*f*** (EtOAc/hexane 3:7) = 0.22. **Mp** 145-147 °C. **^{13}C NMR** (100 MHz, δ ppm/$CDCl_3$): 170.6 (C), 166.7 (C), 160.0 (C), 147.1 (C), 136.5 (C), 132.1 (CH), 132.1 (CH), 130.3 (CH), 130.3 (CH), 128.9 (CH), 128.9 (CH), 126.8 (C), 125.9 (CH), 122.0 (CH), 122.0 (CH), 121.6 (CH), 121.6 (CH), 117.3 (C), 114.1 (CH), 114.1 (CH), 77.0 (C), 68.2 (CH_2), 55.4 (CH_3), 17.4 (CH_3). **^{1}H NMR** (400 MHz, δ ppm/$CDCl_3$): 8.94 (s, 1H), 7.44-7.37 (m, 4H), 7.26-7.22 (m, 5H), 7.14-7.13 (m, 3H), 6.82 (d, *J* = 8.4 Hz, 2H), 5.23-5.16 (m, 2H), 3.79 (s, 3H), 1.58 (s, 3H). Enantiomeric excess was determined by HPLC on Chiralpak IC column (Hexane/Ethanol 95:5 *V*/*V*, flow rate 1.0 mL/min, 254 nm, τ_{minor} = 7.5 min, τ_{major} = 8.2 min, 82% ee). **HRMS** for $C_{24}H_{23}BrN_2NaO_5^+$: calcd. $[M+Na]^+$: 521.0683, found: 521.0705, $[M+2+Na]^+$: 415.0264.

(*S*)-4-Methoxybenzyl 2-(hydroxy(phenyl)amino)-3-((4-methoxyphenyl)amino)-2-methyl-3-oxopropanoate (4u)

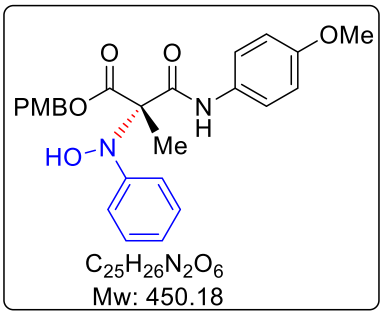

Following the general procedure, treatment of 4-methoxybenzyl 3-((4-methoxyphenyl)amino)-2-methyl-3-oxopropanoate (**1u**, 69 mg, 0.20 mmol) with nitrosobenzene (**2a**, 26 mg, 0.24 mmol) in the presence of (*R*,*R*)-TUC (**3a**, 17 mg, 0.04 mmol) in toluene (3 mL) at 0 °C for 3 h followed by column chromatography afforded the product **4u** as white solid (63 mg, 70%). **R_f** (EtOAc/hexane 3:7) = 0.22. **Mp** 155-157 °C. **^{13}C NMR** (100 MHz, δ ppm/$CDCl_3$): 170.7 (C), 166.3 (C), 159.9 (C), 156.6 (C), 147.3 (C), 130.5 (C), 130.2 (CH), 130.2 (CH), 128.7 (CH), 128.7 (CH), 126.9 (C), 125.5 (CH), 121.7 (CH), 121.7 (CH), 121.7 (CH), 121.7 (CH), 114.2 (CH), 114.2 (CH) 114.0 (CH), 114.0 (CH), 76.8 (C), 68.0 (CH_2), 55.5 (CH_3), 55.3 (CH_3) 17.2 (CH_3). **^{1}H NMR** (400 MHz, δ ppm/$CDCl_3$): 8.85 (s, 1H), 7.47-7.42 (m, 2H), 7.26-7.14 (m, 8H), 6.89-6.85 (m, 4H), 5.27-5.20 (m, 2H), 3.82 (s, 6H), 1.63 (s, 3H). Enantiomeric excess was determined by HPLC on Chiralpak IC column (Hexane/Ethanol 90:10 *V*/*V*, flow rate 1.0 mL/min, 254 nm, τ_{minor} = 15.6 min, τ_{major} = 16.9 min, 81% ee). **HRMS** for $C_{25}H_{27}N_2O_6^+$: calcd. $[M+H]^+$: 451.1864, found: 451.1855.

(*S*)-3-Methoxybenzyl 3-((4-bromophenyl)amino)-2-(hydroxy(phenyl)amino)-2-methyl-3-oxopropanoate (4v)

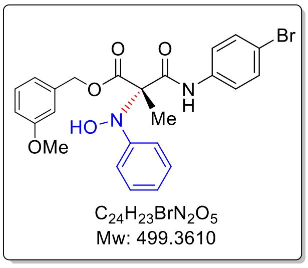

Following the general procedure, treatment of 3-methoxybenzyl 3-((4-bromophenyl)amino)-2-methyl-3-oxopropanoate (**1v**, 78 mg, 0.20 mmol) with nitrosobenzene (**2a**, 26 mg, 0.24 mmol) in the presence of (*R*,*R*)-TUC (**3a**, 17 mg, 0.04 mmol) in toluene (3 mL) at 0 °C for 3 h followed by column chromatography afforded the product **4v** as white solid (77 mg, 77%). **R_f** (EtOAc/hexane 3:7) = 0.22. **Mp** 135-137 °C. **^{13}C NMR** (100 MHz, δ ppm/$CDCl_3$): 170.6 (C), 166.8 (C), 159.9 (C), 147.1 (C), 136.5 (C), 136.3 (C), 132.1 (CH), 132.1 (CH), 129.9 (CH), 128.9 (CH), 128.9 (CH), 126.0 (CH), 122.1 (CH), 122.1 (CH), 121.7 (CH), 121.7 (CH), 120.4 (CH), 117.4 (C), 114.3 (CH), 113.6 (CH), 77.0 (C), 68.1 (CH_2), 55.3 (CH_3), 17.5 (CH_3). **^{1}H NMR** (400 MHz, δ ppm/$CDCl_3$): 9.00 (s, 1H), 7.44-7.39 (m, 4H), 7.30 (s, 1H), 7.25-7.22 (m, 3H), 7.16-7.13 (m, 3H), 6.87-6.82 (m, 3H), 5.20 (d, *J* = 2.4 Hz, 2H), 3.73 (s, 3H), 1.61 (s, 3H). Enantiomeric excess was determined by HPLC on Chiralpak IA column (Hexane/Ethanol 90:10 *V*/*V*, flow rate 1.0 mL/min, 235 nm), minor enantiomer t_r = 15.9 min, major enantiomer t_r = 13.8 min, 83% ee. **HRMS** for $C_{24}H_{24}BrN_2O_5^+$: calcd. $[M+H]^+$: 499.0863, found: 499.0873, $[M+2+H]^+$: 501.0848.

(*S*)-3,4,5-Trimethoxybenzyl 3-((4-bromophenyl)amino)-2-(hydroxy(phenyl)amino)-2-methyl-3-oxopropanoate (4w)

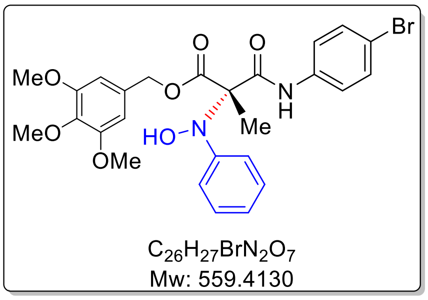

Following the general procedure, treatment of 3,4,5-trimethoxybenzyl 3-((4-bromophenyl)amino)-2-methyl-3-oxopropanoate (**1w**, 90.0 mg, 0.20 mmol) with nitrosobenzene (**2a**, 26 mg, 0.24 mmol) in the presence of (*R*,*R*)-TUC (**3a**, 17 mg, 0.04 mmol) in toluene (3 mL) at 0 °C for 3 h followed by column chromatography afforded the product **4w** as white solid (73 mg, 65%). **R*f*** (EtOAc/hexane 3:7) = 0.10. **Mp** 140-142 °C. **^{13}C NMR** (100 MHz, δ ppm/$CDCl_3$): 170.4 (C), 166.9 (C), 153.3 (C), 153.3 (C), 153.3 (C), 147.1 (C), 138.1 (C), 136.5 (C), 132.1 (CH), 132.1 (CH), 132.1 (CH), 130.4 (C), 128.8 (CH), 125.9 (CH), 122.1 (CH), 121.5 (CH), 121.5 (CH), 117.3 (CH), 105.2 (CH), 105.2 (CH), 77.2 (C), 68.2 (CH_2), 60.9 (CH_3), 56.1 (CH_3), 56.1 (CH_3), 17.7 (CH_3). **^{1}H NMR** (400 MHz, δ ppm/$CDCl_3$): 9.05 (s, 1H), 7.42 (s, 4H), 7.24-7.20 (m, 2H), 7.16-7.14 (m, 3H), 6.49 (s, 2H), 5.17 (s, 2H), 3.81 (s, 3H), 3.75 (s, 6H), 1.62 (s, 3H). Enantiomeric excess was determined by HPLC on Chiralpak IA column (Hexane/Ethanol 90:10 *V*/*V*, flow rate 1.0 mL/min, 235 nm), minor enantiomer t_r = 19.4 min, major enantiomer t_r = 16.8 min, 39% ee. **HRMS** for $C_{26}H_{28}BrN_2O_7^+$: calcd. $[M+Na]^+$: 581.0894, found: 581.0901, $[M+2+Na]^+$: 583.0881.

(*S*)-Methyl 3-((4-bromophenyl)amino)-2-(hydroxy(*o*-tolyl)amino)-2-methyl-3-oxopropanoate (4x)

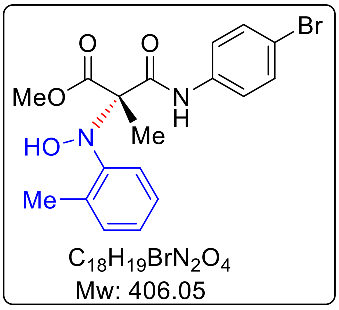

Following the general procedure, treatment of methyl 3-((4-bromophenyl)amino)-2-methyl-3-oxopropanoate (**1a**, 57 mg, 0.20 mmol) with 2-methylnitrosobenzene (**2b**, 29 mg, 0.24 mmol) in the presence of (*R*,*R*)-TUC (**3a**, 17 mg, 0.04 mmol) in toluene (3 mL) at 0 °C for 3 h followed by column chromatography afforded the product **4x** as white solid (25 mg, 31%). **R*f*** (EtOAc/hexane 3:7) = 0.50. **Mp** 145-147 °C. **^{13}C NMR** (100 MHz, δ ppm/$CDCl_3$): 173.2 (C), 168.7 (C), 144.2 (C), 136.7 (C), 132.2 (CH), 132.2 (CH), 131.6 (C), 128.1 (CH), 127.0 (CH), 123.0 (CH), 123.0 (C), 121.7 (CH), 121.7 (CH), 117.3 (CH), 74.7 (C), 53.4 (CH_3), 20.9 (CH_3), 18.8 (CH_3). **^{1}H NMR** (400 MHz, δ ppm/$CDCl_3$): 9.22 (s, 1H), 7.81 (s, 1H), 7.54-7.39 (m, 4H), 7.26-7.16 (m, 4H), 3.93 (s, 3H), 2.49 (s, 3H), 1.51 (s, 3H). Enantiomeric excess was determined by HPLC on Chiralpak IC column (Hexane/Ethanol 95:5 *V*/*V*, flow rate 1.0 mL/min, 254 nm, τ_{minor} = 6.6 min, τ_{major} = 8.4 min, 30% ee). **HRMS** for $C_{18}H_{19}BrN_2NaO_4$: calcd. $[M+Na]^+$: 429.0420, found: 429.0410, $[M+2+Na]^+$: 431.0264.

(*S*)-Methyl 3-((3,4-dimethoxyphenyl)amino)-2-(hydroxy(*o*-tolyl)amino)-2-methyl-3-oxopropanoate (4y)

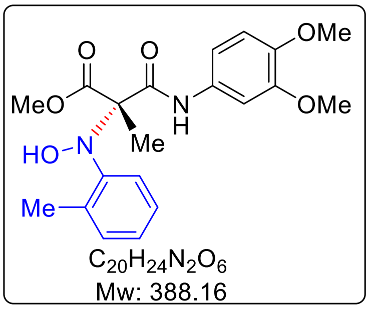

Following the general procedure, treatment of methyl 3-((3,4-dimethoxyphenyl)amino)-2-methyl-3-oxopropanoate (**1l**, 57 mg, 0.20 mmol) with 2-methylnitrosobenzene (**2b**, 29 mg, 0.24 mmol) in the presence of (*R*,*R*)-TUC (**3a**, 17 mg, 0.04 mmol) in toluene (3 mL) at 0 °C for 4 h followed by column chromatography afforded the product **4y** (27 mg, 35%). **R*f*** (EtOAc/hexane 3:7) = 0.42. **Mp** 129-131 °C. **^{13}C NMR** (100 MHz, δ ppm/$CDCl_3$): 173.4 (C), 168.4 (C), 149.4 (C), 146.2 (C), 144.4 (C), 136.8 (C), 131.5 (CH), 131.4 (C), 127.9 (CH), 127.0 (CH), 123.0 (CH), 111.9 (CH), 111.6 (CH), 104.9 (CH), 74.7 (C), 56.3 (CH_3), 56.1 (CH_3), 53.3 (CH_3), 21.0 (CH_3), 18.8 (CH_3). **^{1}H NMR** (400 MHz, δ ppm/$CDCl_3$): 9.12 (s, 1H), 7.84 (s, 1H), 7.49 (s, 1H), 7.26-7.18 (m, 4H), 6.91-6.83 (m, 2H), 3.94 (s, 3H), 3.91 (s, 3H), 3.87 (s, 3H), 2.50 (s, 3H), 1.52 (s, 3H). Enantiomeric excess was determined by HPLC on Chiralpak IC column (Hexane/Ethanol 90:10 *V*/*V*, flow rate 1.0 mL/min, 254 nm, τ_{minor} = 16.5 min, τ_{major} = 23.0 min, 30% ee). **HRMS** for $C_{20}H_{24}N_2NaO_6^+$: calcd. $[M+H]^+$: 411.1527, found: 411.1527.

Synthesis of methyl-3-((4-bromophenyl)amino)-2-methyl-3-oxo-2-(phenylamino)propan-oate (5)

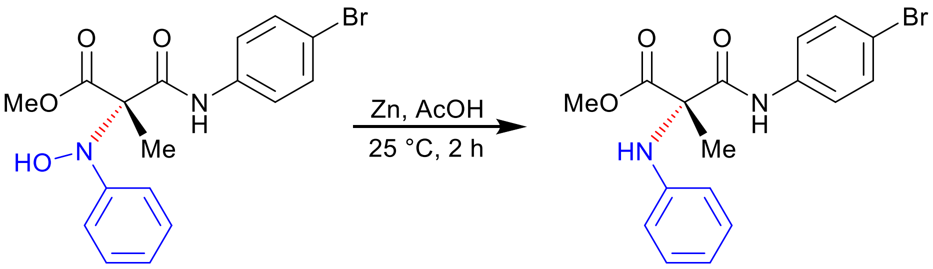

4a, 90% ee

5, 93%, 49% ee

To a 10 mL round-bottomed flask charged with **4a** (78 mg, 0.20 mmol, 1 equiv) were added Zn dust (260 mg, 4.0 mmol, 20 equiv) and acetic acid (2 mL). This reaction mixture was stirred at 25 °C for 2 h. After the completion of the reaction, as indicated by TLC, the reaction was quenched with water and extracted with ethyl acetate. The combined organic layer was washed with saturated solution of $NaHCO_3$ and brine. After drying over anhydrous Na_2SO_4 the solvent was evaporated under reduced pressure. The residue was purified by column chromatography (100–200 mesh silica gel) using ethyl acetate/hexane as the eluent to afford compound **5** as white solid (70 mg, 93%). **R*f*** (ethyl acetate/hexane 15:85) = 0.35. **Mp** 183-185 °C. **^{13}C NMR** (100 MHz, δ ppm/$CDCl_3$): 172.1 (C), 167.6 (C), 142.9 (C), 136.5 (C), 132.1 (CH), 132.1(CH), 129.6 (CH), 129.6 (CH), 121.5 (CH), 121.5 (CH), 120.5 (CH), 117.4 (C), 116.6 (CH), 116.6 (CH), 66.0 (C), 54.1 (CH_3), 18.9 (CH_3). **^{1}H NMR** (400 MHz, δ ppm/$CDCl_3$): 8.90 (s, 1H), 7.42 (s, 4H), 7.20 (t, *J* = 8.0 Hz, 2H), 6.90 (t, *J* = 7.2 Hz, 1H), 6.65 (d, *J* = 7.6 Hz,

2H), 5.12 (s, 1H), 3.85 (s, 3H), 1.76 (s, 3H). Enantiomeric excess was determined by HPLC on Chiralpak IA column (Hexane/Ethanol 92:8 *V/V*, flow rate 1.0 mL/min, 251 nm, τ_{minor} = 24.9 min, τ_{major} = 15.9 min, 49% ee). **HRMS** for $C_{17}H_{18}BrN_2O_3^+$: calcd. $[M+H]^+$: 377.0495, found: 377.0498, $[M+2+H]^+$: 379.0477.

X-ray data of compound 4a

X-ray data collection and structure refinement details of compound 4a

A good quality colorless single crystal of size 0.28 × 0.22 × 0.19 mm, was selected under a polarizing microscope and mounted on a glass fiber for data collection. Single crystal X-ray data for compound **4a** were collected on a Rigaku Kappa 3 circle diffractometer equipped with the AFC12 goniometer and enhanced sensitivity (HG) Saturn724+ CCD detector in the 4 × 4 bin mode using the monochromated MoKα radiation generated from the microfocus sealed tube MicroMax-003 X-ray generator equipped with specially designed confocal multilayer optics. Data collection was performed using ω-scans of 0.5° steps at 293(2) K. Cell determination, data collection, and data reduction was performed using the Rigaku CrystalClear-SM Expert 2.1 b24[2] software. Structure solution and refinement were performed by using SHELXTL-NT.[3] Refinement of coordinates and anisotropic thermal parameters of non-hydrogen atoms were carried out by the full-matrix least-squares method. The hydrogen atoms attached to carbon atoms were generated with idealized geometries and isotropically refined using a riding model.

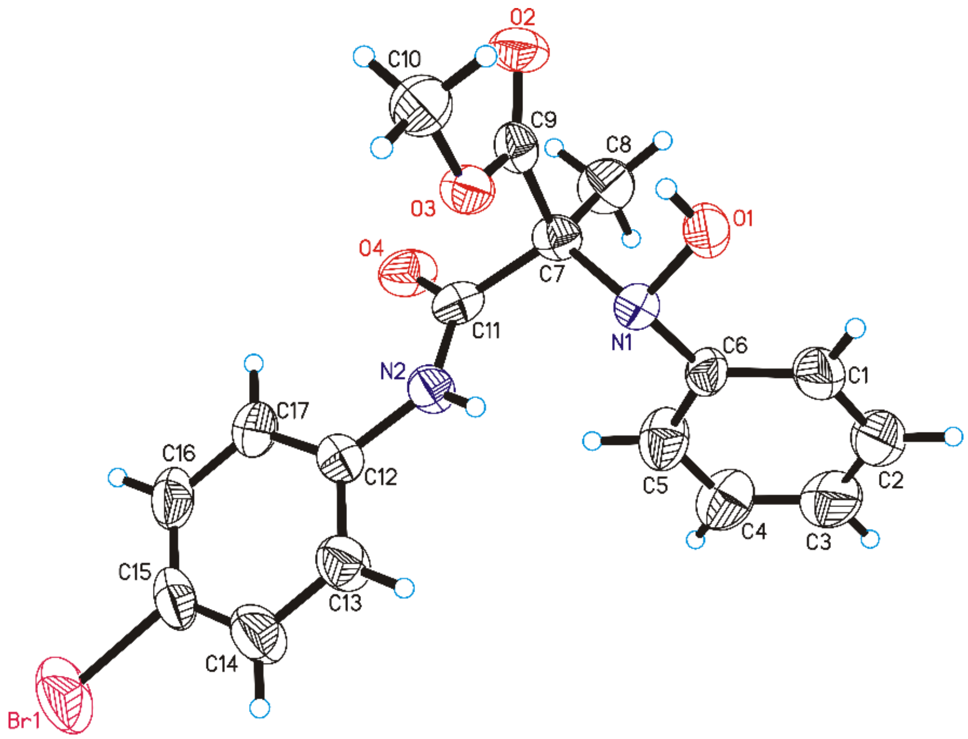

Figure S1: ORTEP diagram drawn with 30% ellipsoid probability for non-H atoms of the crystal structure of chiral compound **4a** determined at 293 K. The absolute configuration of C7 is *S*.

Table S1: Crystal data and structure refinement details for compound **4a**.

Compound	4a
Empirical formula	$C_{17} H_{17} Br N_2 O_4$
Formula weight	393.24
Crystal System	Orthorhombic
Space group	$P\ 2_12_12_1$
a (Å)	10.151(3)
b (Å)	12.755(3)
c (Å)	14.560(4)
α (°)	90.00
β (°)	90.00
γ (°)	90.00
V (Å^3)	1885.2(9)
Z	4
D_c (g/cm^3)	1.386
F_{000}	800
μ (mm^{-1})	2.201
θ_{max} (°)	25.36
Total reflections	12849
Unique reflections	3413
Reflections [$I > 2\sigma(I)$]	1773
Parameters	217
R_{int}	0.0488
Goodness-of-fit	0.959
R [$F^2 > 2\sigma(F^2)$]	0.0488
wR (F^2, all data)	0.1242
CCDC No.	1898632

References

1. R. A. Dhokale, P. R. Thakare, S. B. Mhaske, *Org. Lett.* **2012**, *14*, 3994.
2. CrystalClear 2.1, Rigaku Corporation, Tokyo, Japan.
3. G. M. Sheldrick, *Acta Crystallogr., Sect. A* 2008, **64**, 112.

Copies of ^{1}H and ^{13}C NMR spectra for all compounds

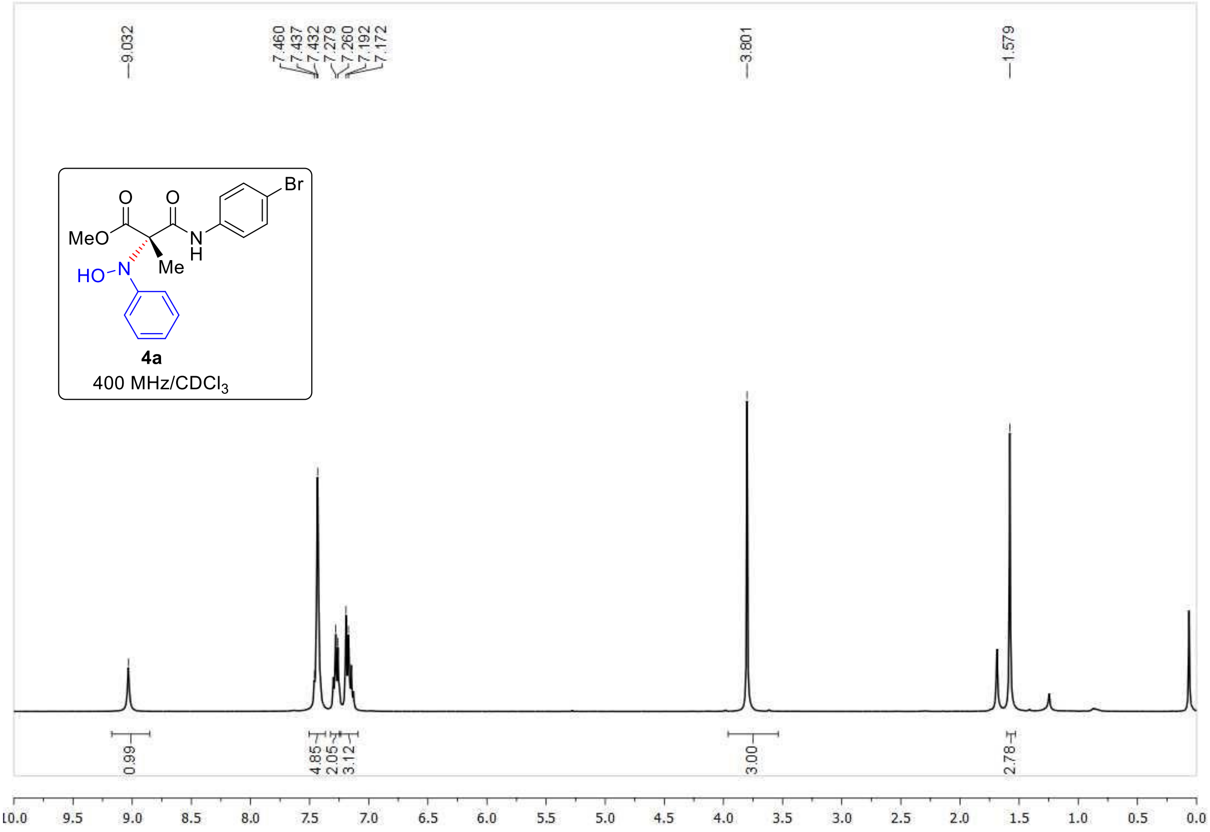

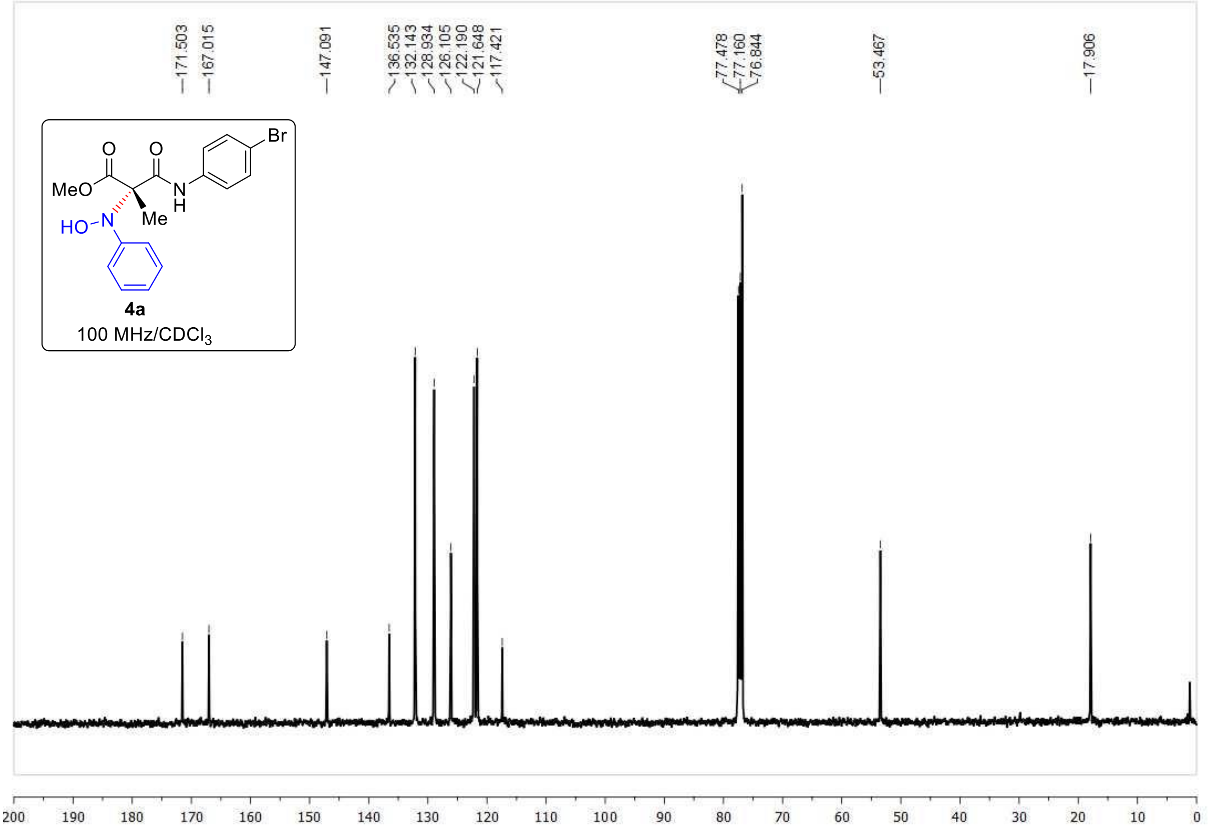

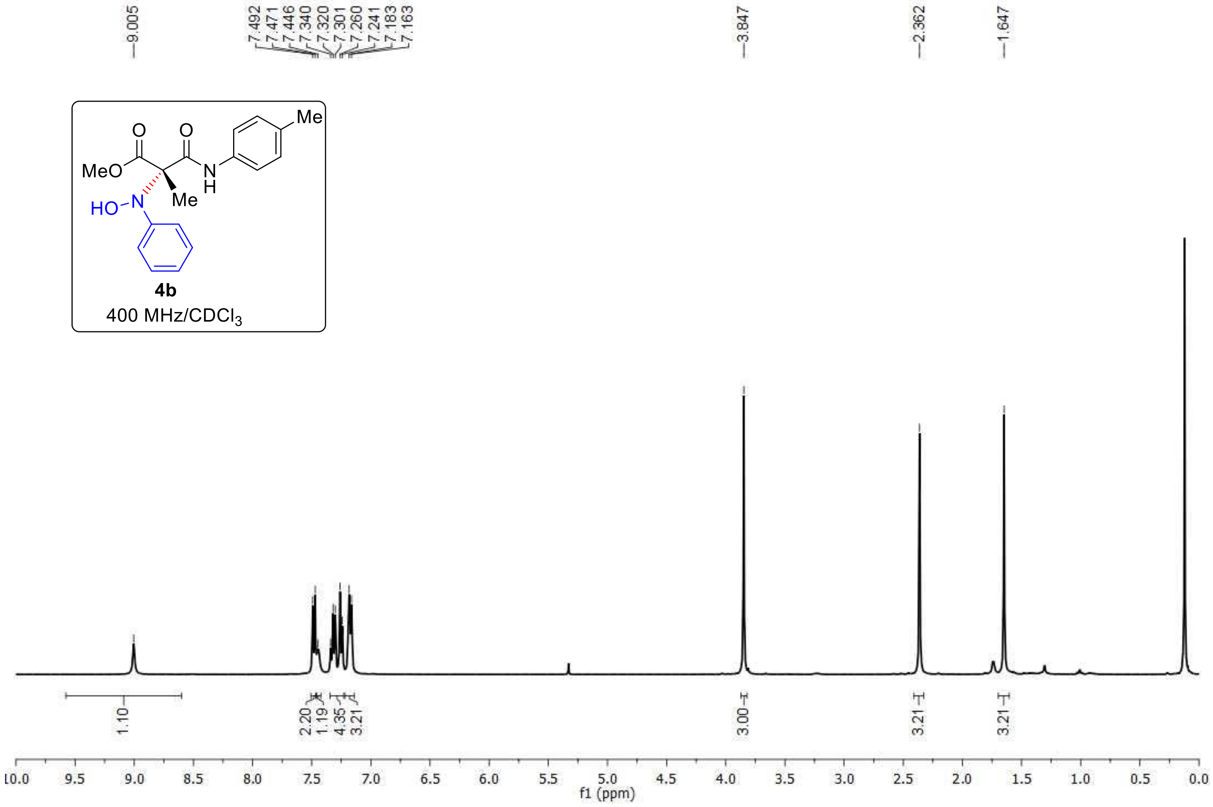
4b
400 MHz/$CDCl_3$
f1 (ppm)

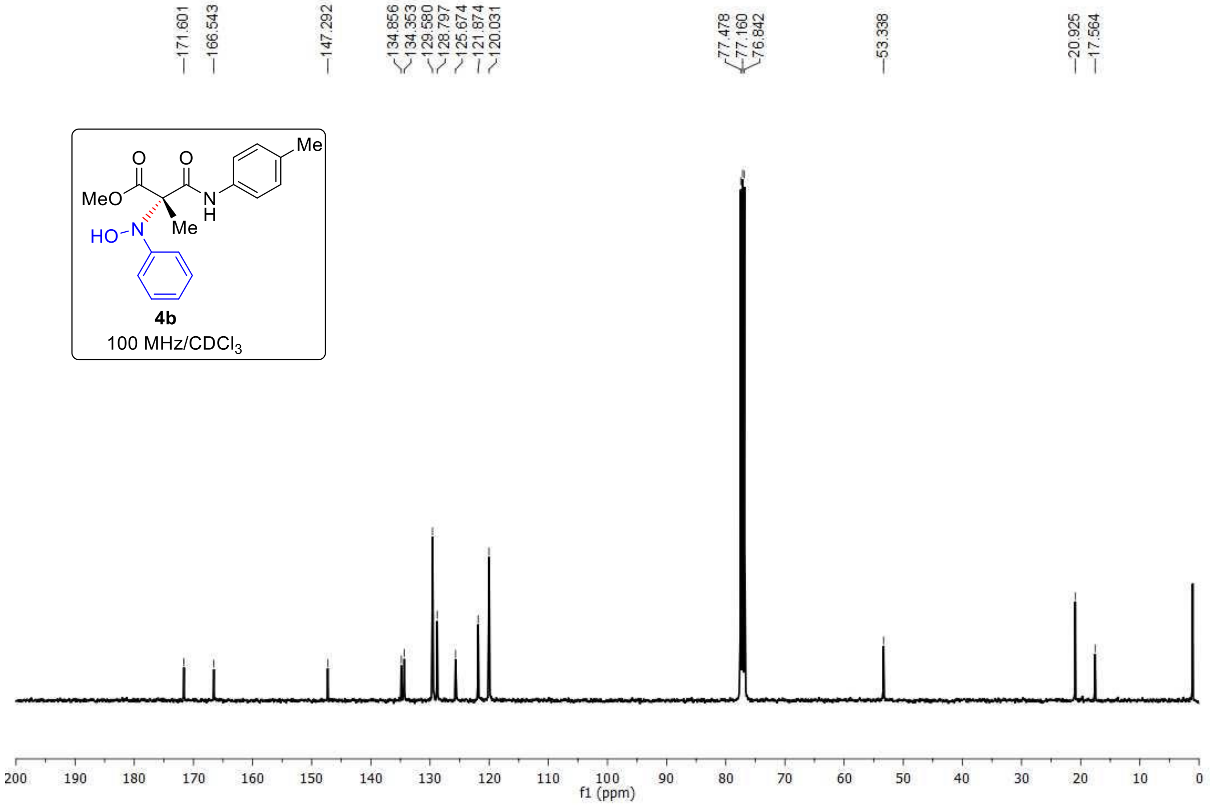
4b
100 MHz/$CDCl_3$
f1 (ppm)

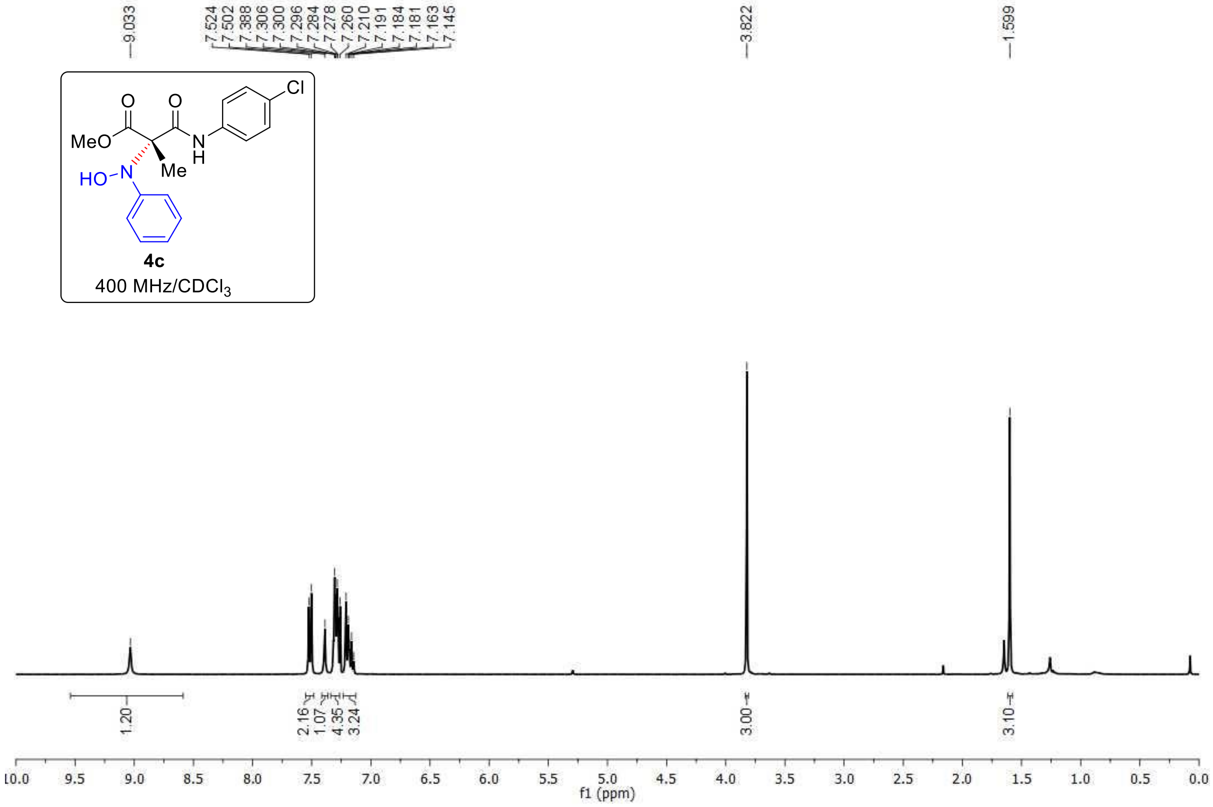

4c
400 MHz/CDCl3

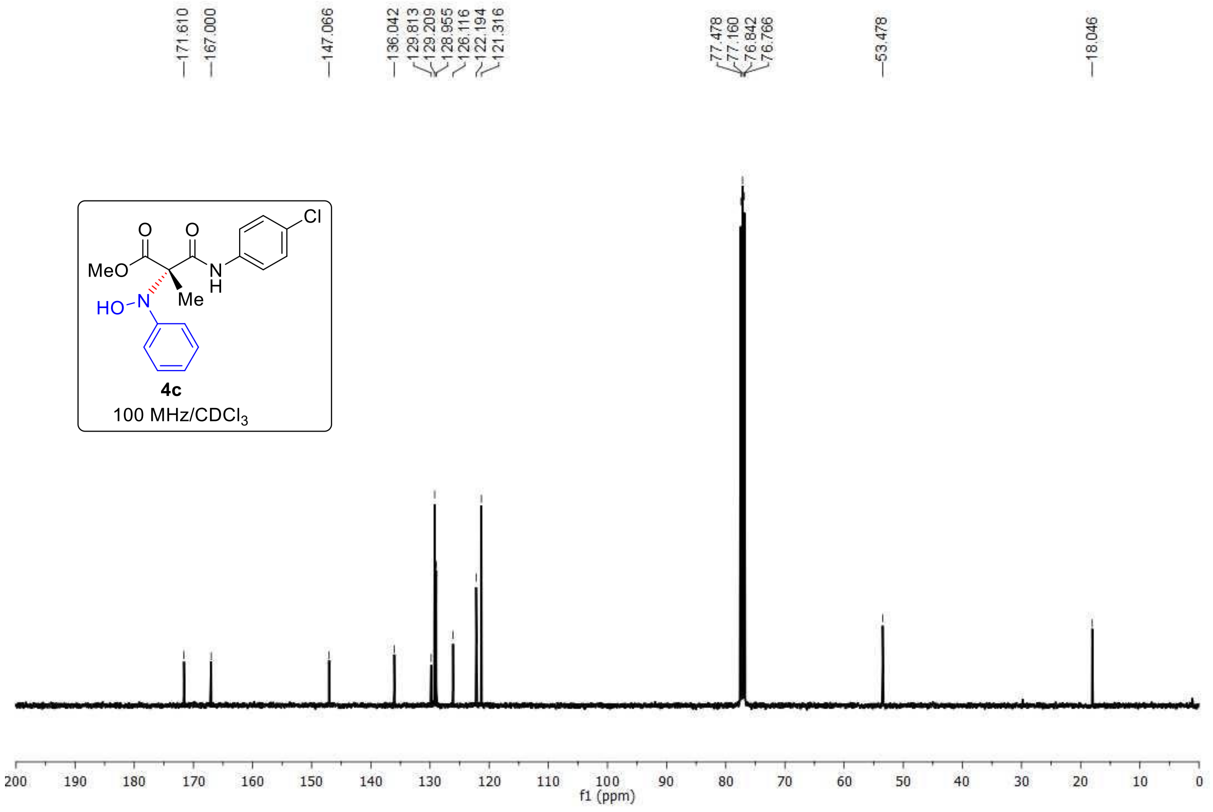

4c
100 MHz/CDCl3

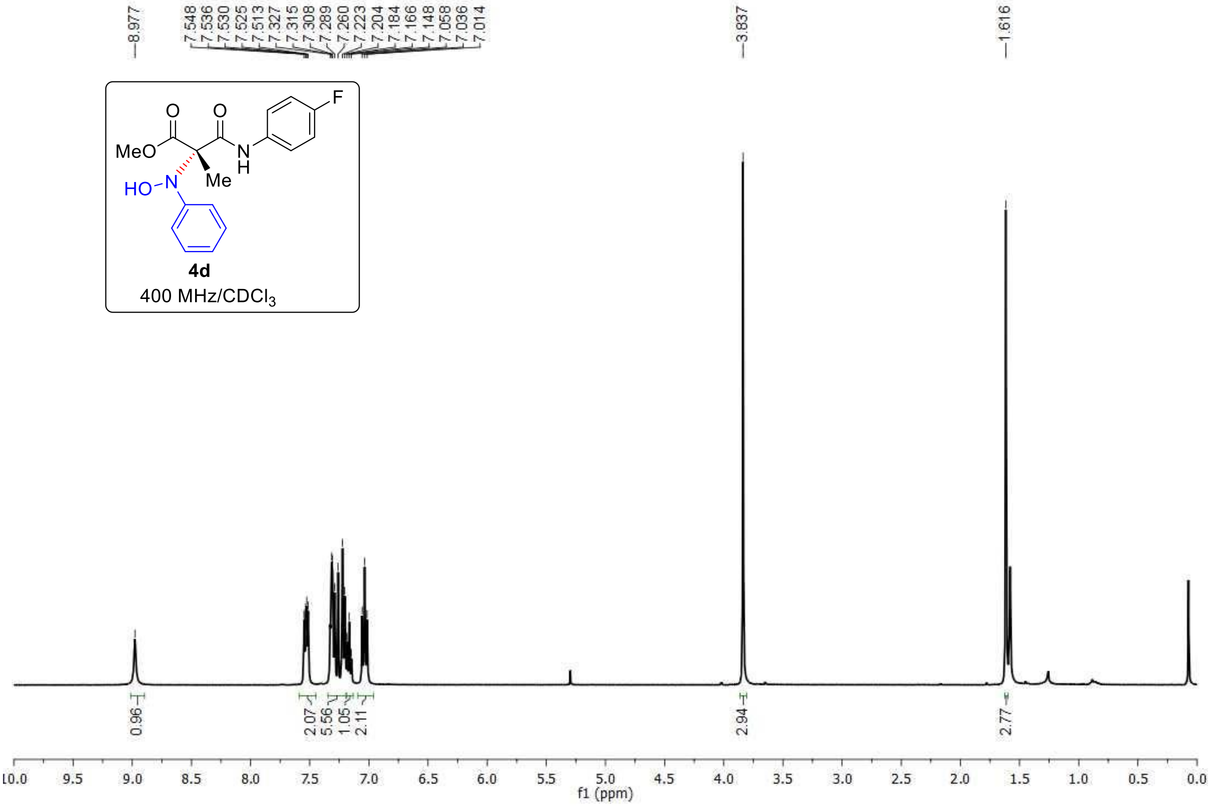
8.977
7.548
7.536
7.530
7.525
7.513
7.327
7.315
7.308
7.289
7.260
7.223
7.204
7.184
7.166
7.148
7.058
7.036
7.014
3.837
1.616
MeO
O
O
N
H
F
Me
HO
N
4d
400 MHz/CDCl3
0.96
2.07
5.56
1.05
2.11
2.94
2.77
f1 (ppm)

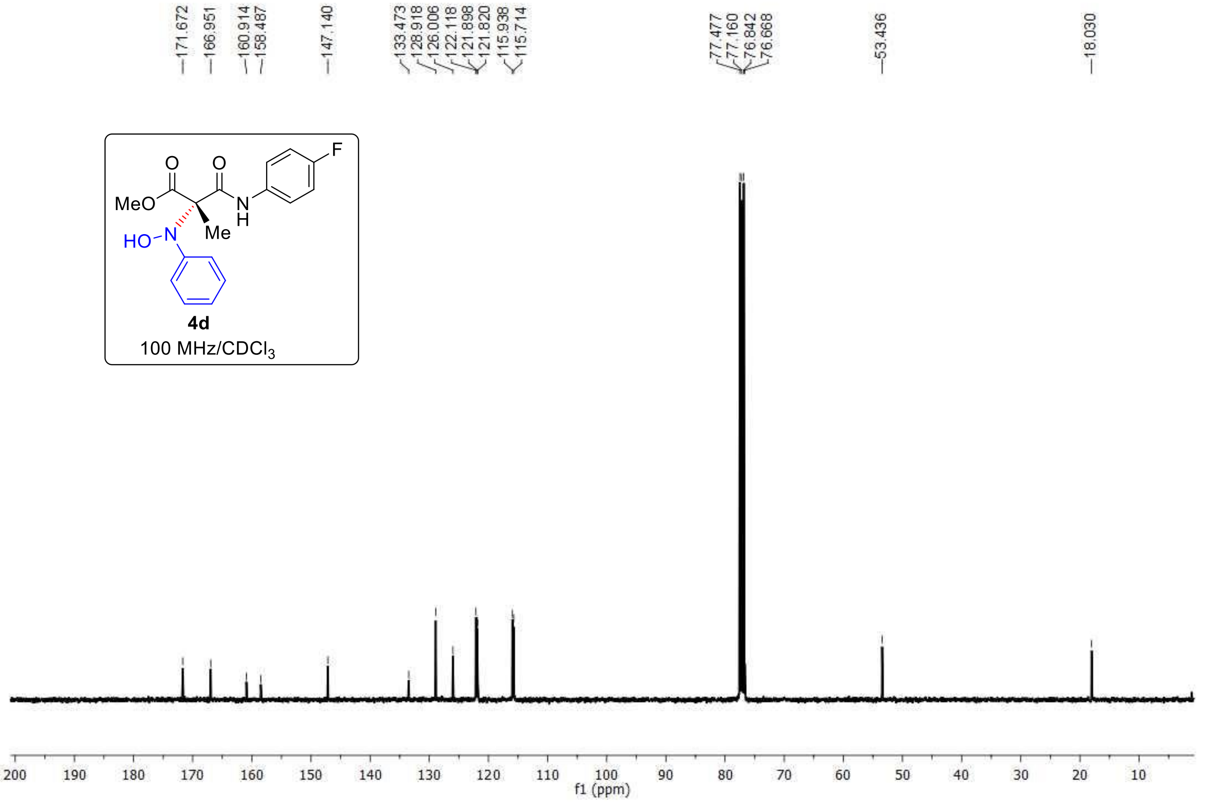
171.672
166.951
160.914
158.487
147.140
133.473
128.918
126.006
122.118
121.898
121.820
115.938
115.714
77.477
77.160
76.842
76.668
53.436
18.030
MeO
O
O
N
H
F
Me
HO
N
4d
100 MHz/CDCl3
f1 (ppm)

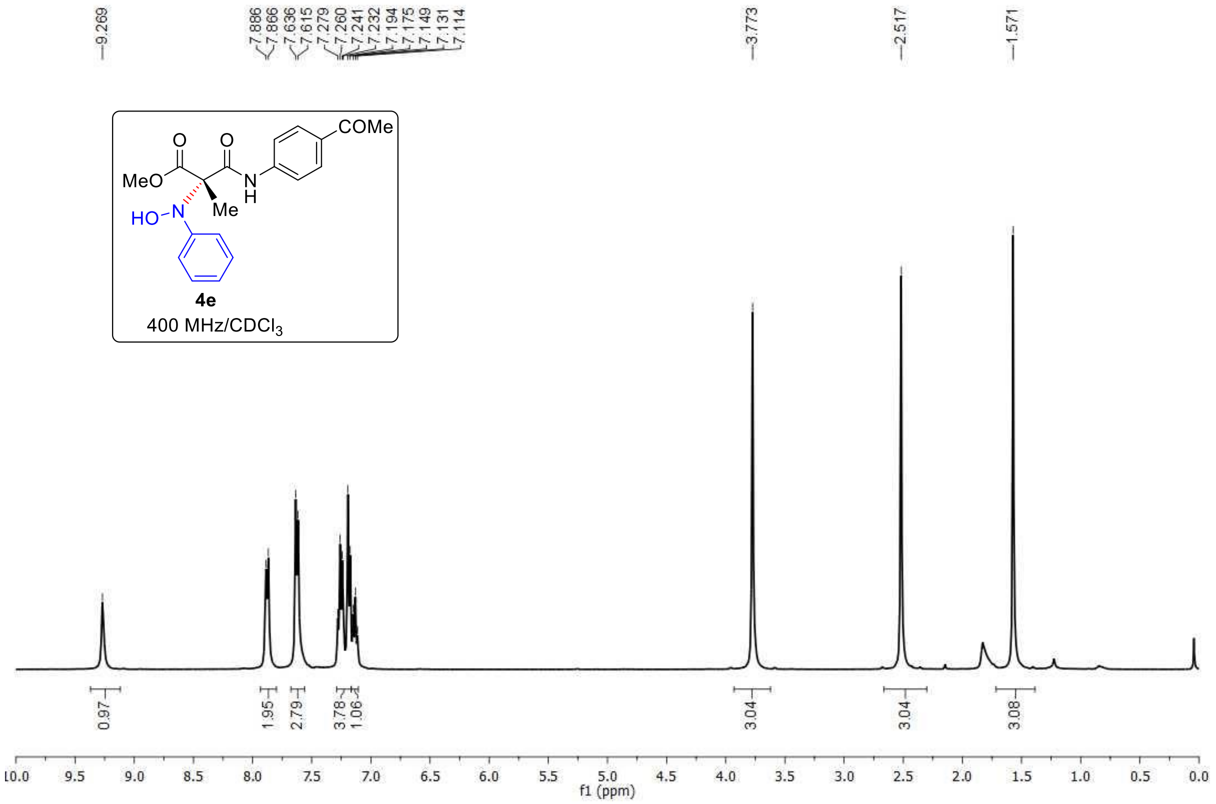
9.269
7.886 7.866 7.636 7.615 7.279 7.260 7.241 7.232 7.194 7.175 7.149 7.131 7.114
3.773
2.517
1.571
COMe
O O
MeO N H
HO N
Me
4e
400 MHz/CDCl3
0.97 1.95 2.79 3.78 1.06 3.04 3.04 3.08
10.0 9.5 9.0 8.5 8.0 7.5 7.0 6.5 6.0 5.5 5.0 4.5 4.0 3.5 3.0 2.5 2.0 1.5 1.0 0.5 0.0
f1 (ppm)

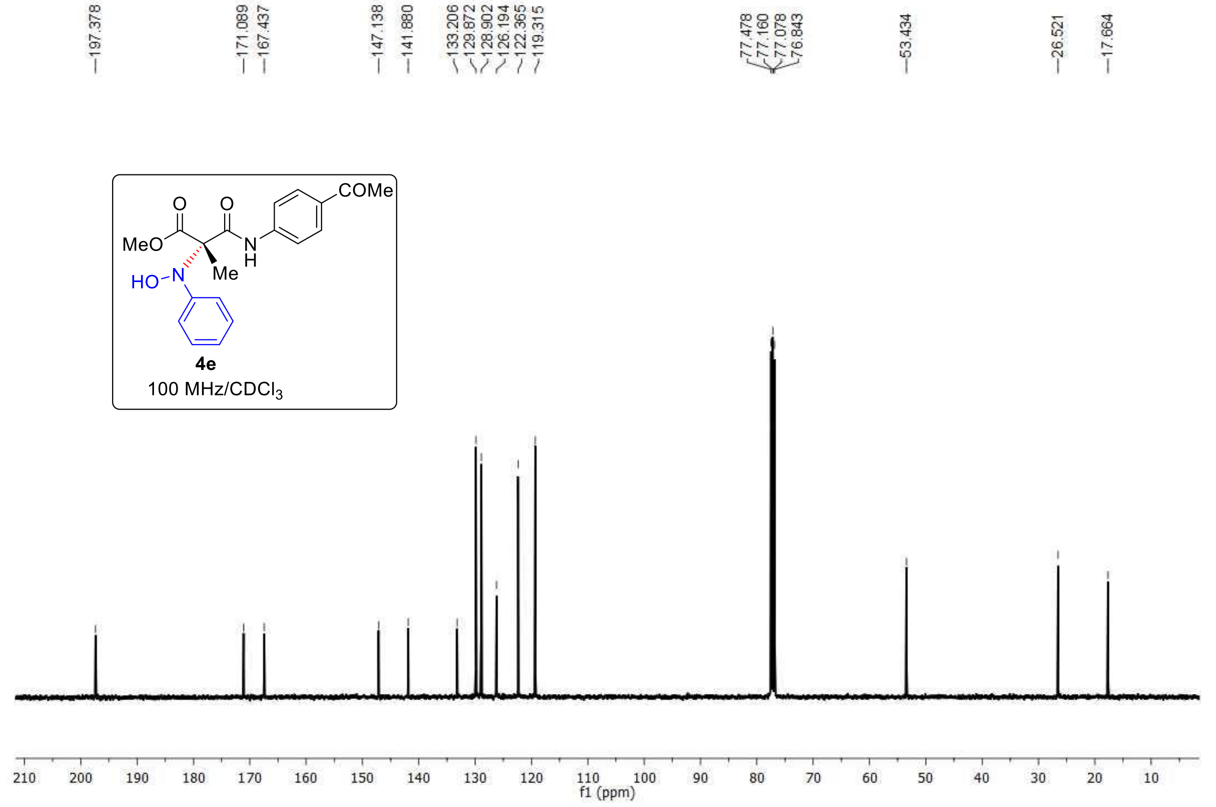
197.378
171.089 167.437
147.138 141.880
133.206 129.872 128.902 126.194 122.365 119.315
77.478 77.160 77.078 76.843
53.434
26.521 17.664
COMe
O O
MeO N H
HO N
Me
4e
100 MHz/CDCl3
210 200 190 180 170 160 150 140 130 120 110 100 90 80 70 60 50 40 30 20 10
f1 (ppm)

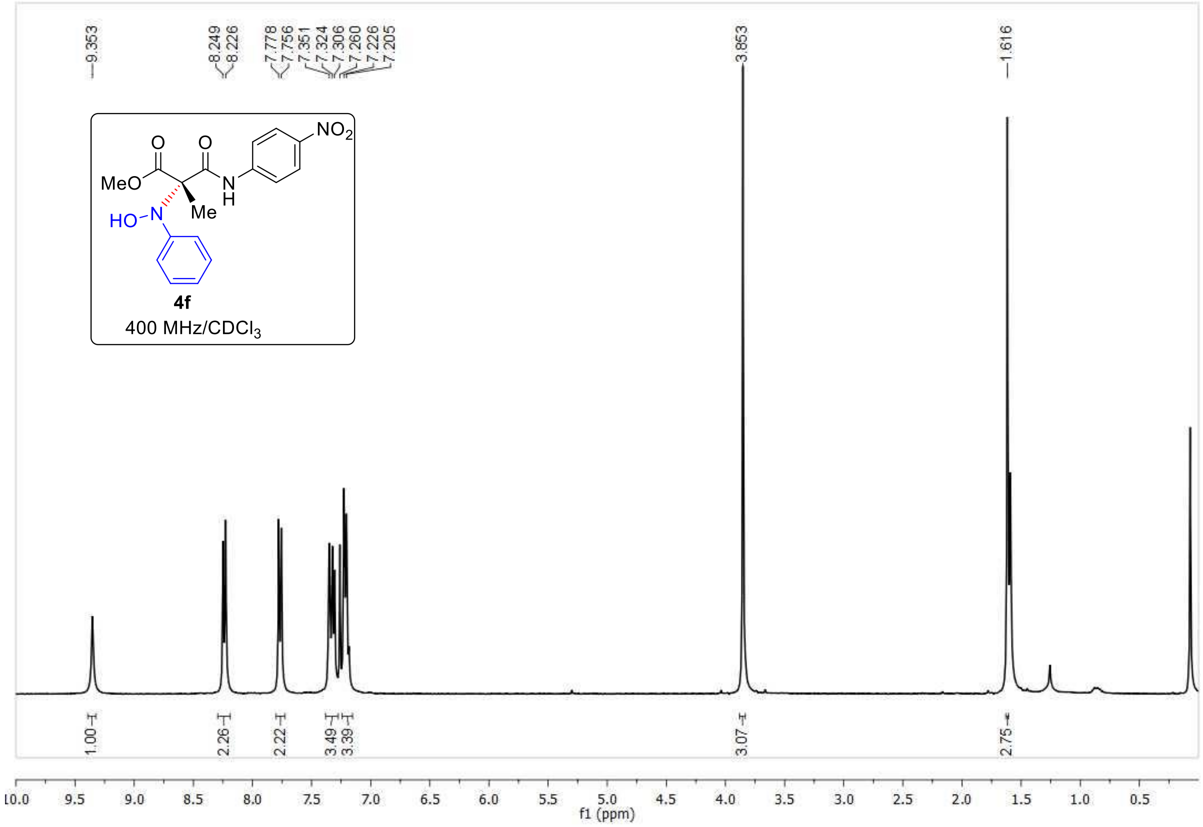
9.353
8.249
8.226
7.778
7.756
7.351
7.324
7.306
7.260
7.226
7.205
3.853
1.616
NO2
MeO
O
O
N
H
HO
N
Me
4f
400 MHz/CDCl3
1.00
2.26
2.22
3.49
3.39
3.07
2.75
f1 (ppm)

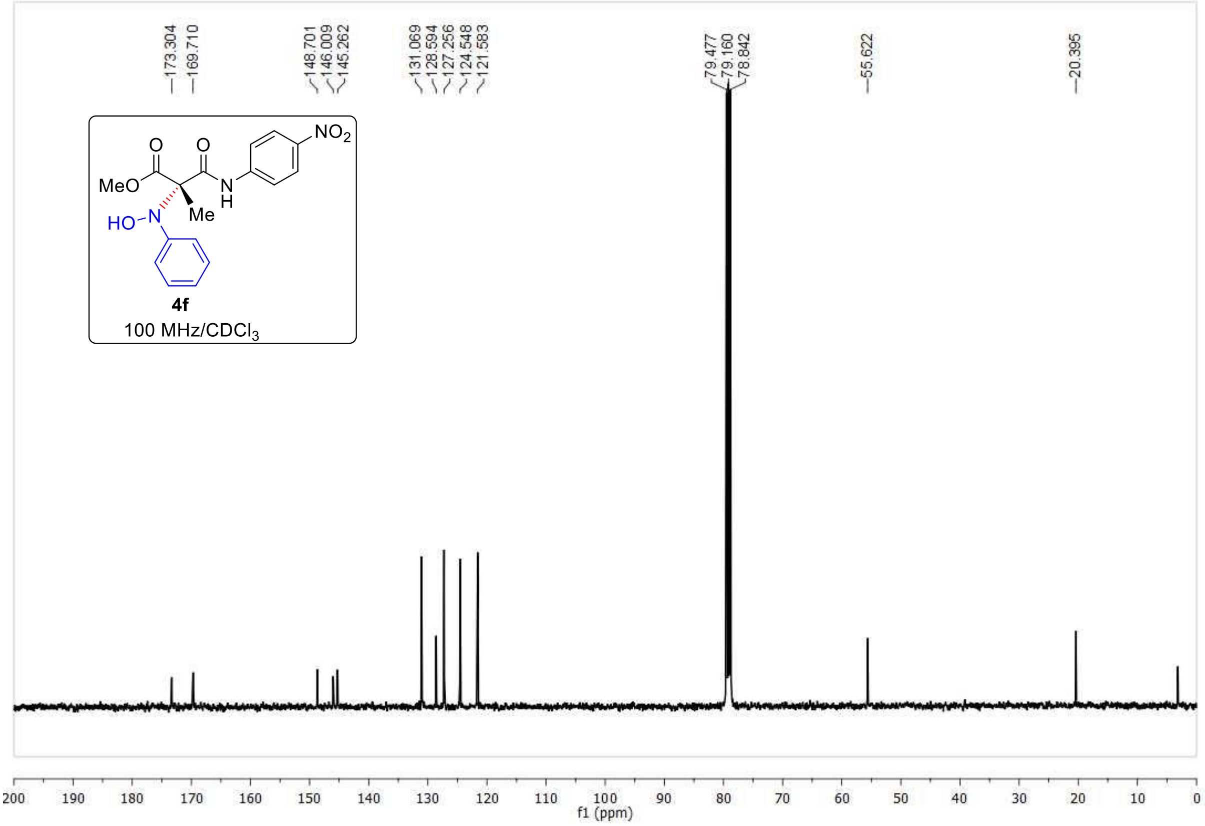
173.304
169.710
148.701
146.009
145.262
131.069
128.594
127.256
124.548
121.583
79.477
79.160
78.842
55.622
20.395
NO2
MeO
O
O
N
H
HO
N
Me
4f
100 MHz/CDCl3
f1 (ppm)

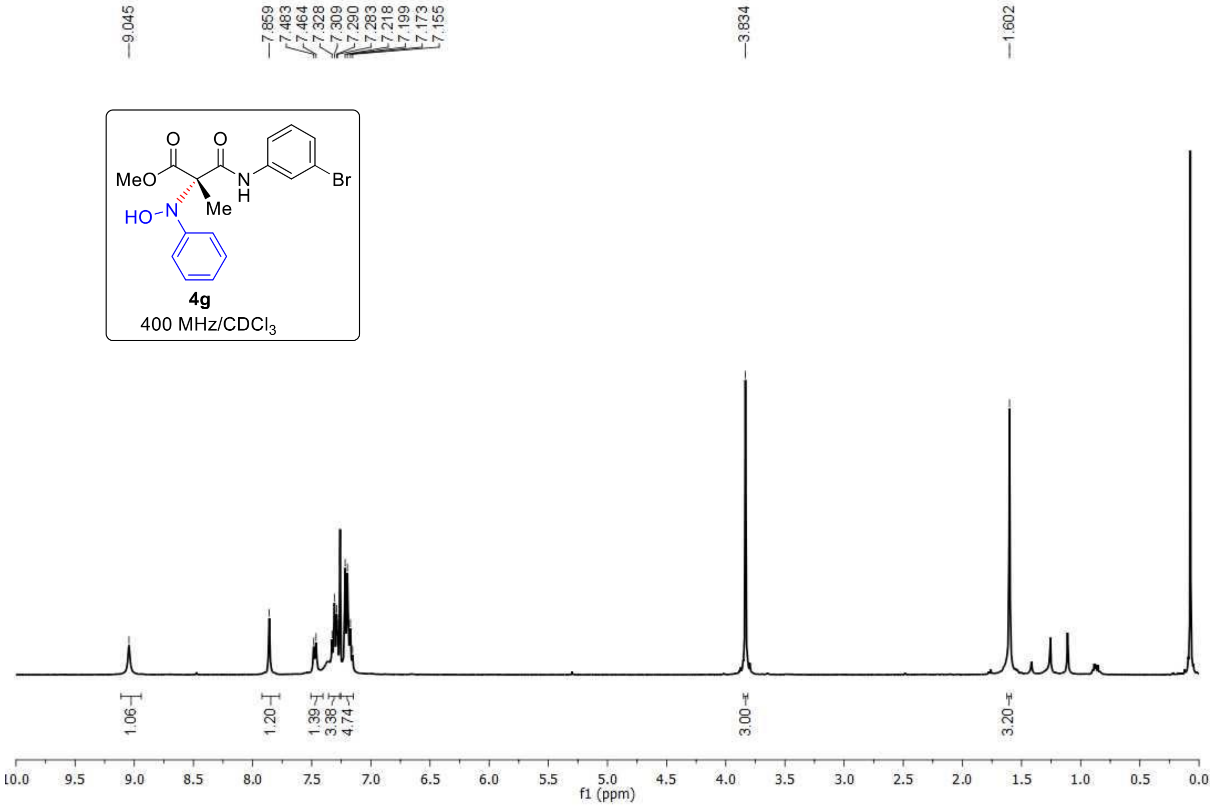
9.045
7.859
7.483
7.464
7.328
7.309
7.290
7.283
7.218
7.199
7.173
7.155
3.834
1.602
MeO
O
O
N
H
Br
Me
HO
N
4g
400 MHz/CDCl3
1.06
1.20
1.39
3.38
4.74
3.00
3.20
f1 (ppm)

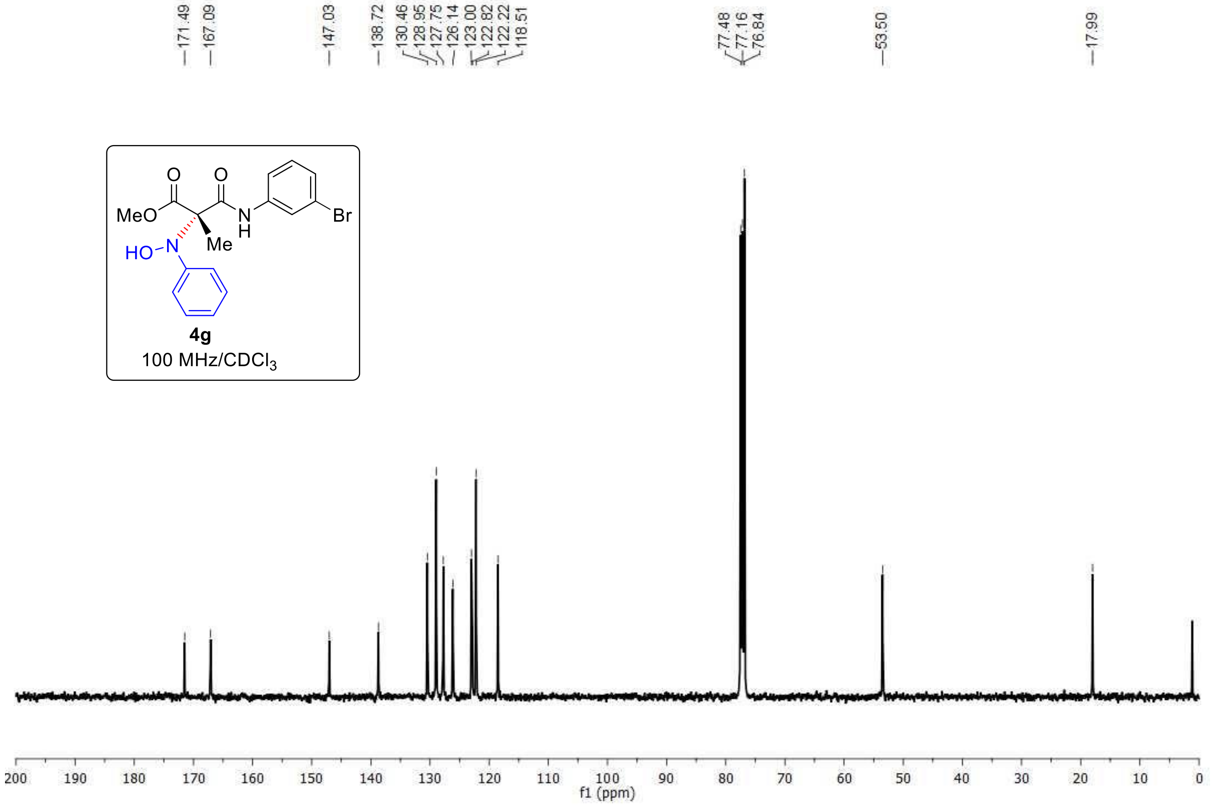
171.49
167.09
147.03
138.72
130.46
128.95
127.75
126.14
123.00
122.82
122.22
118.51
77.48
77.16
76.84
53.50
17.99
MeO
O
O
N
H
Br
Me
HO
N
4g
100 MHz/CDCl3
f1 (ppm)

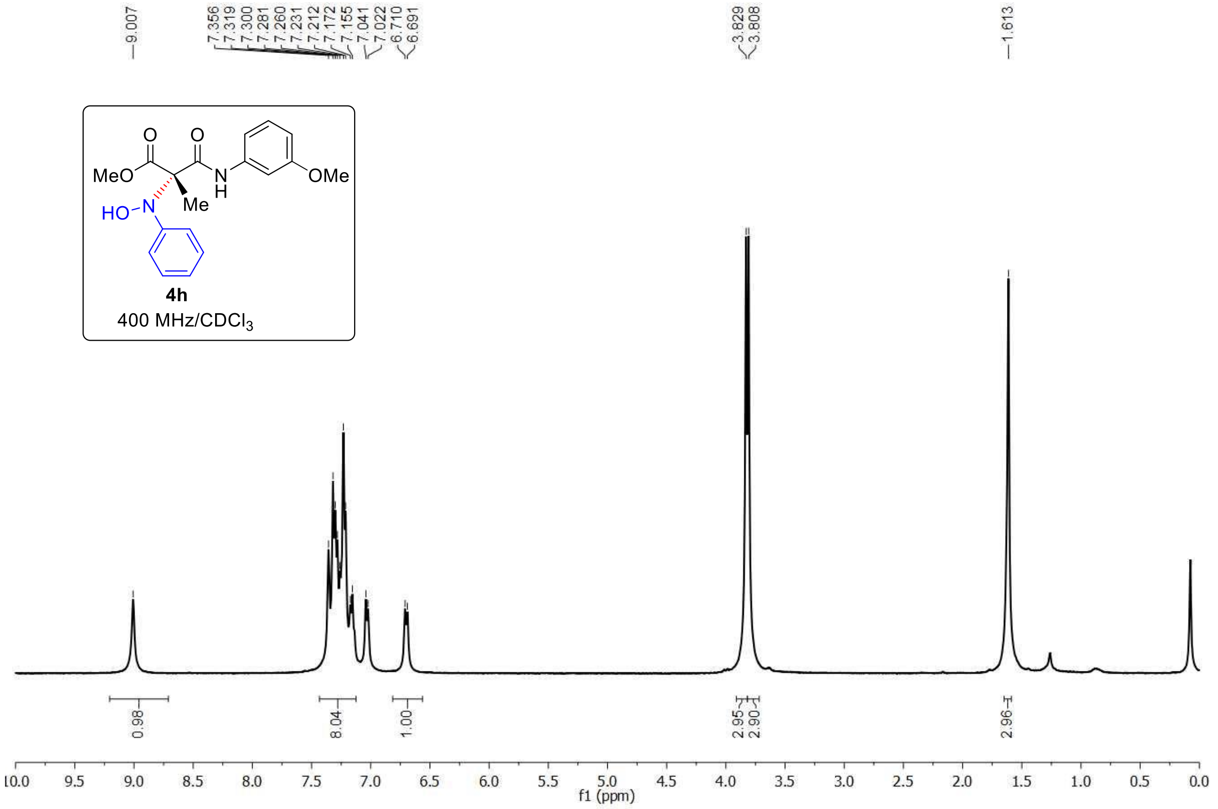
9.007
7.356
7.319
7.300
7.281
7.260
7.231
7.212
7.172
7.155
7.041
7.022
6.710
6.691
3.829
3.808
1.613
MeO
HO
Me
OMe
4h
400 MHz/CDCl3
0.98
8.04
1.00
2.95
2.90
2.96
f1 (ppm)

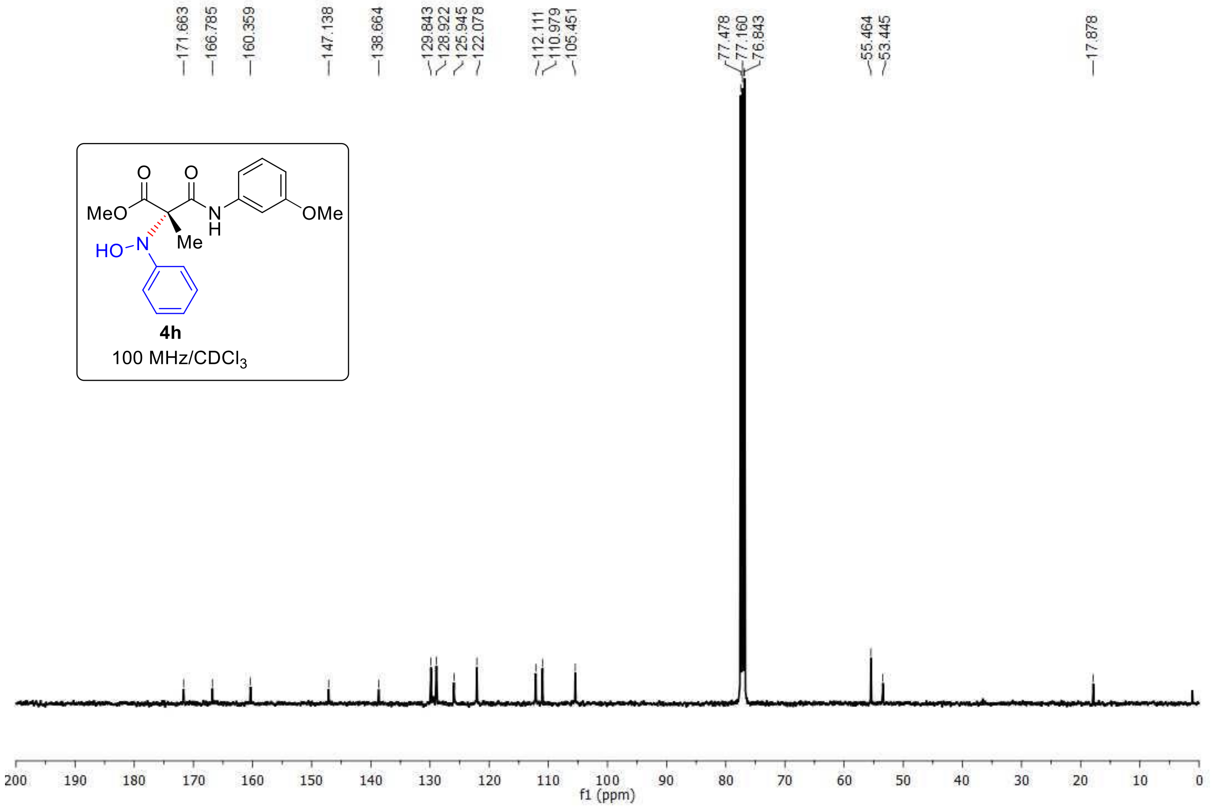
171.663
166.785
160.359
147.138
138.664
129.843
128.922
125.945
122.078
112.111
110.979
105.451
77.478
77.160
76.843
55.464
53.445
17.878
MeO
HO
Me
OMe
4h
100 MHz/CDCl3
f1 (ppm)

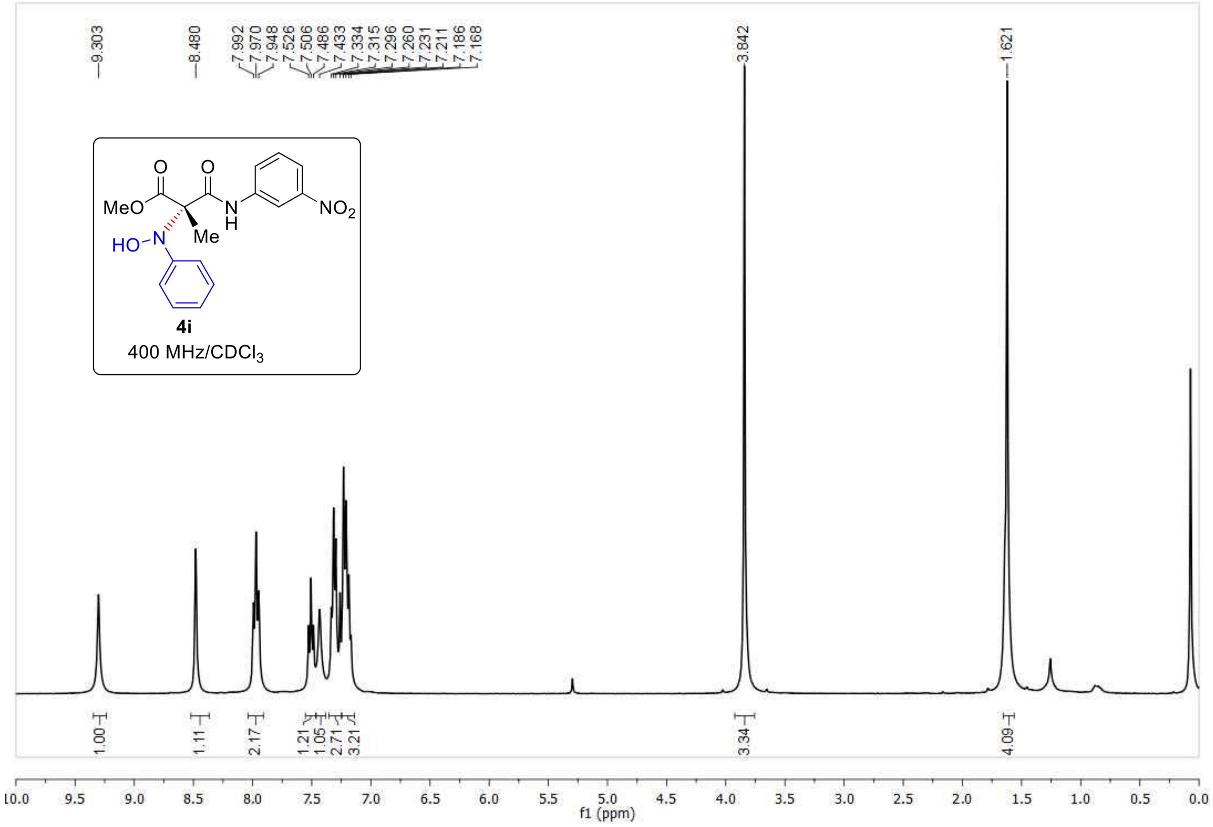
9.303
8.480
7.992
7.970
7.948
7.526
7.506
7.486
7.433
7.334
7.315
7.296
7.260
7.231
7.211
7.186
7.168
3.842
1.621
MeO
O
O
N
H
NO2
HO
N
Me
4i
400 MHz/CDCl3
1.00
1.11
2.17
1.21
1.05
2.71
3.21
3.34
4.09
f1 (ppm)

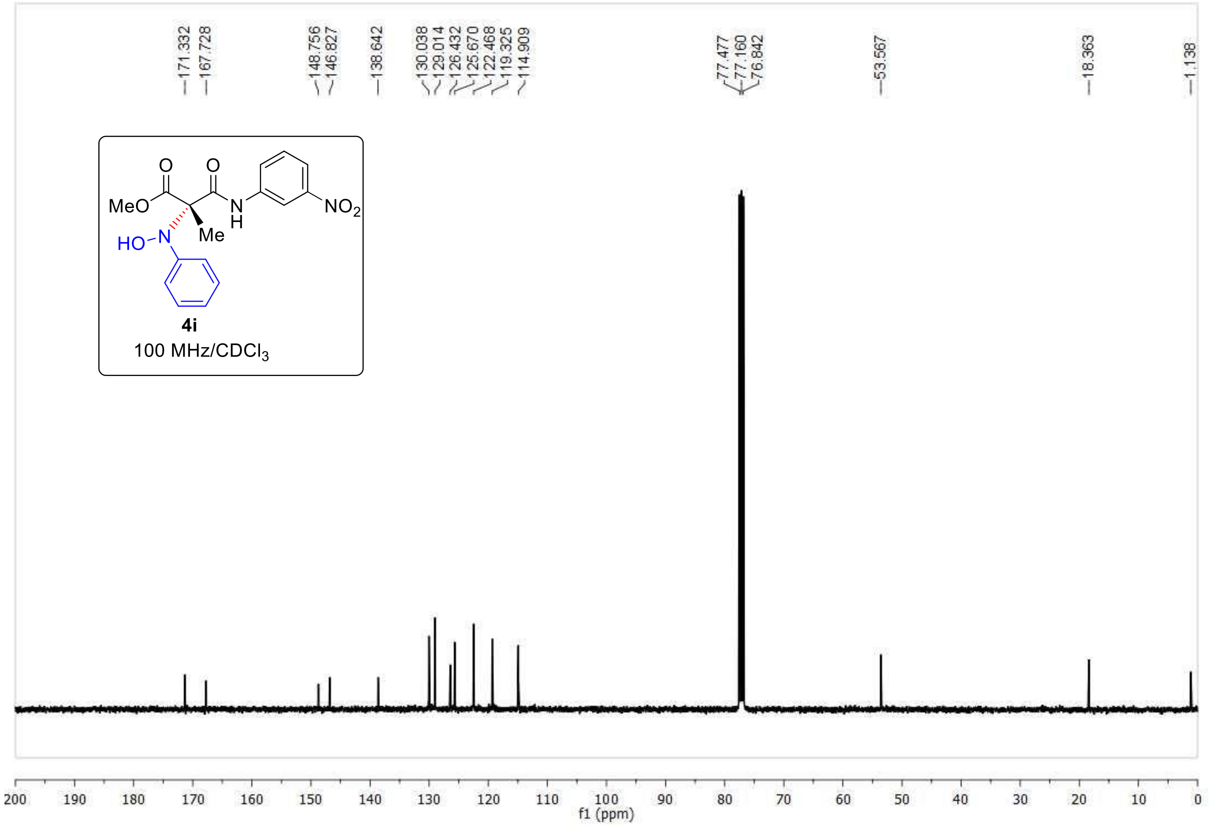
171.332
167.728
148.756
146.827
138.642
130.038
129.014
126.432
125.670
122.468
119.325
114.909
77.477
77.160
76.842
53.567
18.363
1.138
MeO
O
O
N
H
NO2
HO
N
Me
4i
100 MHz/CDCl3
f1 (ppm)

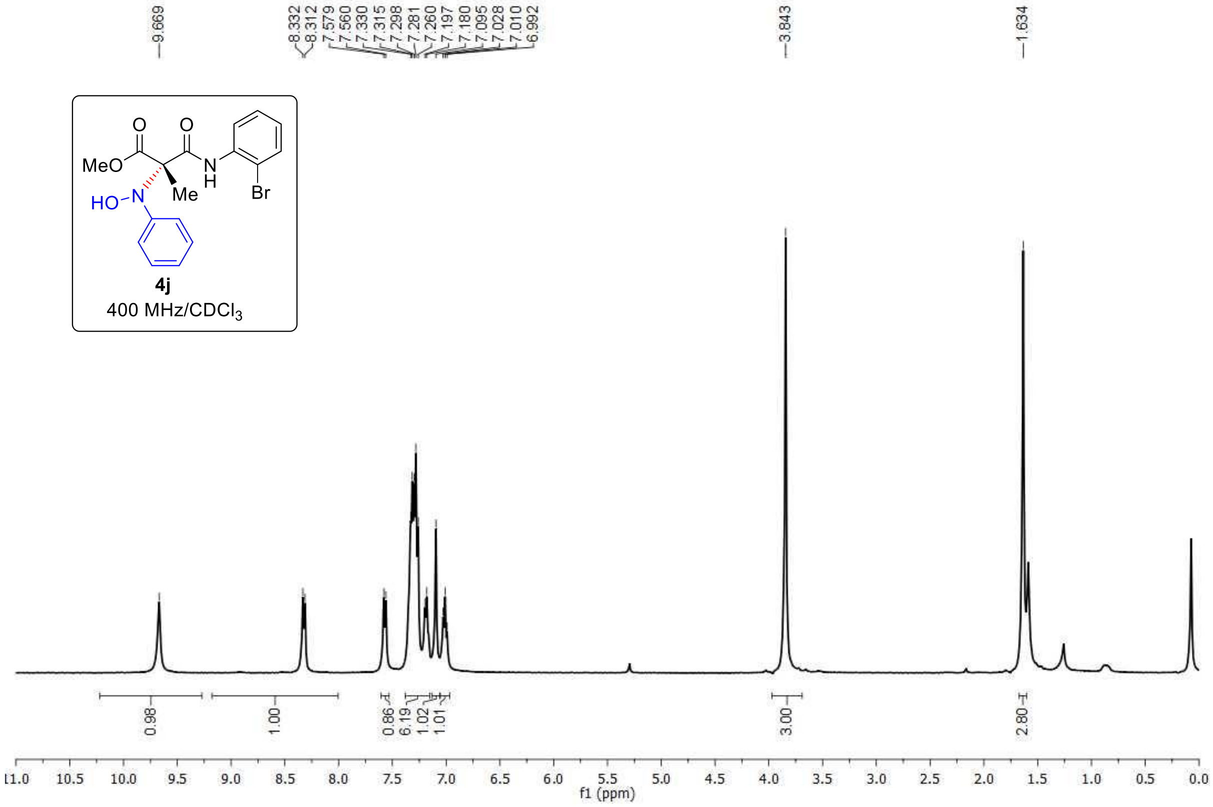
9.669
8.332
8.312
7.579
7.560
7.330
7.315
7.298
7.281
7.260
7.197
7.180
7.095
7.028
7.010
6.992
3.843
1.634
MeO
O
O
N
H
Br
Me
HO
N
4j
400 MHz/CDCl3
0.98
1.00
0.86
6.19
1.02
1.01
3.00
2.80
f1 (ppm)

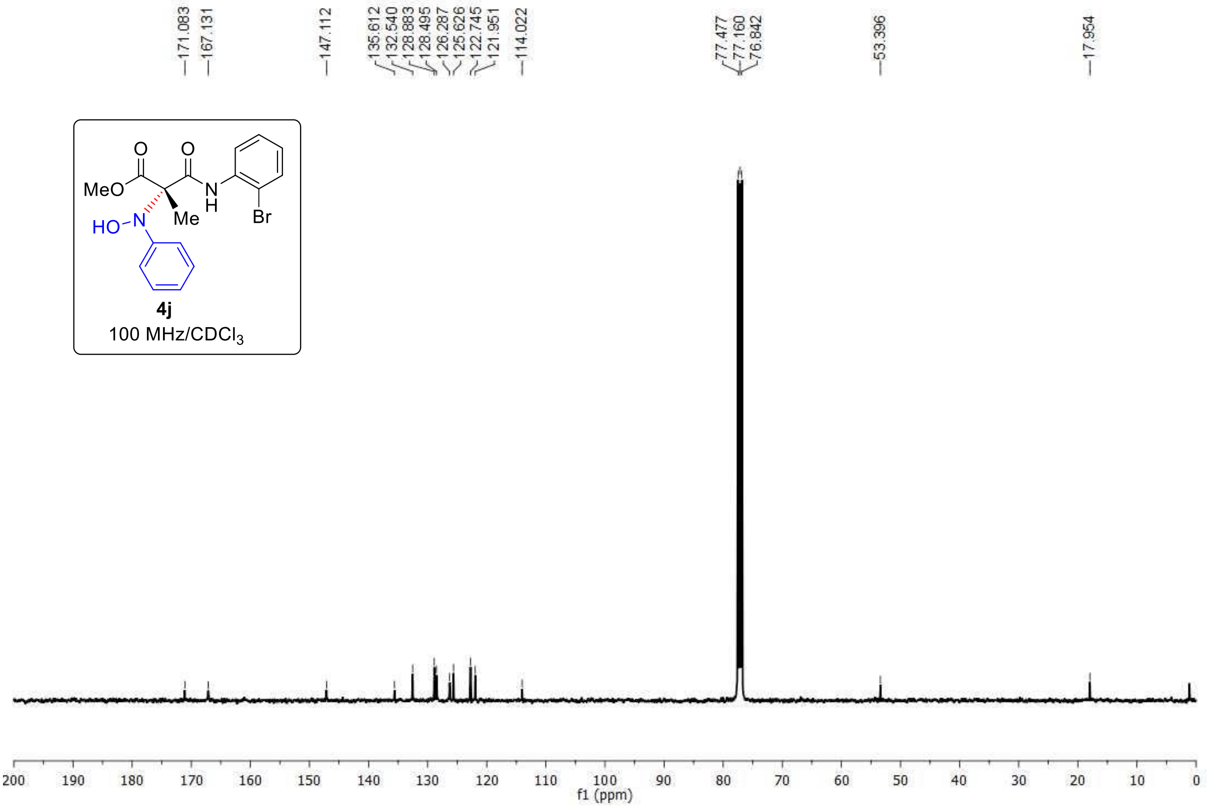
171.083
167.131
147.112
135.612
132.540
128.883
128.495
126.287
125.626
122.745
121.951
114.022
77.477
77.160
76.842
53.396
17.954
MeO
O
O
N
H
Br
Me
HO
N
4j
100 MHz/CDCl3
f1 (ppm)

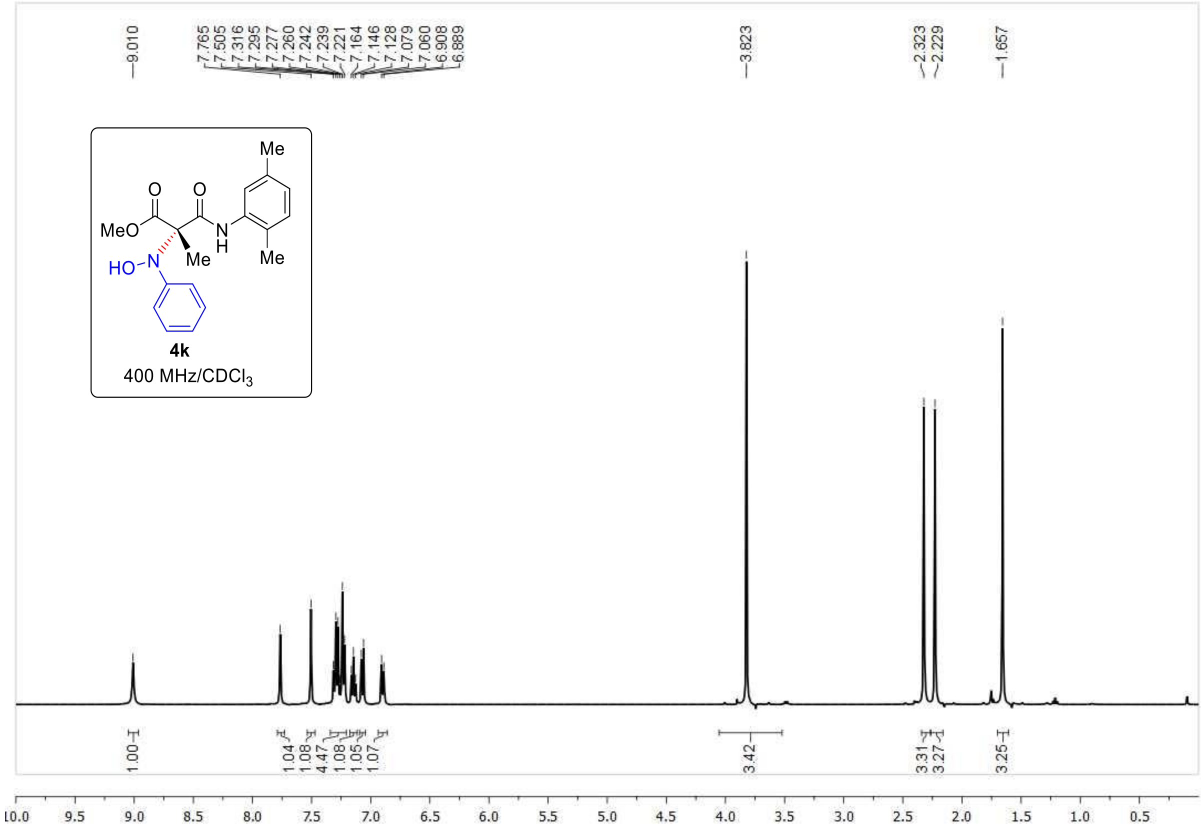
9.010
7.765
7.505
7.316
7.295
7.277
7.260
7.242
7.239
7.221
7.164
7.146
7.128
7.079
7.060
6.908
6.889
3.823
2.323
2.229
1.657
Me
O
O
MeO
N
H
HO
N
Me
Me
4k
400 MHz/CDCl3
1.00
1.04
1.08
4.47
1.08
1.05
1.07
3.42
3.31
3.27
3.25

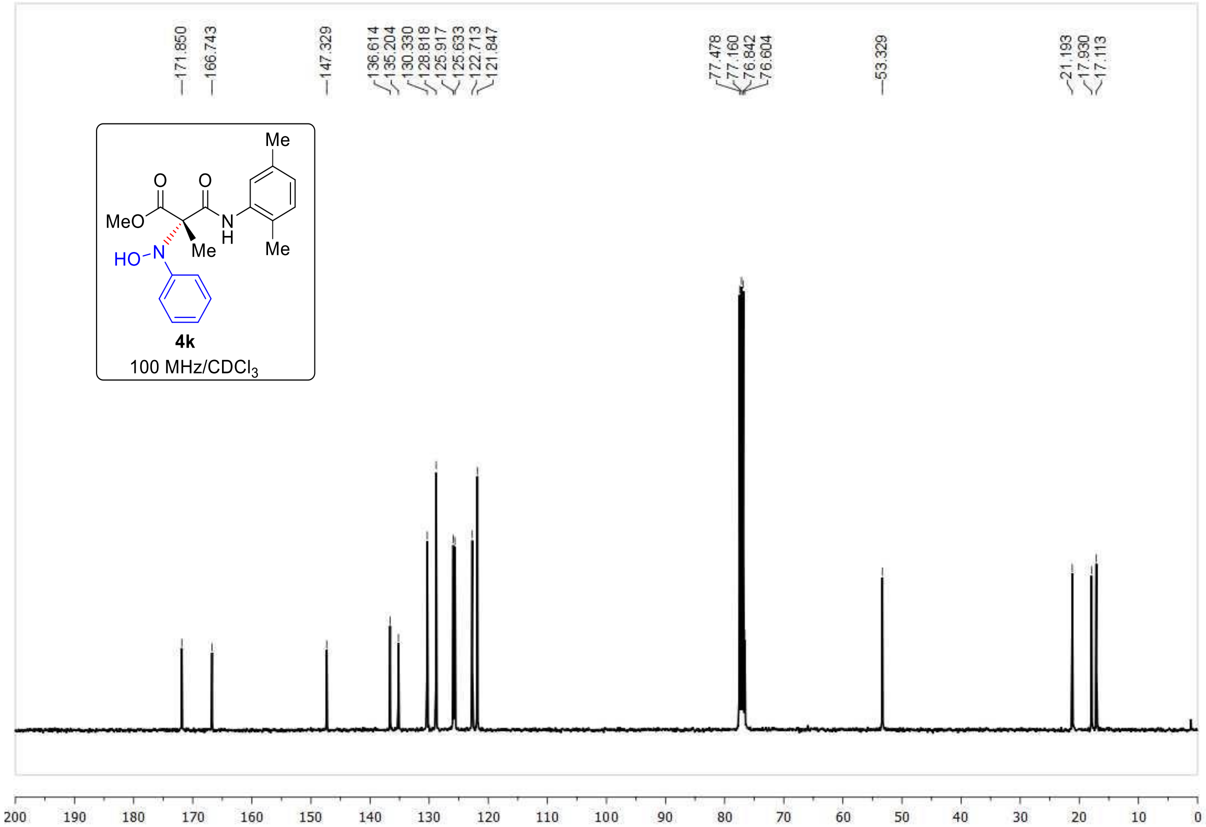
171.850
166.743
147.329
136.614
135.204
130.330
128.818
125.917
125.633
122.713
121.847
77.478
77.160
76.842
76.604
53.329
21.193
17.930
17.113
Me
O
O
MeO
N
H
HO
N
Me
Me
4k
100 MHz/CDCl3

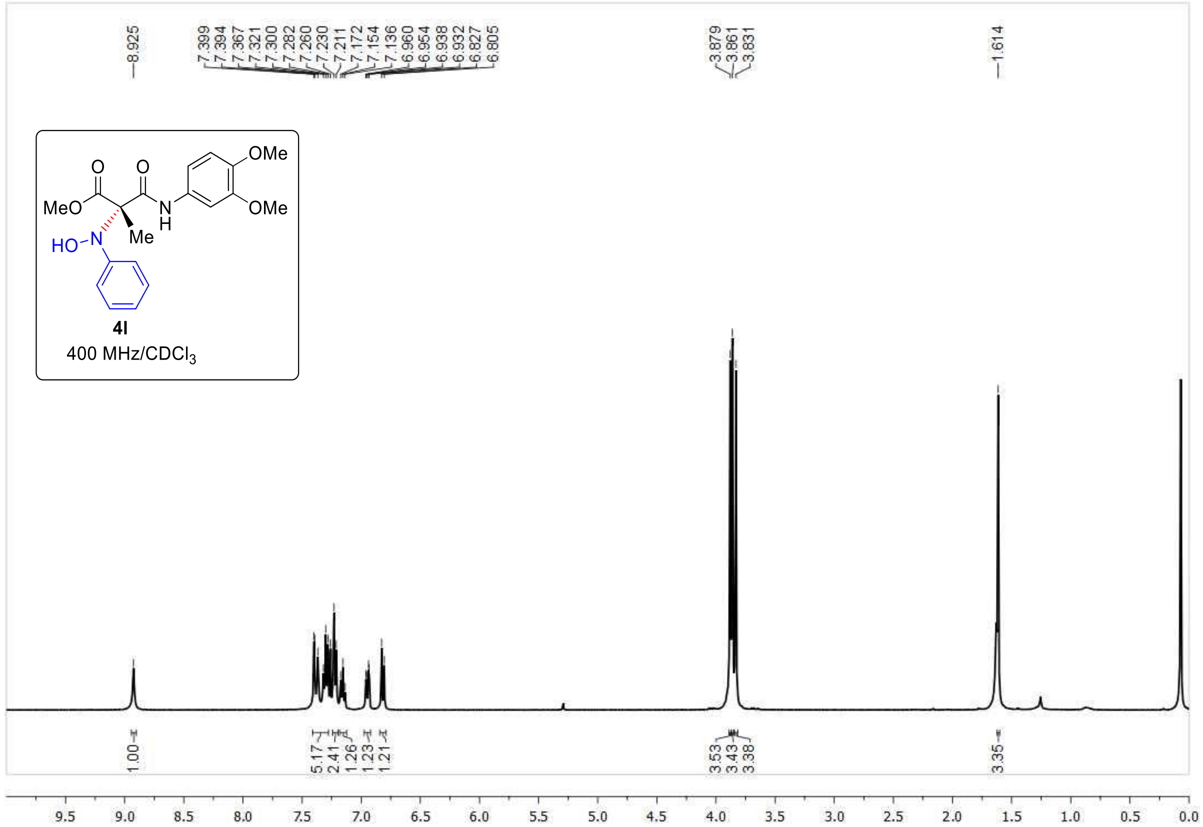

8.925
7.399
7.394
7.367
7.321
7.300
7.282
7.260
7.230
7.211
7.172
7.154
7.136
6.960
6.954
6.938
6.932
6.827
6.805
3.879
3.861
3.831
1.614
4l
400 MHz/CDCl3
1.00
5.17
2.41
1.26
1.23
1.21
3.53
3.43
3.38
3.35

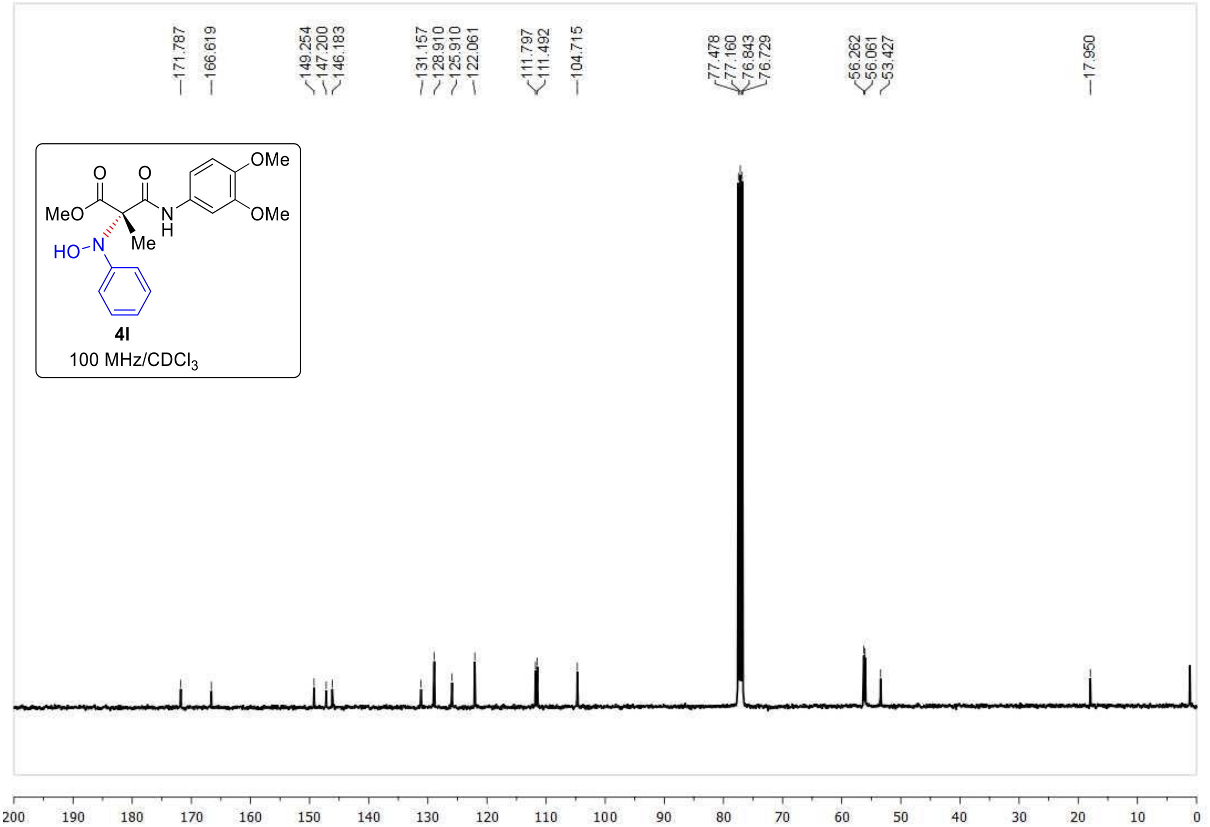

171.787
166.619
149.254
147.200
146.183
131.157
128.910
125.910
122.061
111.797
111.492
104.715
77.478
77.160
76.843
76.729
56.262
56.061
53.427
17.950
4l
100 MHz/CDCl3

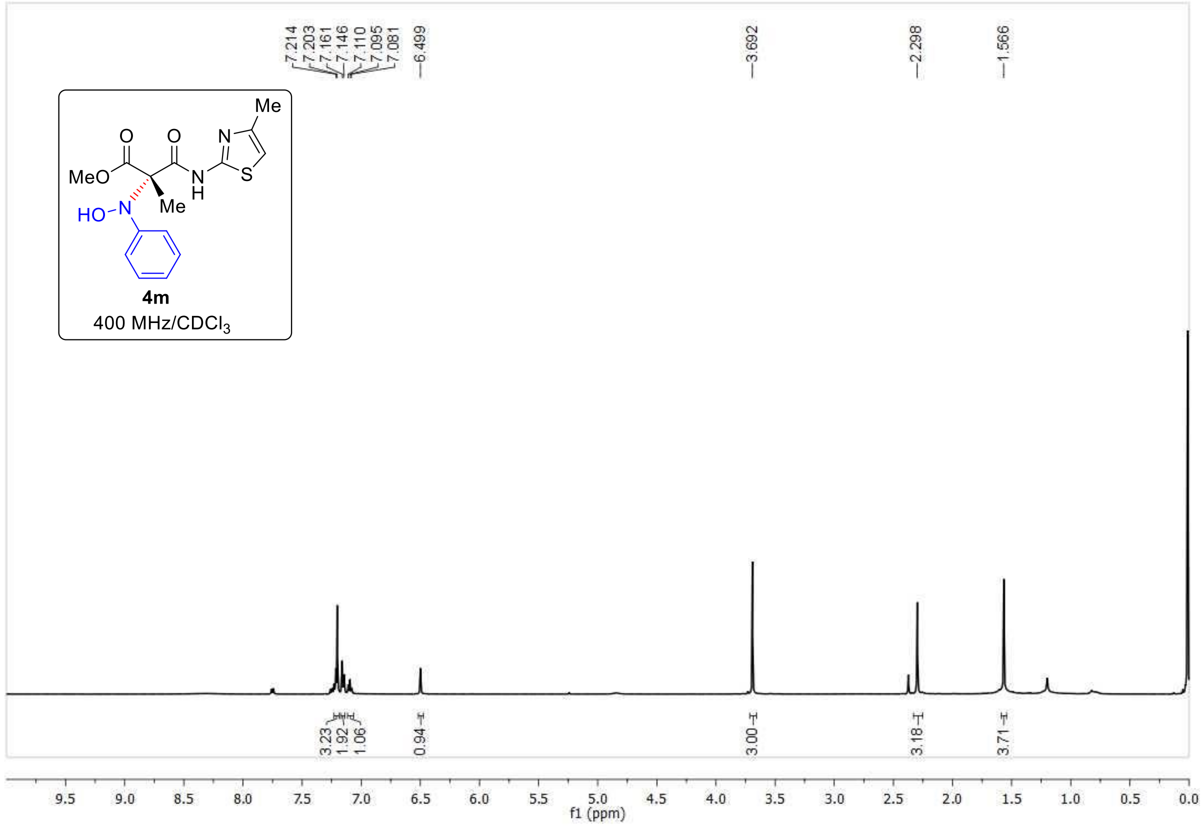
7.214
7.203
7.161
7.146
7.110
7.095
7.081
6.499
3.692
2.298
1.566
Me
O
O
N
MeO
N
H
S
HO
N
Me
4m
400 MHz/CDCl3
3.23
1.92
1.06
0.94
3.00
3.18
3.71
9.5 9.0 8.5 8.0 7.5 7.0 6.5 6.0 5.5 5.0 4.5 4.0 3.5 3.0 2.5 2.0 1.5 1.0 0.5 0.0
f1 (ppm)

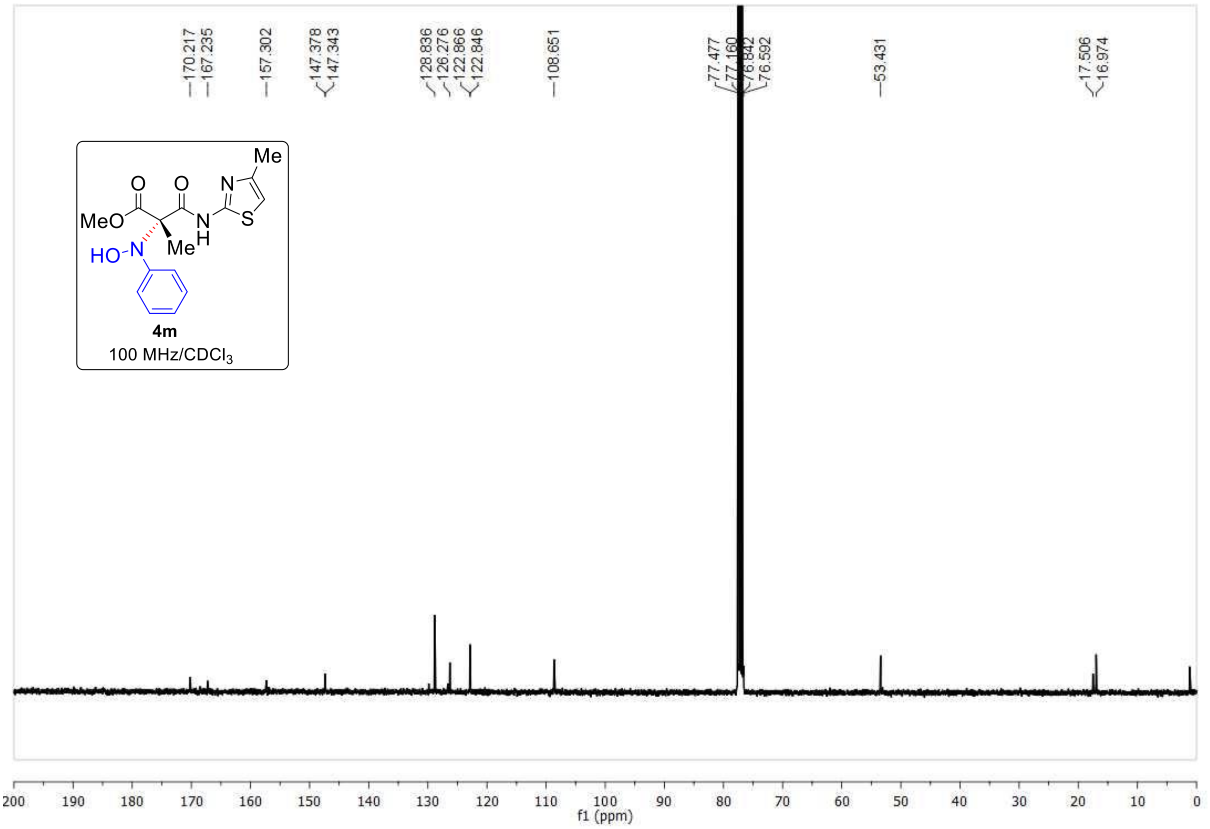
170.217
167.235
157.302
147.378
147.343
128.836
126.276
122.866
122.846
108.651
77.477
77.160
76.842
76.592
53.431
17.506
16.974
Me
O
O
N
MeO
N
H
S
HO
N
Me
4m
100 MHz/CDCl3
200 190 180 170 160 150 140 130 120 110 100 90 80 70 60 50 40 30 20 10 0
f1 (ppm)

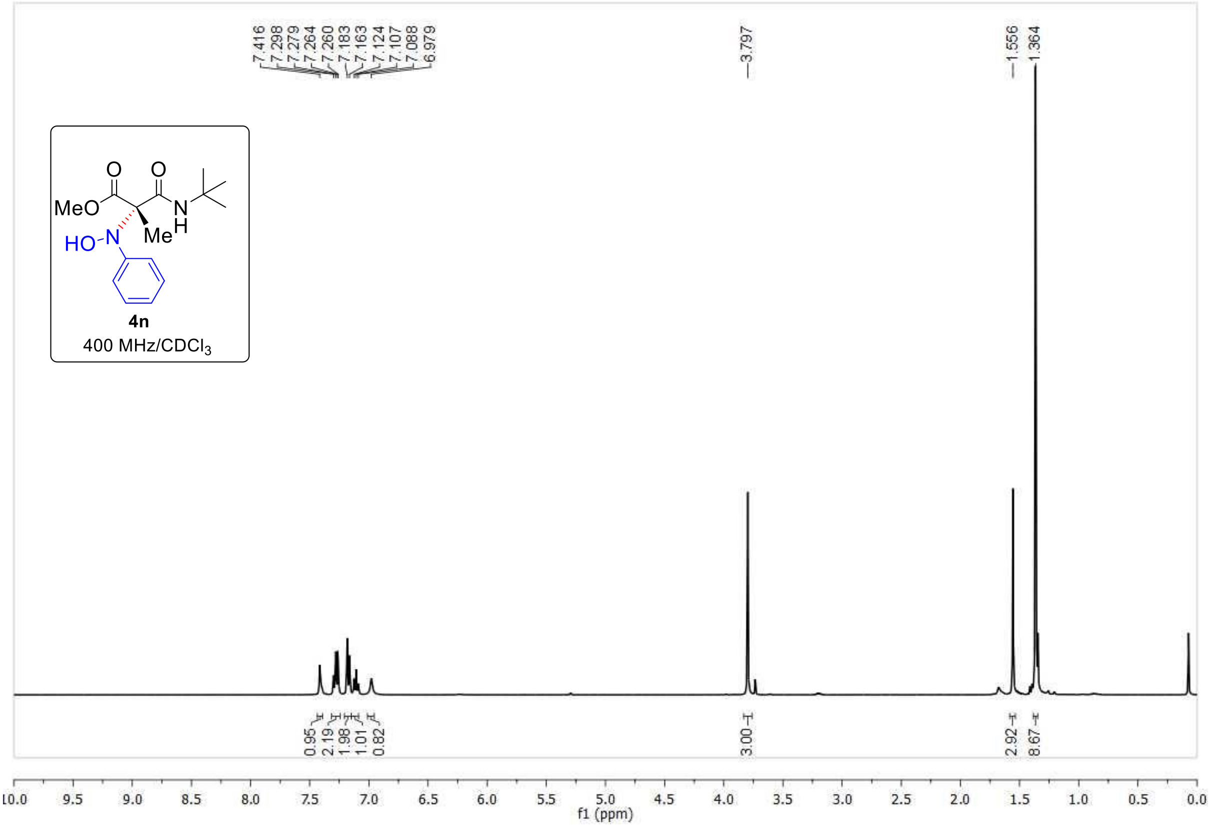

7.416
7.298
7.279
7.264
7.260
7.183
7.163
7.124
7.107
7.088
6.979
3.797
1.556
1.364
MeO
O
O
N
H
Me
HO
N
4n
400 MHz/CDCl3
0.95
2.19
1.98
1.01
0.82
3.00
2.92
8.67
f1 (ppm)

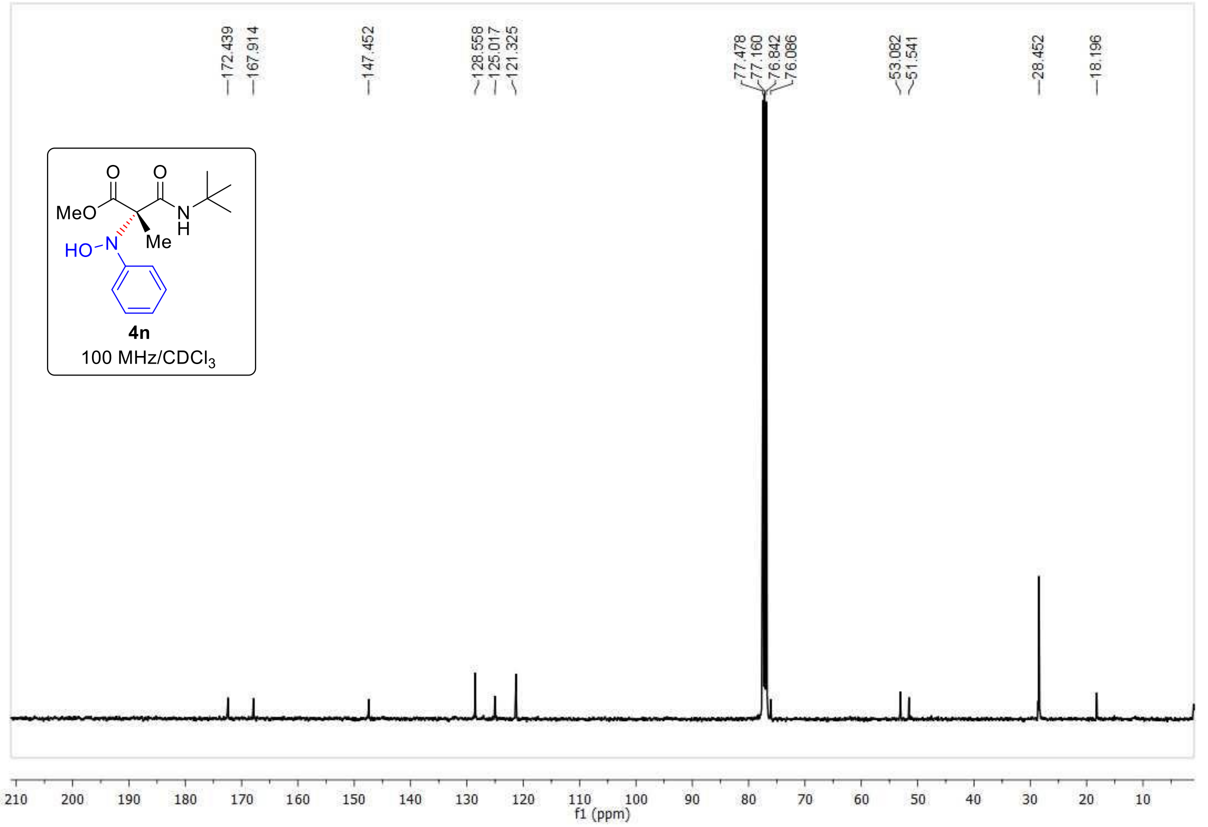

172.439
167.914
147.452
128.558
125.017
121.325
77.478
77.160
76.842
76.086
53.082
51.541
28.452
18.196
MeO
O
O
N
H
Me
HO
N
4n
100 MHz/CDCl3
f1 (ppm)

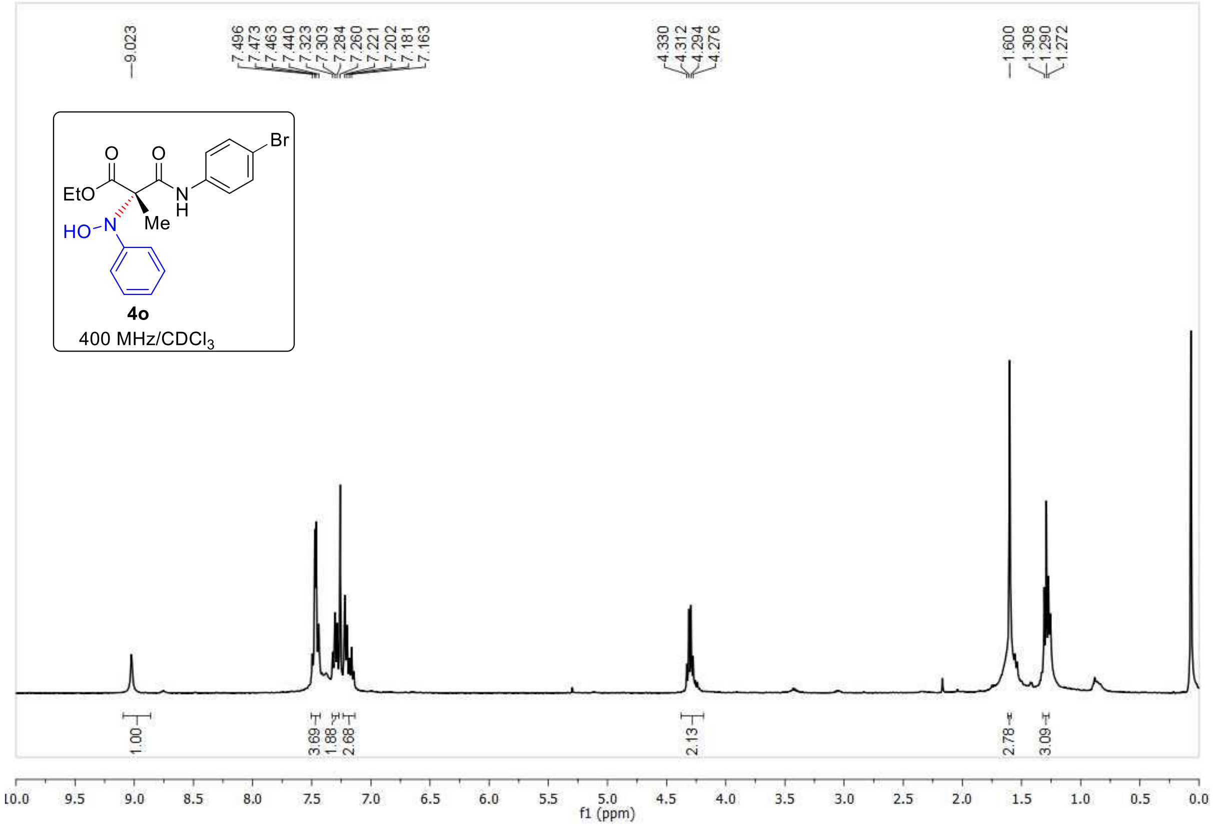
9.023
7.496
7.473
7.463
7.440
7.323
7.303
7.284
7.260
7.221
7.202
7.181
7.163
4.330
4.312
4.294
4.276
1.600
1.308
1.290
1.272
EtO
O
O
N
H
Br
HO
N
Me
4o
400 MHz/CDCl3
1.00
3.69
1.88
2.68
2.13
2.78
3.09
f1 (ppm)

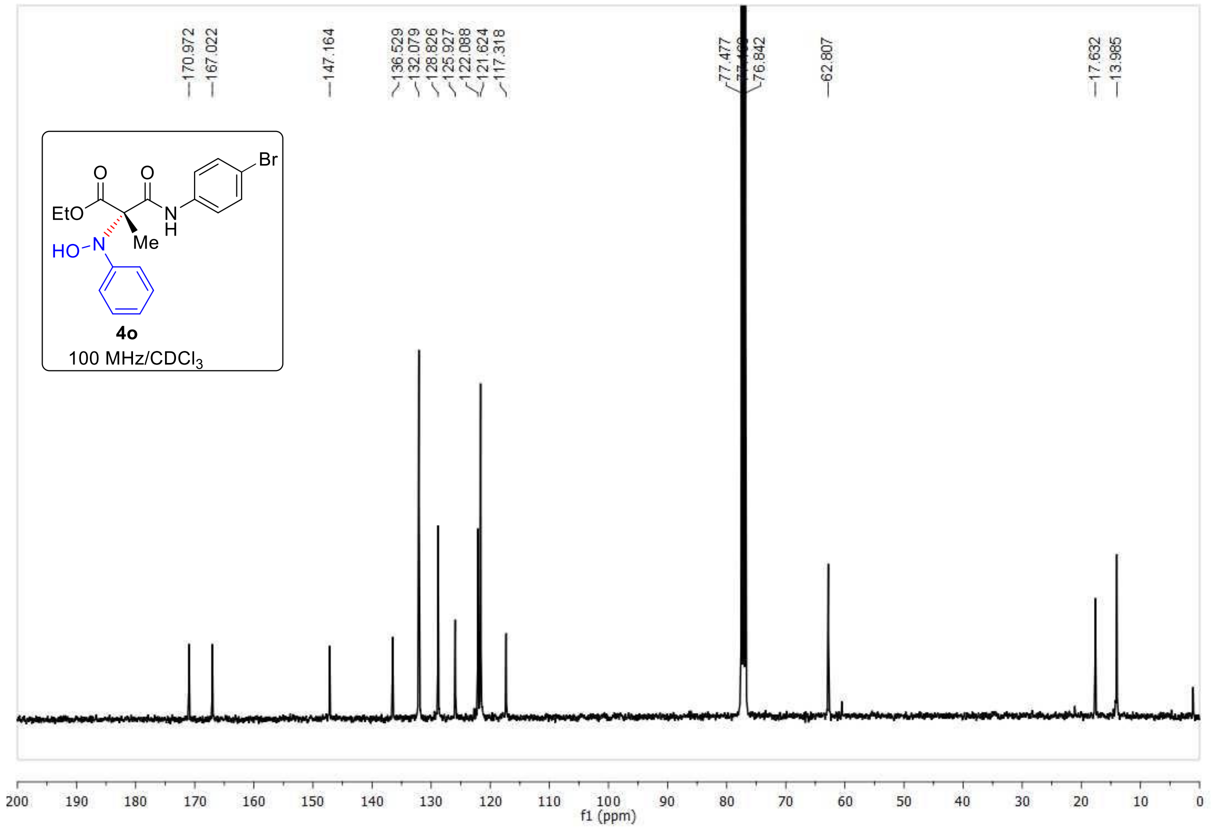
170.972
167.022
147.164
136.529
132.079
128.826
125.927
122.088
121.624
117.318
77.477
76.842
62.807
17.632
13.985
EtO
O
O
N
H
Br
HO
N
Me
4o
100 MHz/CDCl3
f1 (ppm)

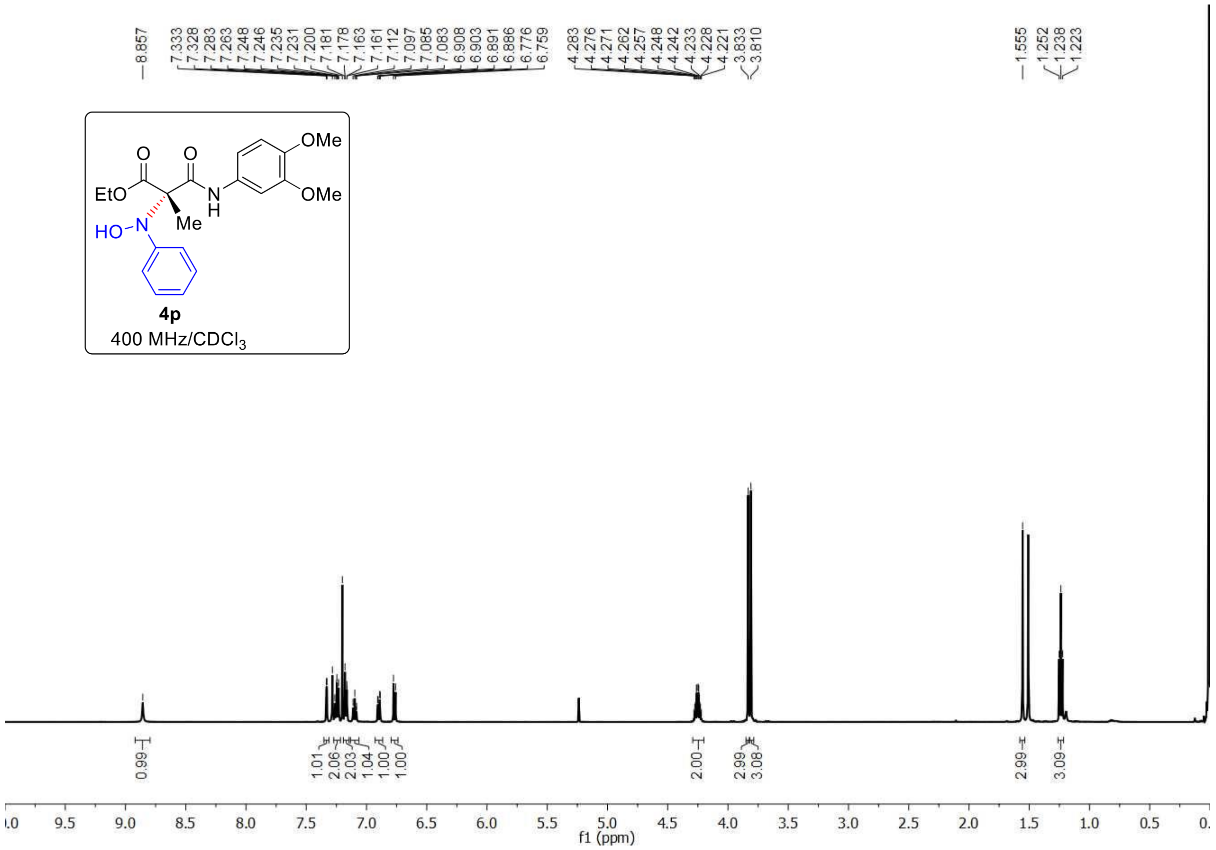

4p
400 MHz/CDCl3
f1 (ppm)

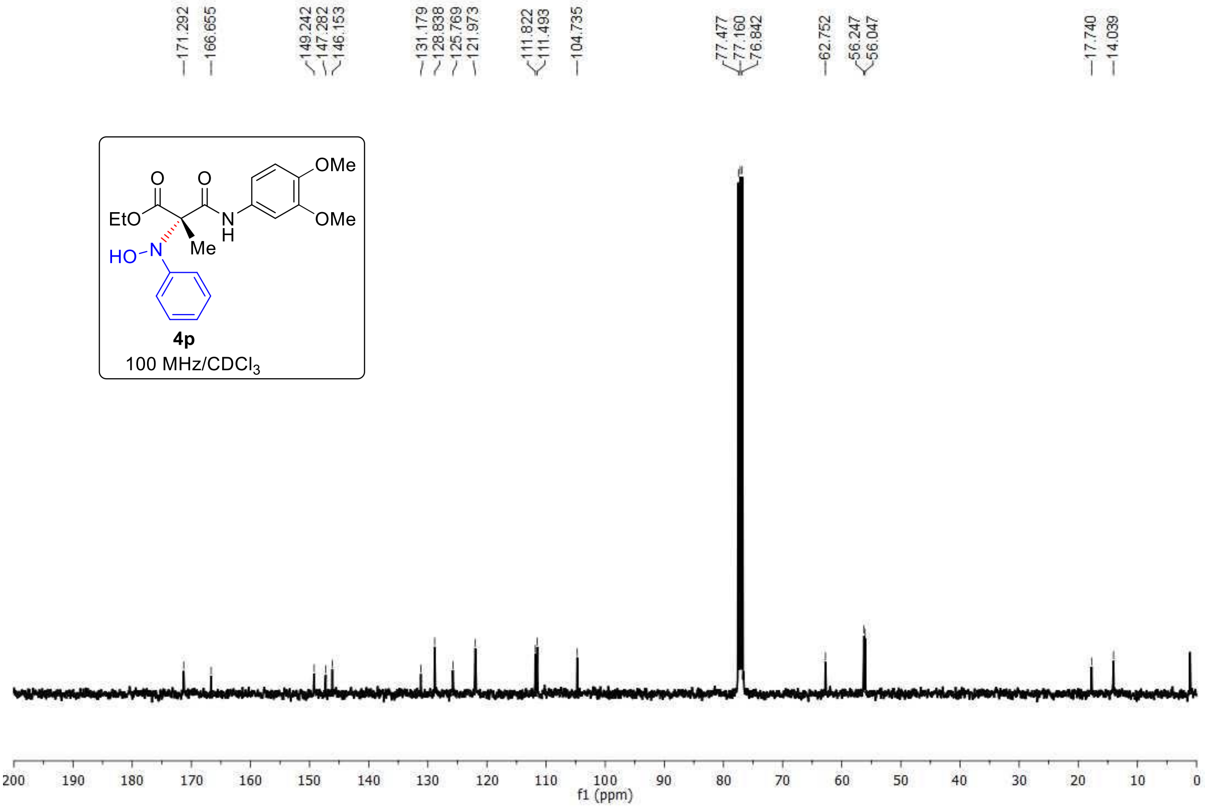

4p
100 MHz/CDCl3
f1 (ppm)

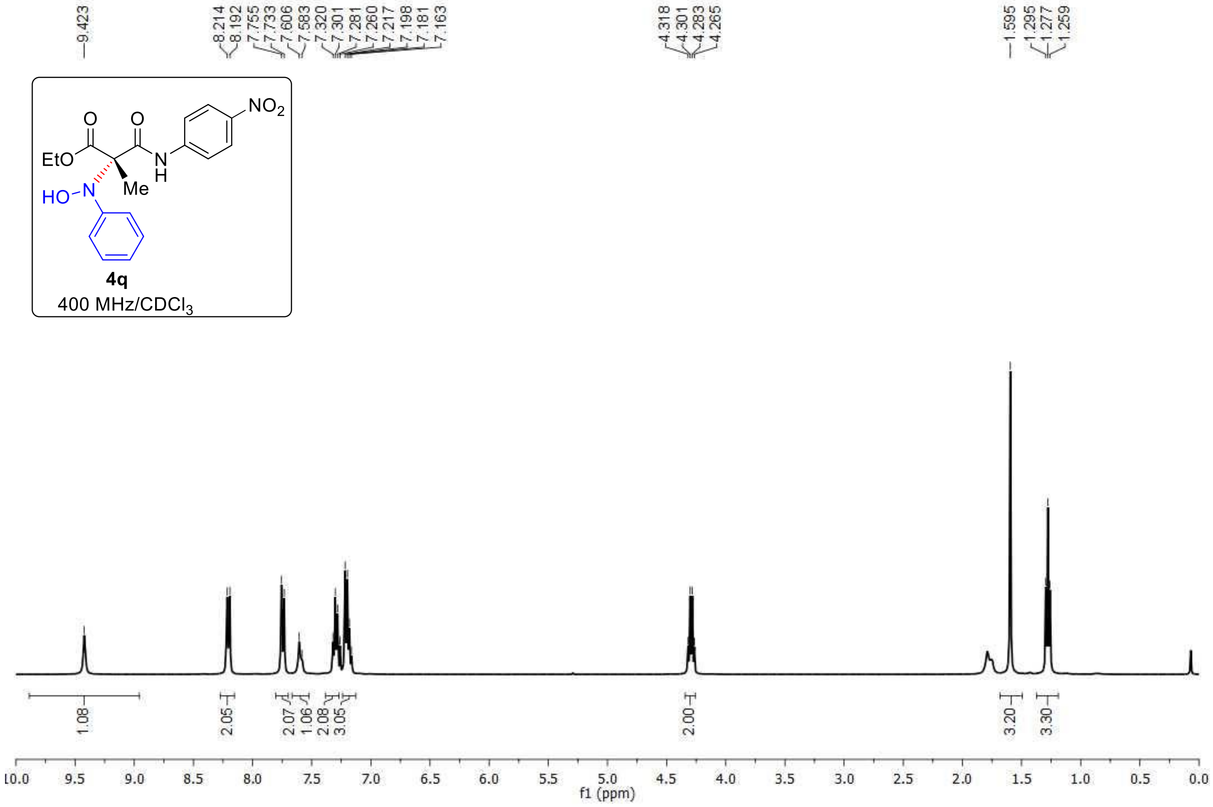
9.423
8.214
8.192
7.755
7.733
7.606
7.583
7.320
7.301
7.281
7.260
7.217
7.198
7.181
7.163
4.318
4.301
4.283
4.265
1.595
1.295
1.277
1.259
NO2
O
O
EtO
N
H
HO
N
Me
4q
400 MHz/CDCl3
1.08
2.05
2.07
1.06
2.08
3.05
2.00
3.20
3.30
f1 (ppm)

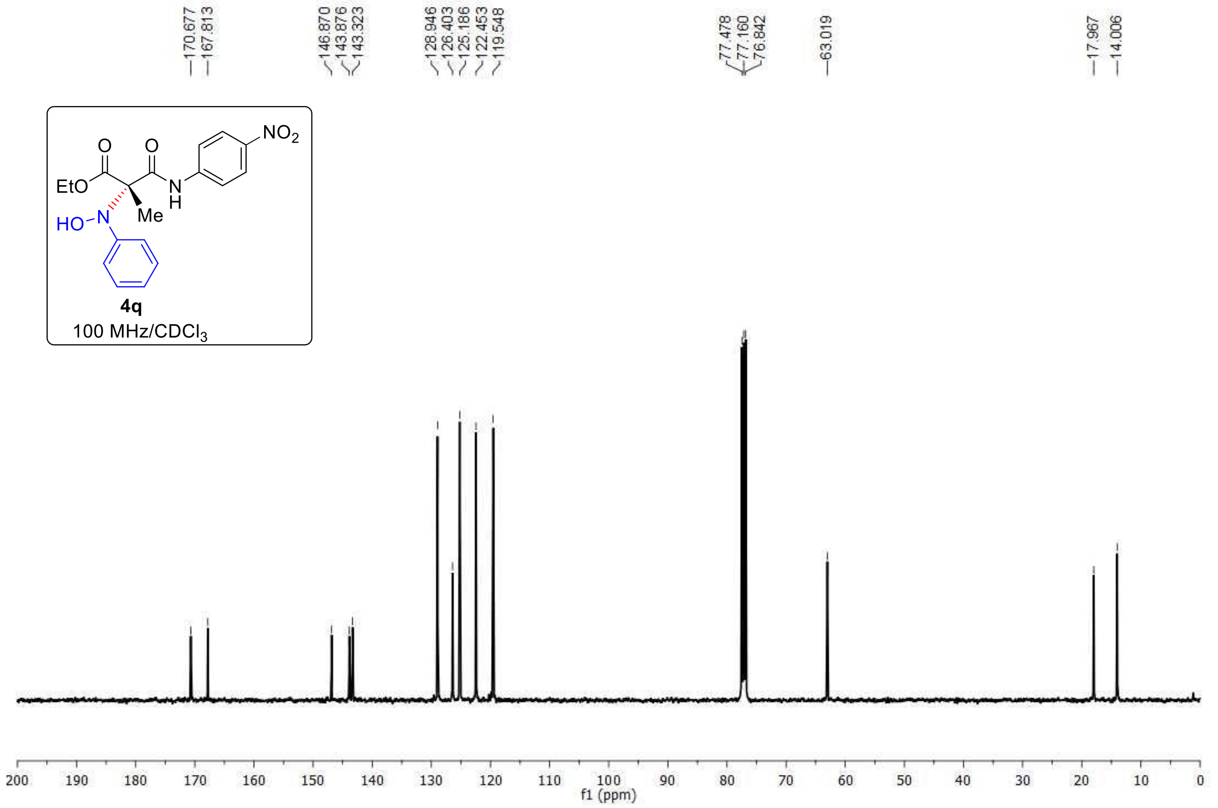
170.677
167.813
146.870
143.876
143.323
128.946
126.403
125.186
122.453
119.548
77.478
77.160
76.842
63.019
17.967
14.006
NO2
O
O
EtO
N
H
HO
N
Me
4q
100 MHz/CDCl3
f1 (ppm)

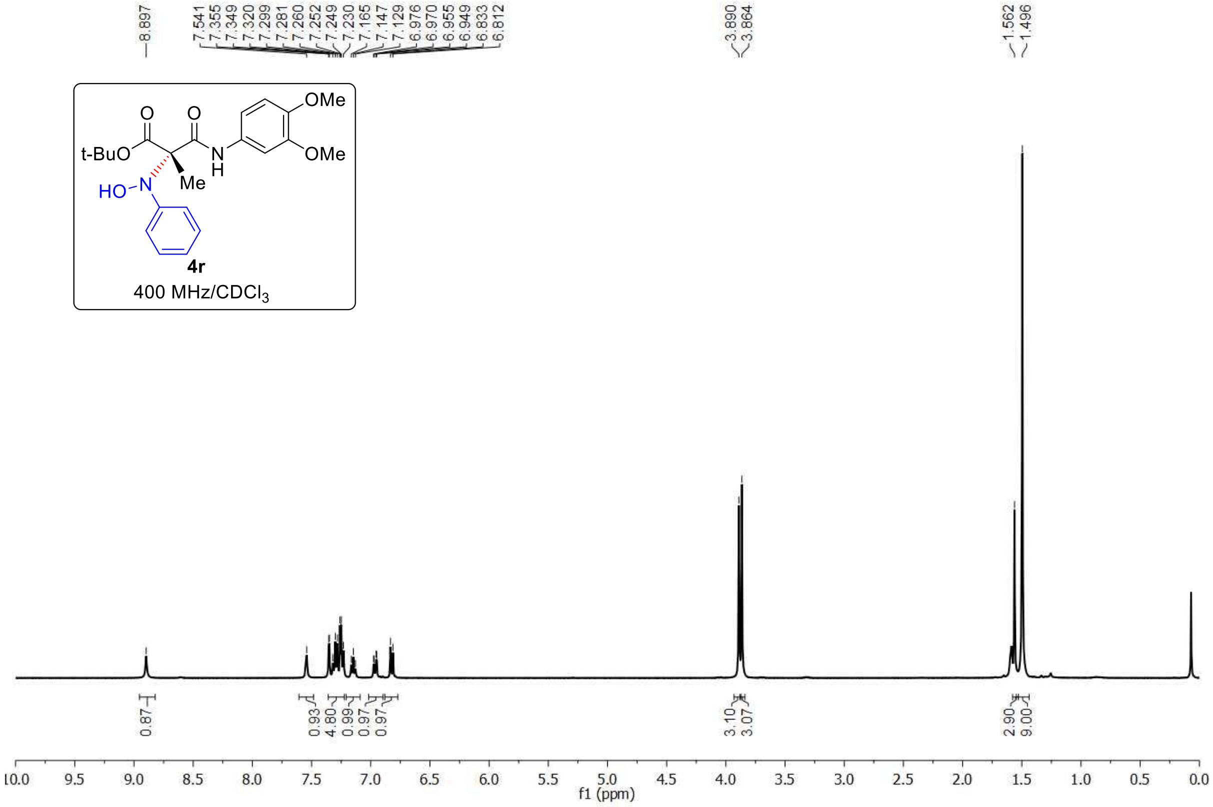
8.897
7.541
7.355
7.349
7.320
7.299
7.281
7.260
7.252
7.249
7.230
7.165
7.147
7.129
6.976
6.970
6.955
6.949
6.833
6.812
3.890
3.864
1.562
1.496
OMe
O
O
t-BuO
N
H
OMe
HO
N
Me
4r
400 MHz/CDCl3
0.87
0.93
4.80
0.99
0.97
0.97
3.10
3.07
2.90
9.00
f1 (ppm)

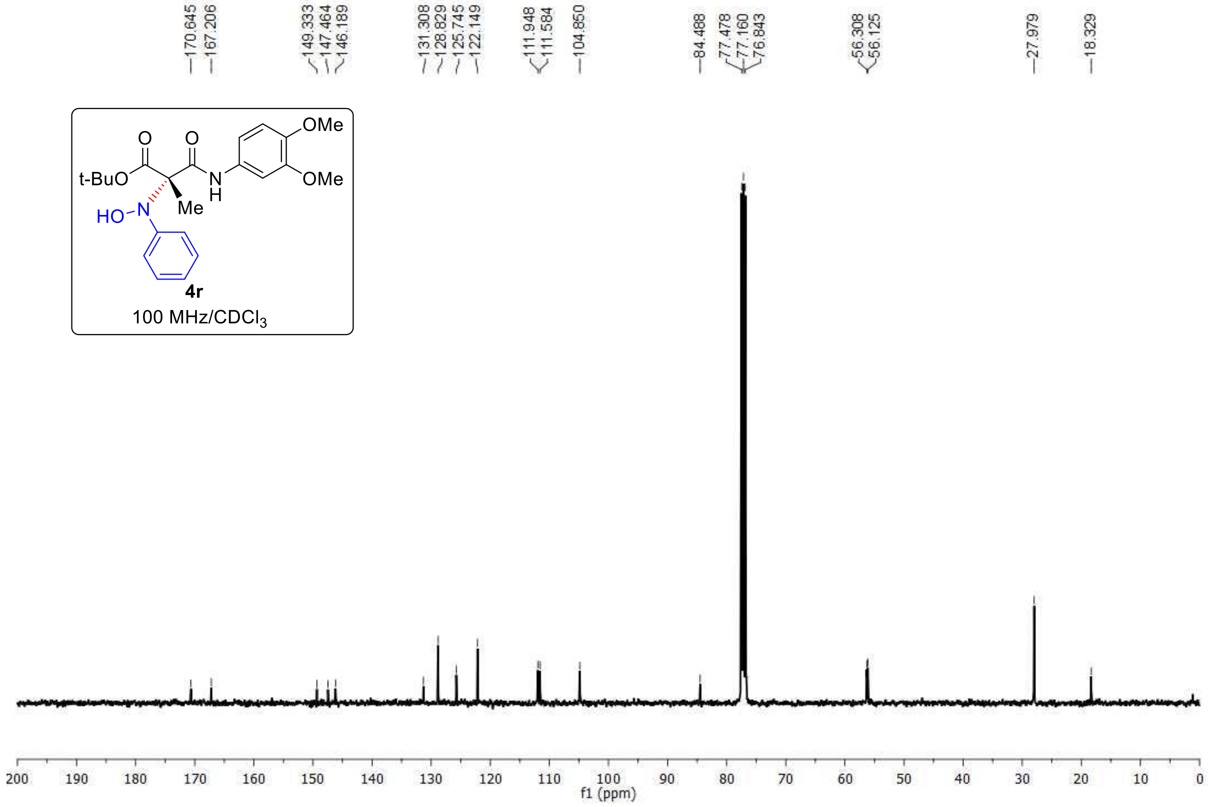
170.645
167.206
149.333
147.464
146.189
131.308
128.829
125.745
122.149
111.948
111.584
104.850
84.488
77.478
77.160
76.843
56.308
56.125
27.979
18.329
OMe
O
O
t-BuO
N
H
OMe
HO
N
Me
4r
100 MHz/CDCl3
f1 (ppm)

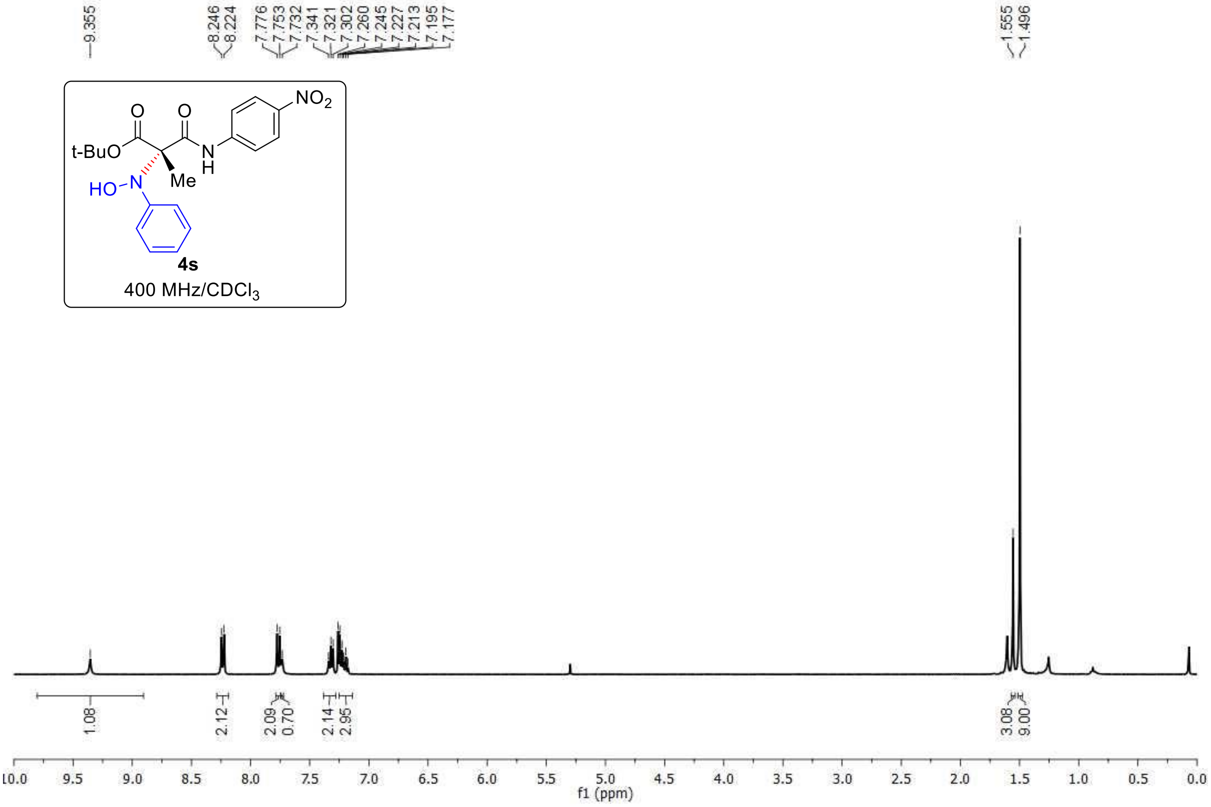
9.355
8.246
8.224
7.776
7.753
7.732
7.341
7.321
7.302
7.260
7.245
7.227
7.213
7.195
7.177
1.555
1.496
NO2
O
O
t-BuO
N
H
HO
N
Me
4s
400 MHz/CDCl3
1.08
2.12
2.09
0.70
2.14
2.95
3.08
9.00
f1 (ppm)

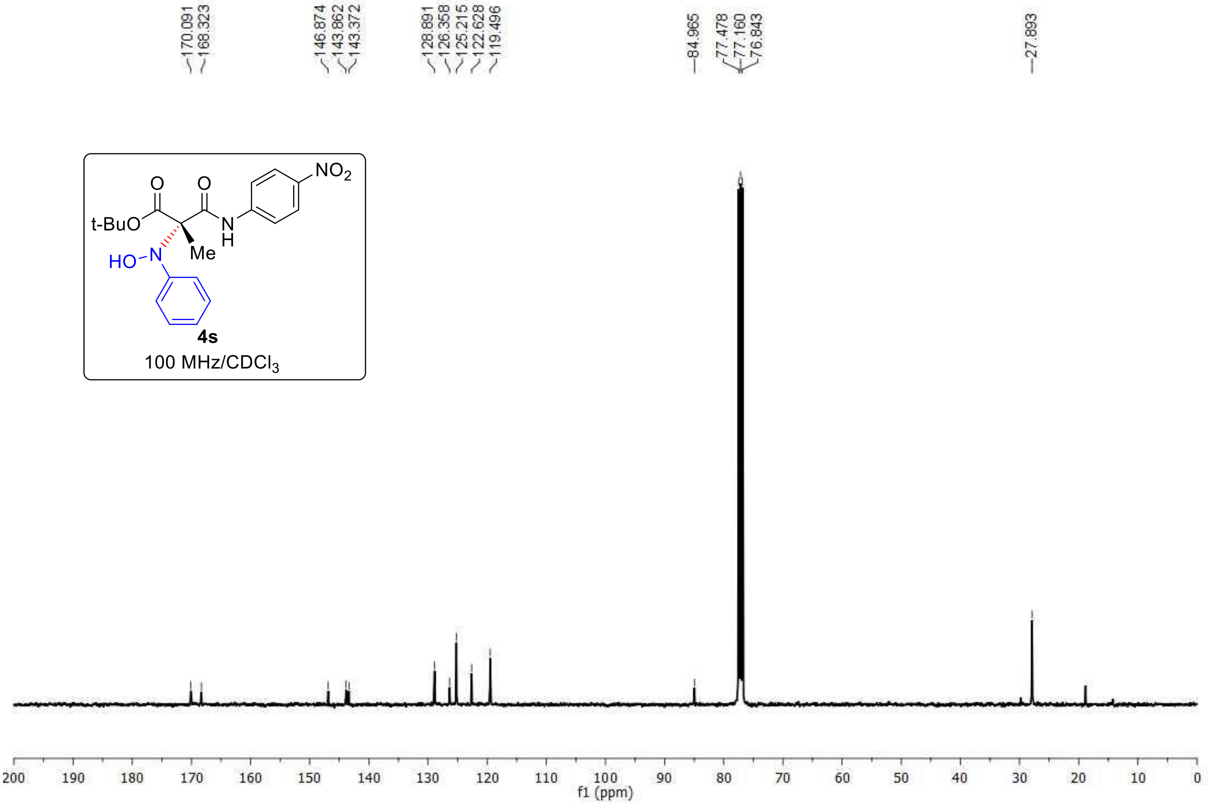
170.091
168.323
146.874
143.862
143.372
128.891
126.358
125.215
122.628
119.496
84.965
77.478
77.160
76.843
27.893
NO2
O
O
t-BuO
N
H
HO
N
Me
4s
100 MHz/CDCl3
f1 (ppm)

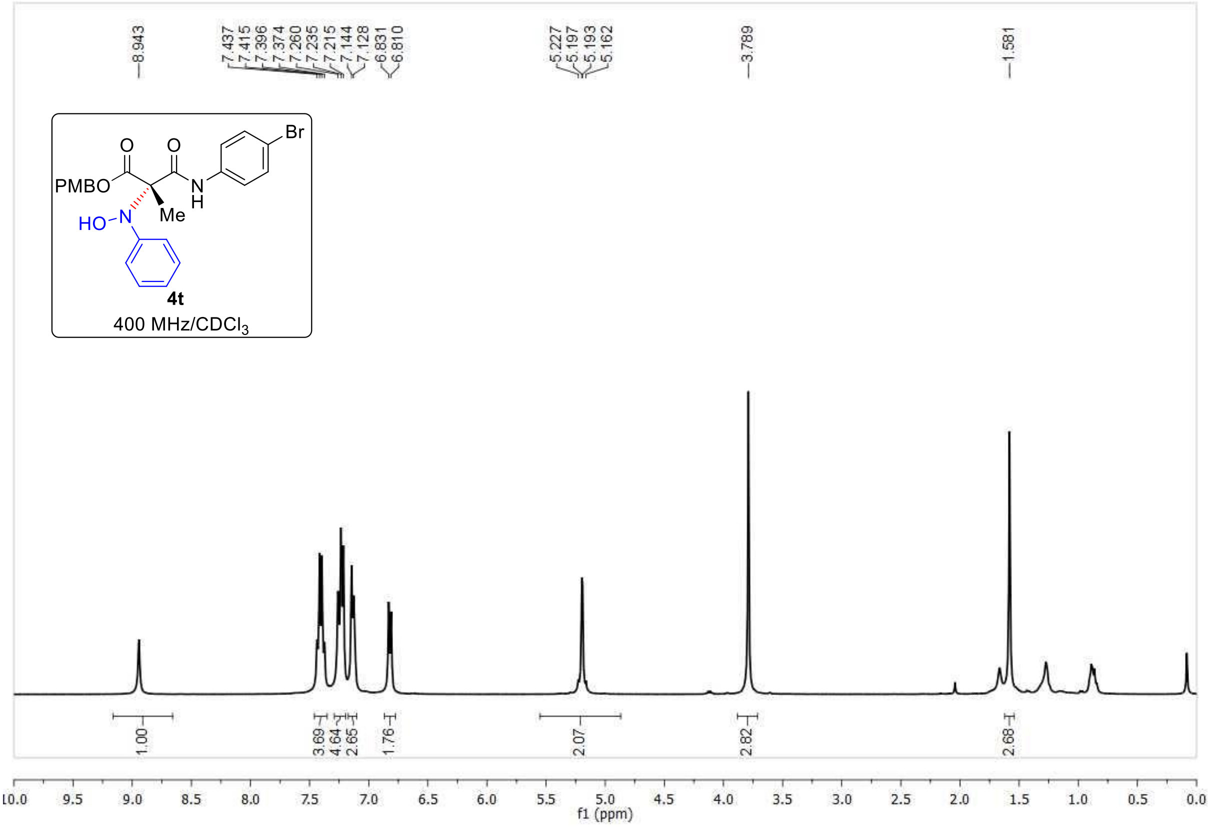

8.943
7.437
7.415
7.396
7.374
7.260
7.235
7.215
7.144
7.128
6.831
6.810
5.227
5.197
5.193
5.162
3.789
1.581
PMBO
O
O
N
H
Br
HO
N
Me
4t
400 MHz/CDCl3
1.00
3.69
4.64
2.65
1.76
2.07
2.82
2.68
f1 (ppm)

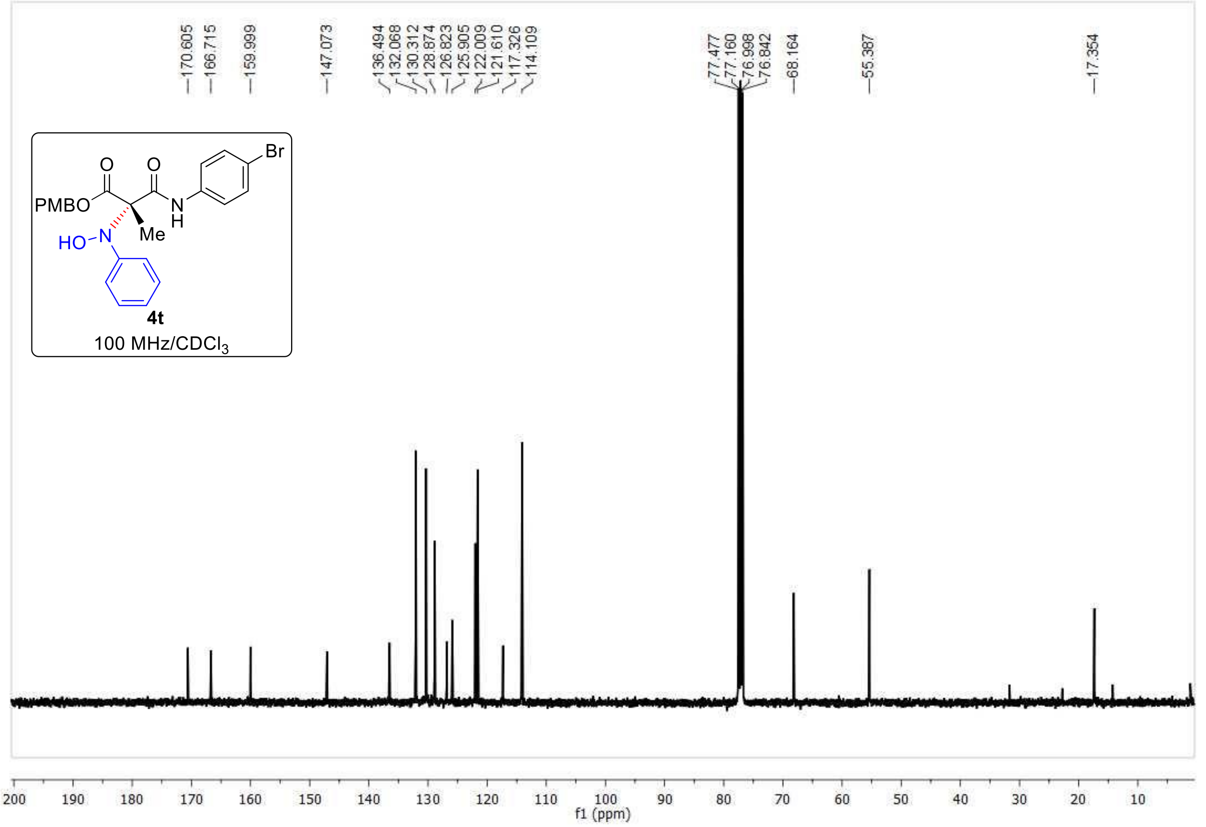

170.605
166.715
159.999
147.073
136.494
132.068
130.312
128.874
126.823
125.905
122.009
121.610
117.326
114.109
77.477
77.160
76.998
76.842
68.164
55.387
17.354
PMBO
O
O
N
H
Br
HO
N
Me
4t
100 MHz/CDCl3
f1 (ppm)

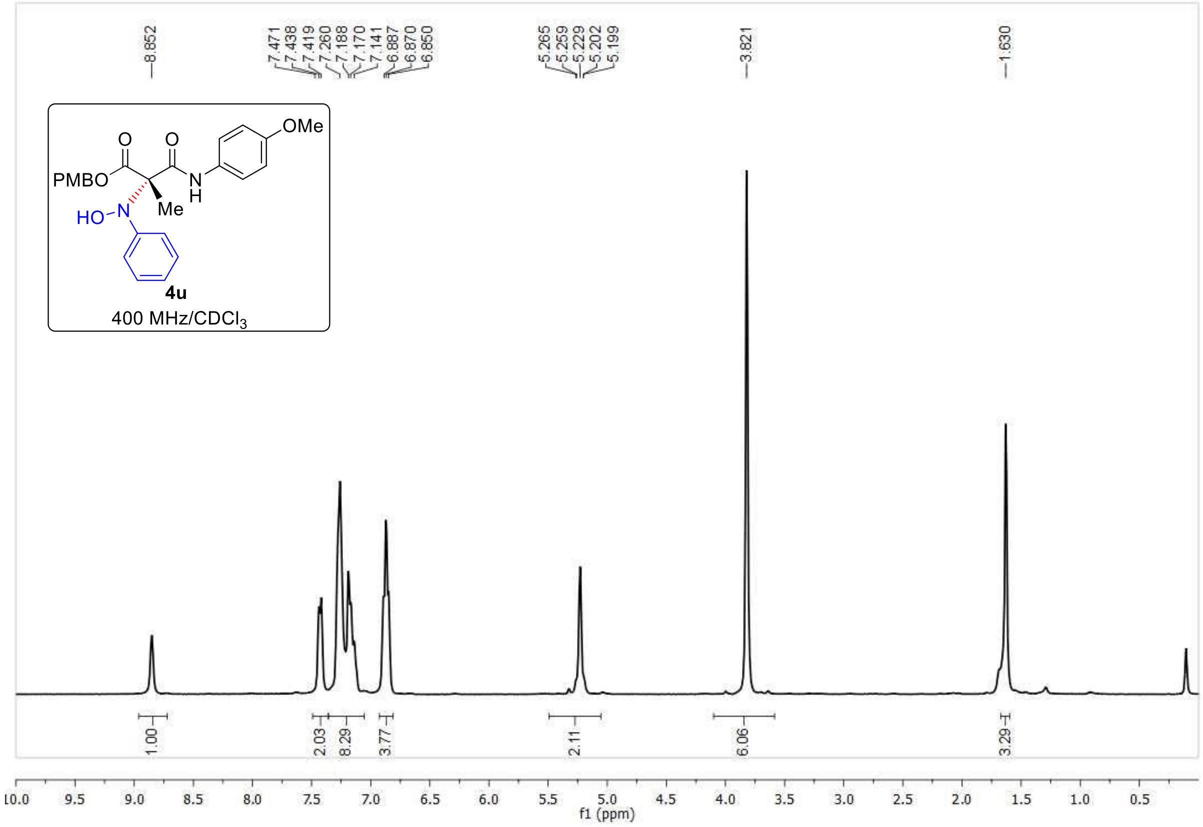
8.852
7.471
7.438
7.419
7.260
7.188
7.170
7.141
6.887
6.870
6.850
5.265
5.259
5.229
5.202
5.199
3.821
1.630
OMe
O
O
PMBO
N
H
HO
N
Me
4u
400 MHz/CDCl3
1.00
2.03
8.29
3.77
2.11
6.06
3.29
f1 (ppm)

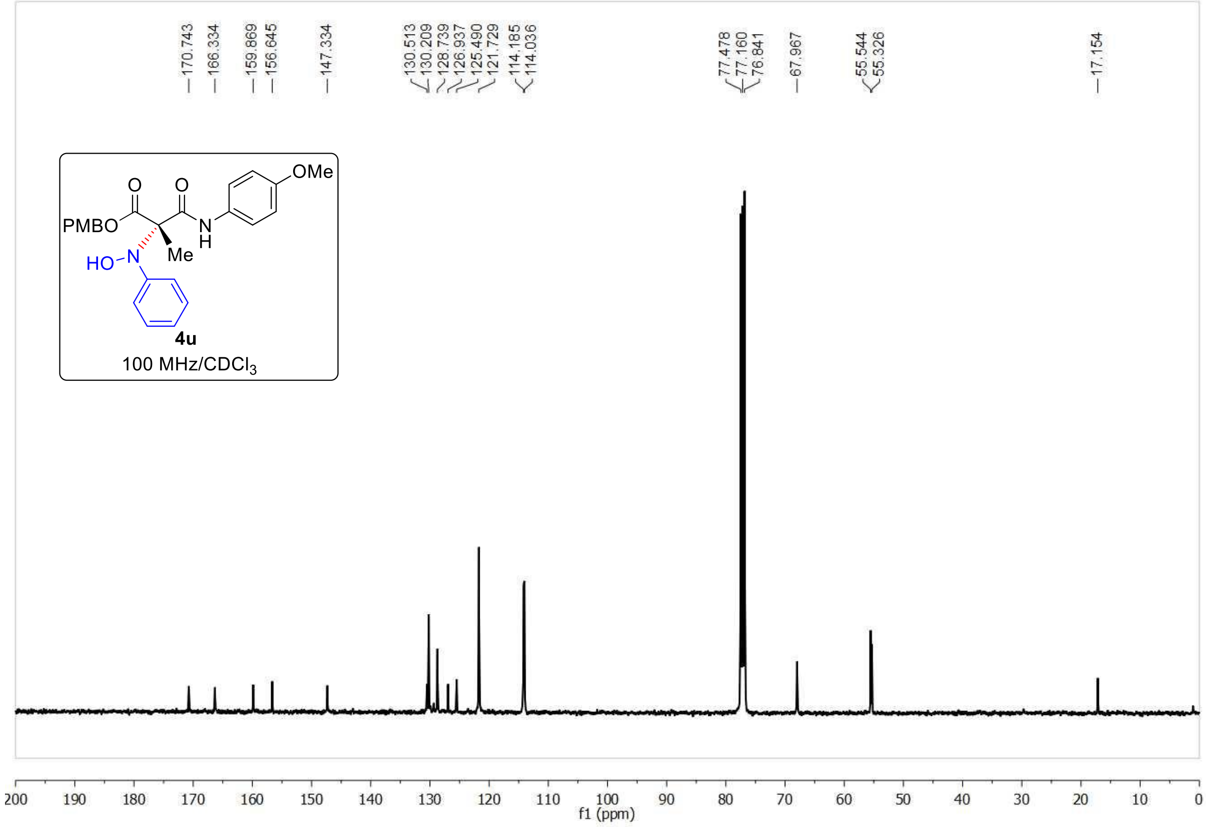
170.743
166.334
159.869
156.645
147.334
130.513
130.209
128.739
126.937
125.490
121.729
114.185
114.036
77.478
77.160
76.841
67.967
55.544
55.326
17.154
OMe
O
O
PMBO
N
H
HO
N
Me
4u
100 MHz/CDCl3
f1 (ppm)

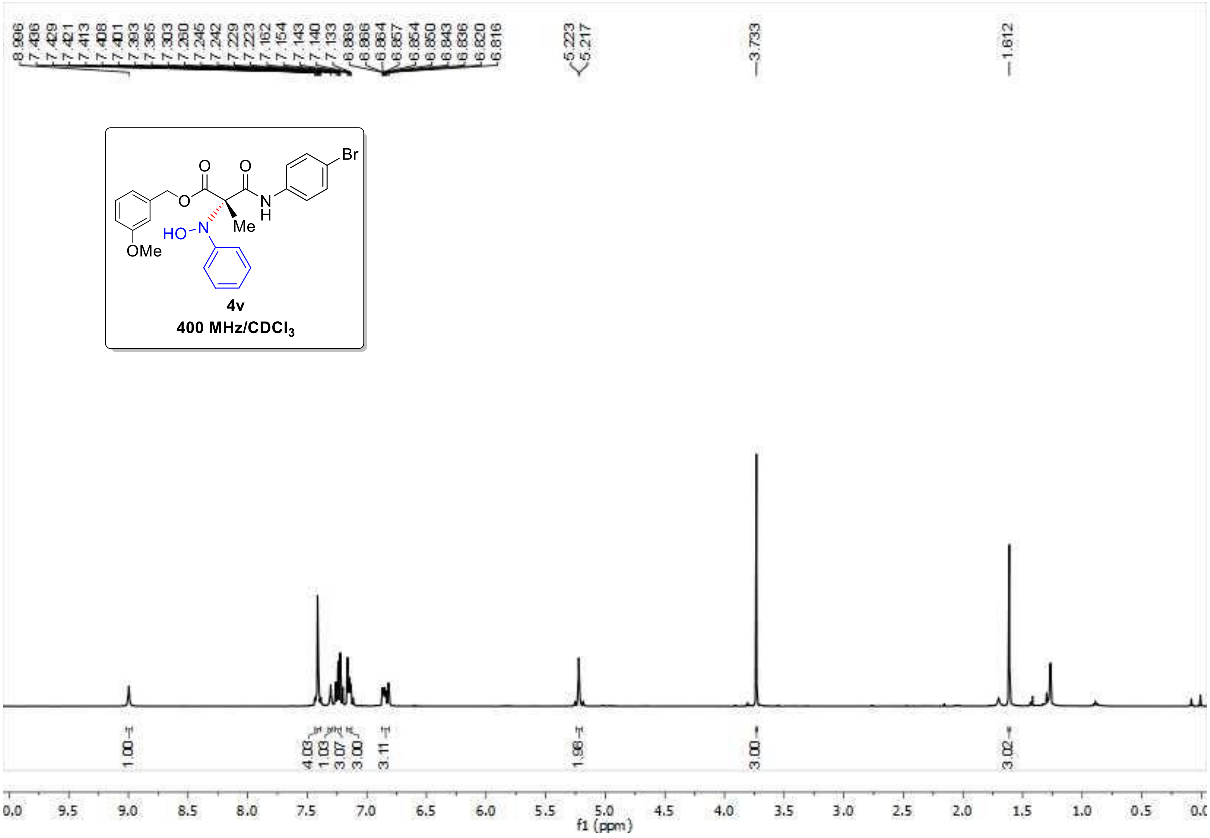
8.996
7.436
7.429
7.421
7.413
7.408
7.401
7.393
7.385
7.303
7.280
7.245
7.242
7.229
7.223
7.162
7.154
7.143
7.140
7.133
6.869
6.866
6.864
6.857
6.854
6.850
6.843
6.836
6.820
6.816
5.223
5.217
3.733
1.612
Br
O
O
O
N
H
HO
N
Me
OMe
4v
400 MHz/CDCl3
1.00
4.03
1.03
3.07
3.00
3.11
1.98
3.00
3.02
f1 (ppm)

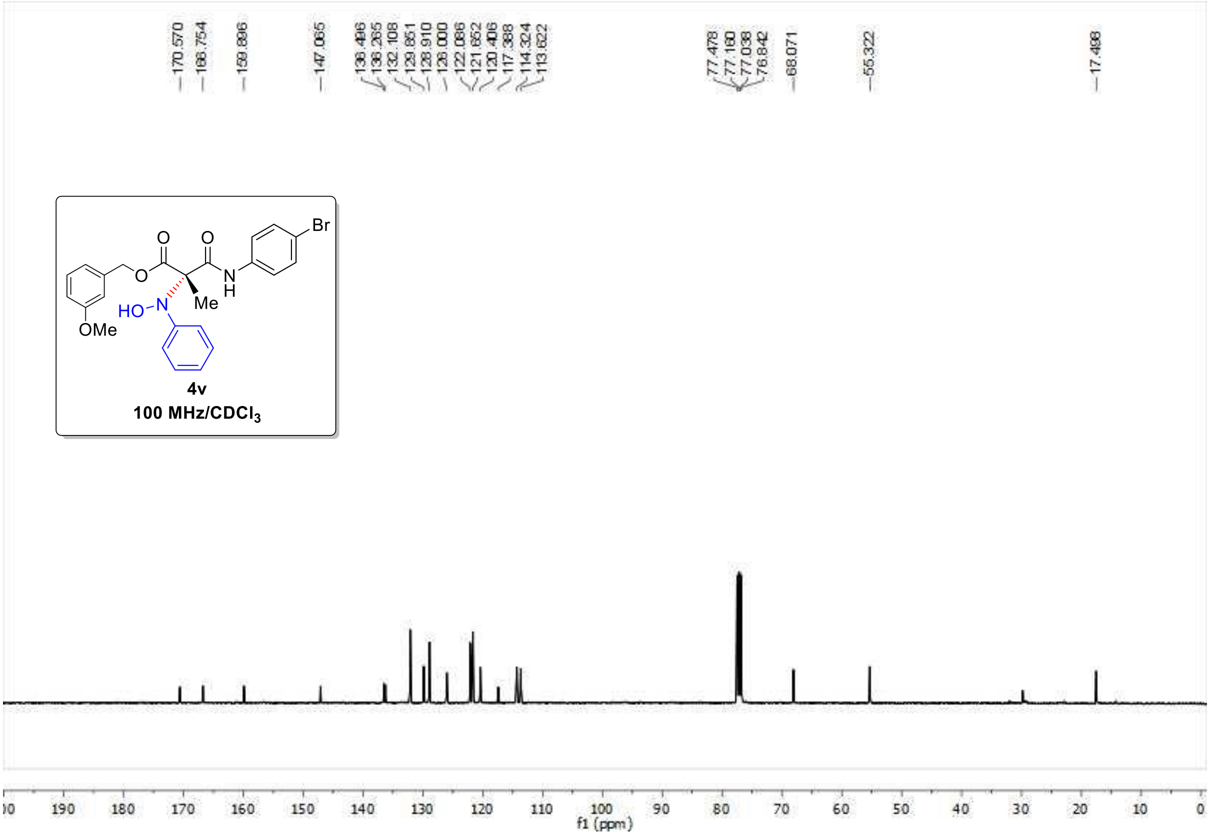
170.570
166.754
159.896
147.065
136.496
136.265
132.108
129.851
128.910
126.000
122.086
121.652
120.406
117.388
114.324
113.622
77.478
77.160
77.038
76.842
68.071
55.322
17.498
Br
O
O
O
N
H
HO
N
Me
OMe
4v
100 MHz/CDCl3
f1 (ppm)

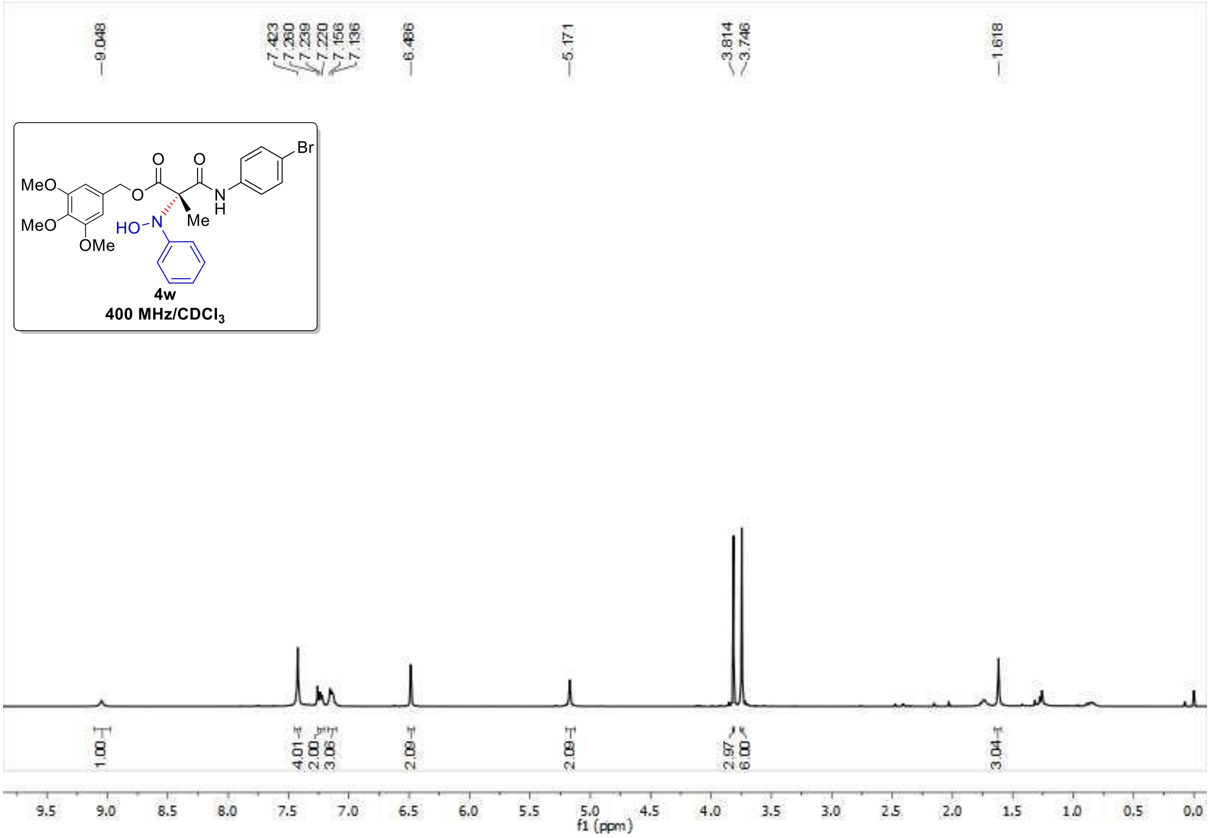

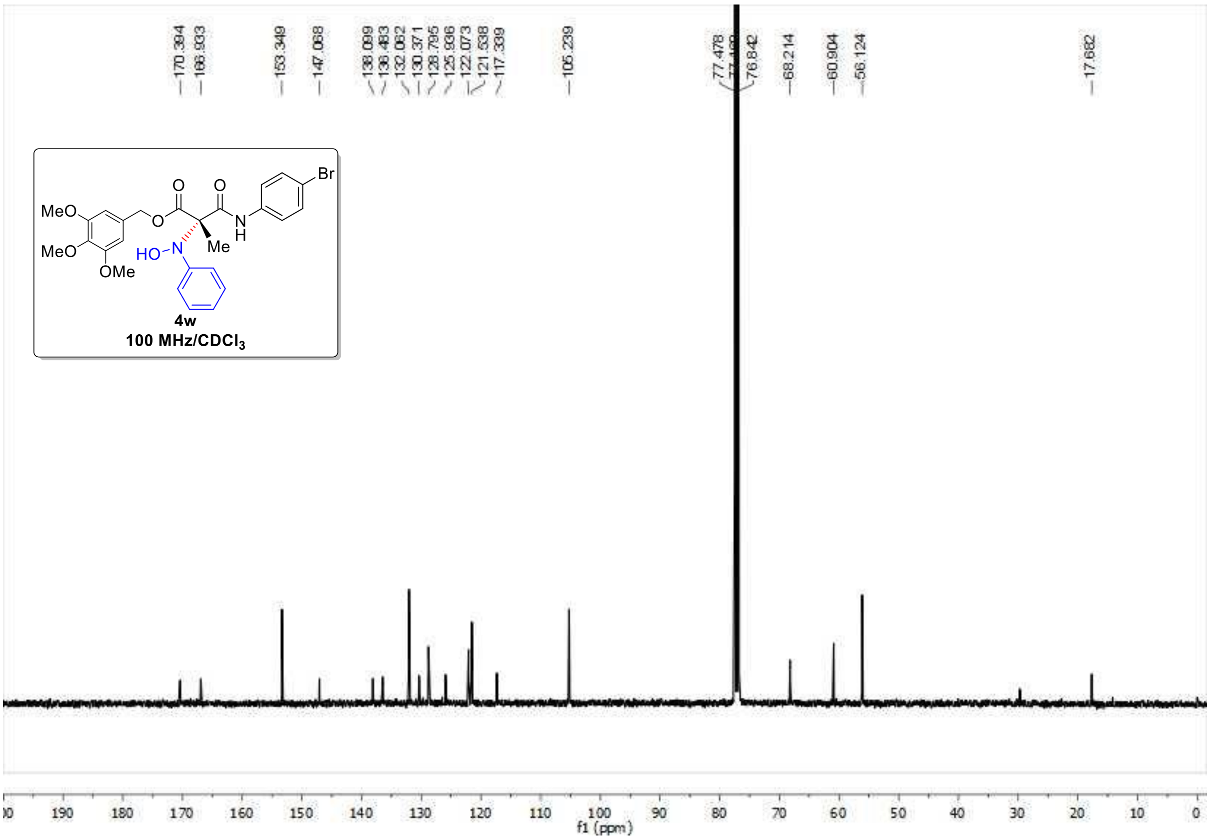

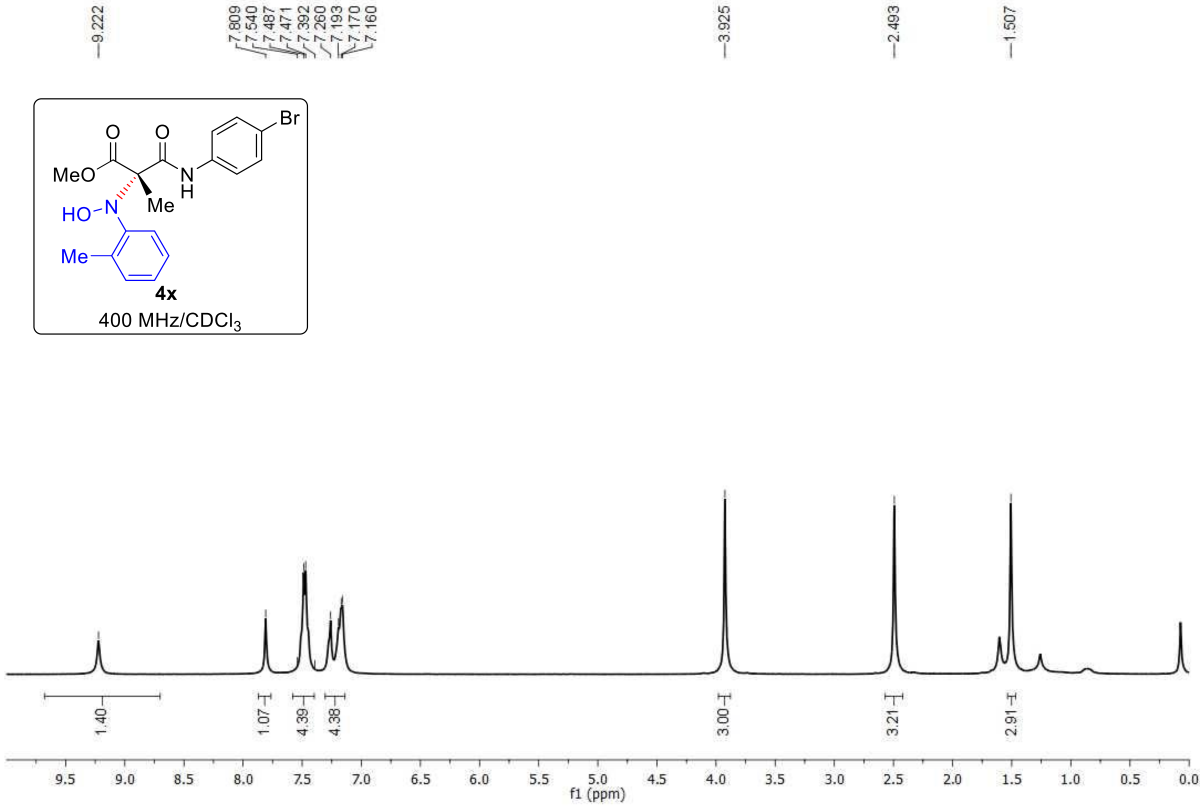

9.222
7.809
7.540
7.487
7.471
7.392
7.260
7.193
7.170
7.160
3.925
2.493
1.507
MeO
O
O
N
H
Br
HO
N
Me
Me
4x
400 MHz/CDCl3
1.40
1.07
4.39
4.38
3.00
3.21
2.91
f1 (ppm)

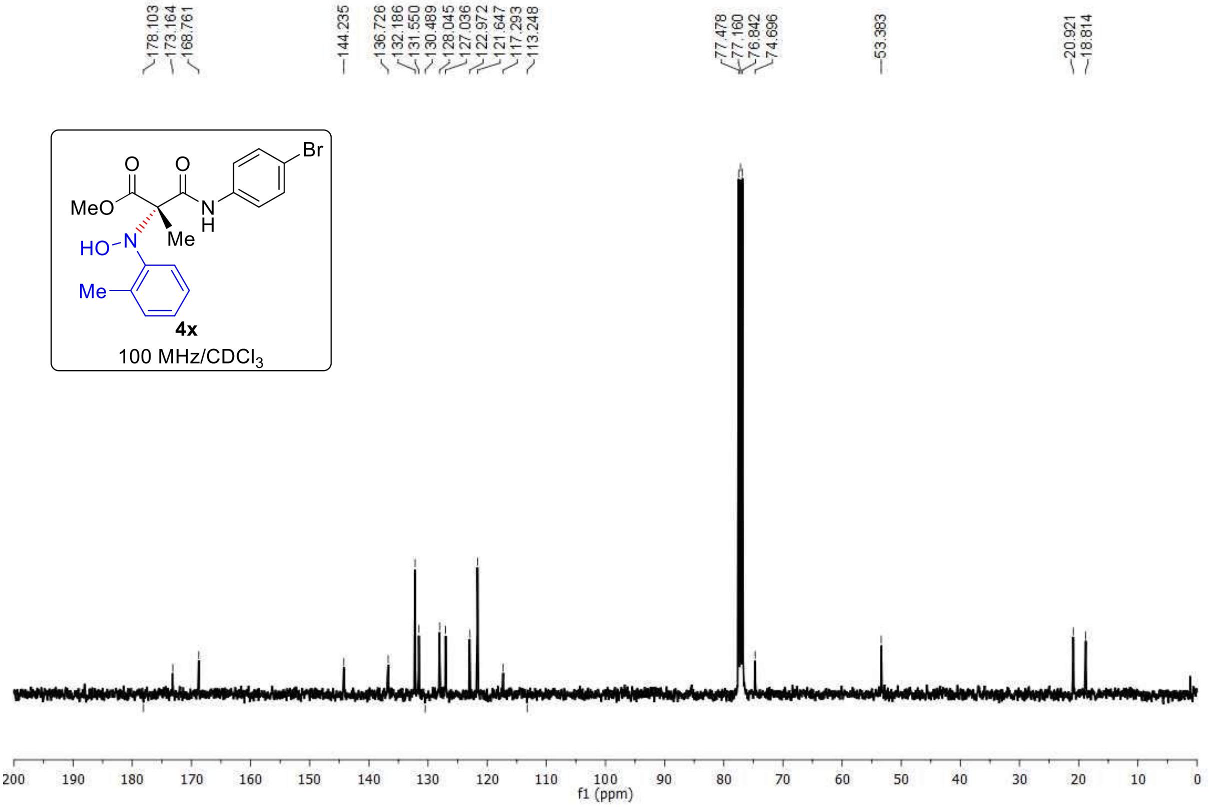

178.103
173.164
168.761
144.235
136.726
132.186
131.550
130.489
128.045
127.036
122.972
121.647
117.293
113.248
77.478
77.160
76.842
74.696
53.383
20.921
18.814
MeO
O
O
N
H
Br
HO
N
Me
Me
4x
100 MHz/CDCl3
f1 (ppm)

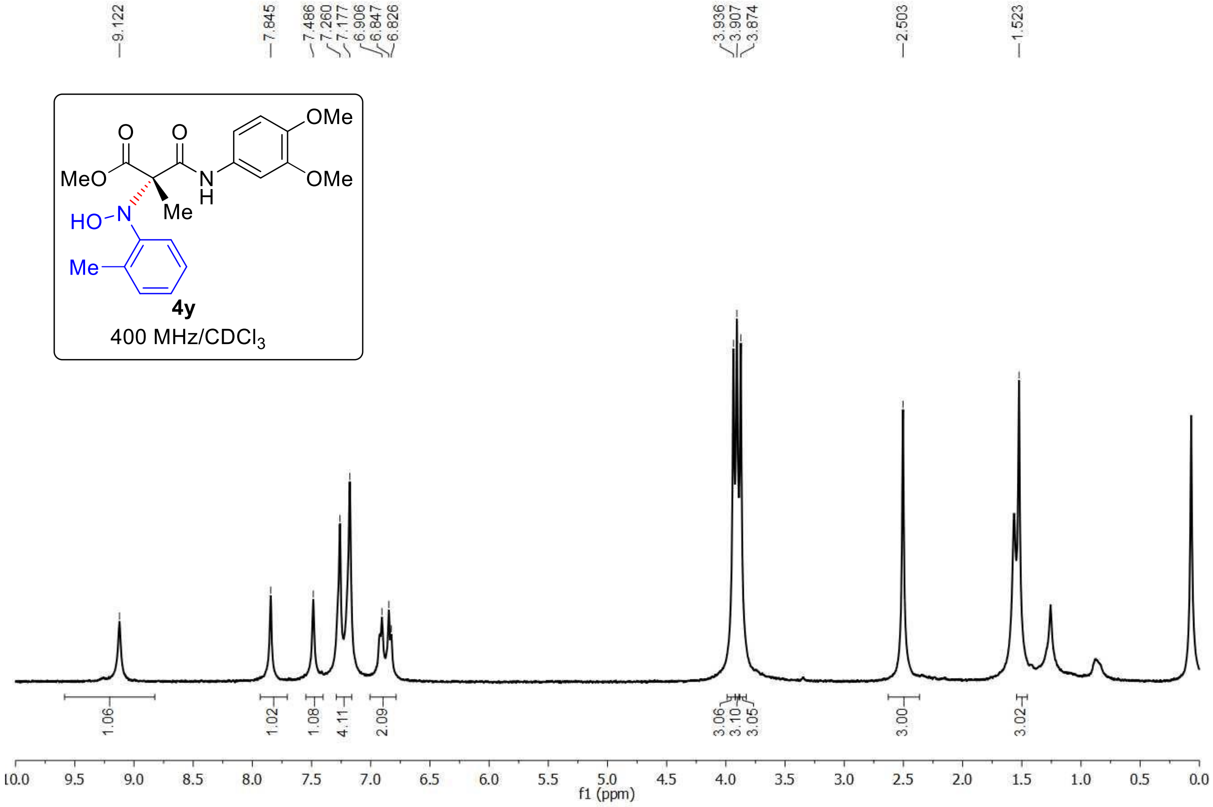

9.122
7.845
7.486
7.260
7.177
6.906
6.847
6.826
3.936
3.907
3.874
2.503
1.523
OMe
O
O
MeO
N
H
OMe
HO
N
Me
Me
4y
400 MHz/CDCl3
1.06
1.02
1.08
4.11
2.09
3.06
3.10
3.05
3.00
3.02
f1 (ppm)

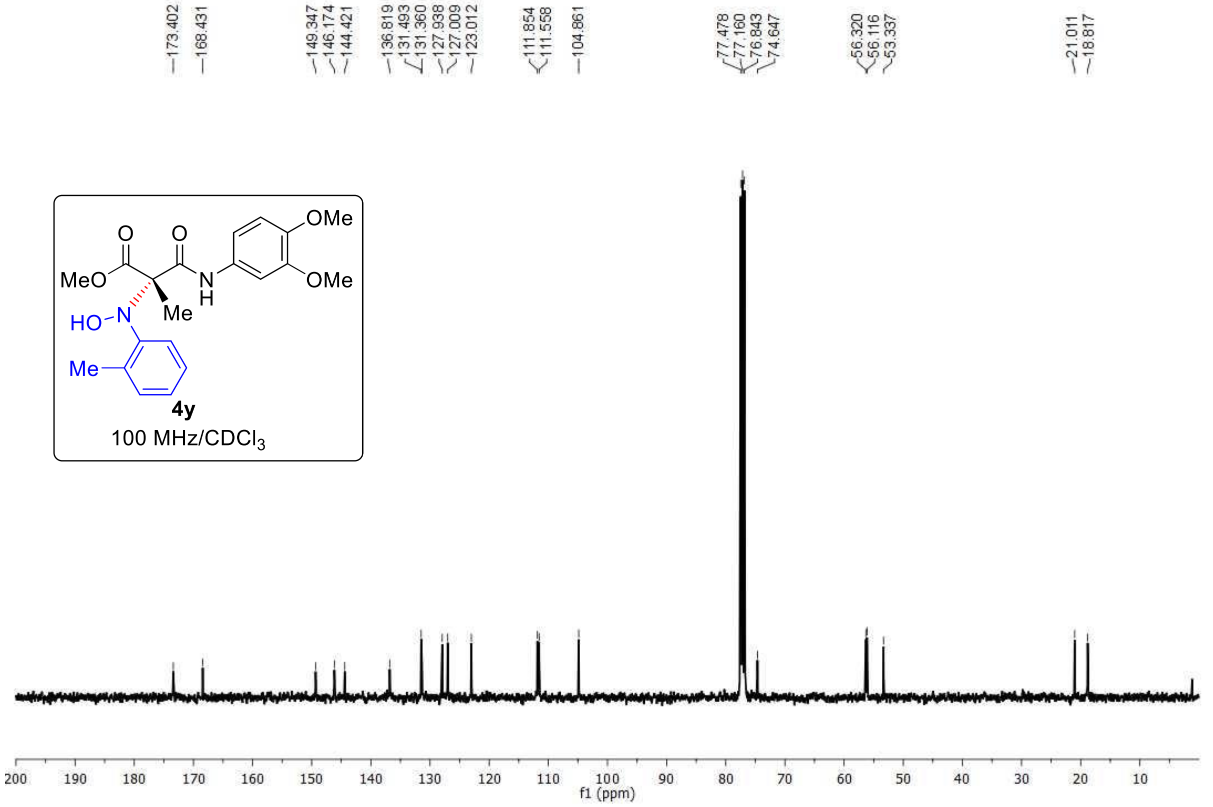

173.402
168.431
149.347
146.174
144.421
136.819
131.493
131.360
127.938
127.009
123.012
111.854
111.558
104.861
77.478
77.160
76.843
74.647
56.320
56.116
53.337
21.011
18.817
OMe
O
O
MeO
N
H
OMe
HO
N
Me
Me
4y
100 MHz/CDCl3
f1 (ppm)

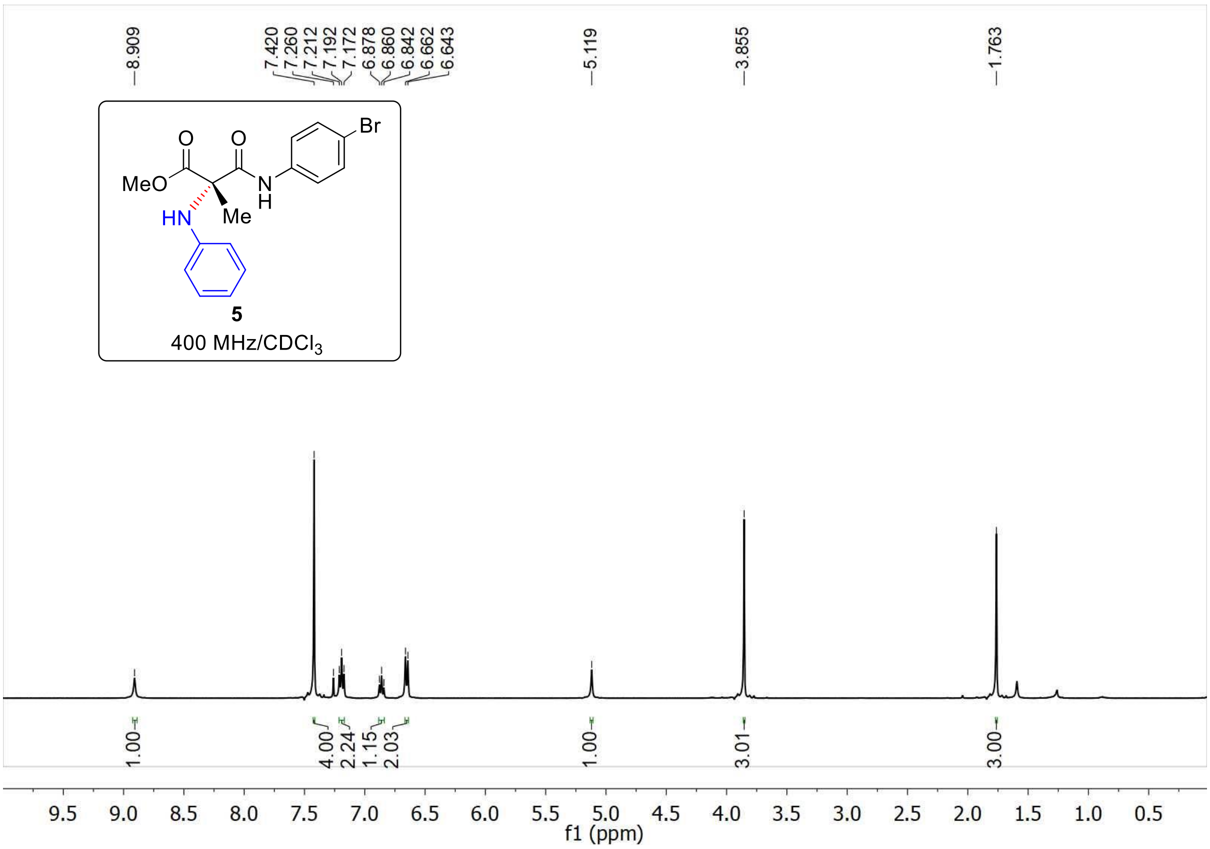
8.909
7.420
7.260
7.212
7.192
7.172
6.878
6.860
6.842
6.662
6.643
5.119
3.855
1.763
MeO
O
O
N
H
Br
HN
Me
5
400 MHz/CDCl3
1.00
4.00
2.24
1.15
2.03
1.00
3.01
3.00
9.5
9.0
8.5
8.0
7.5
7.0
6.5
6.0
5.5
5.0
4.5
4.0
3.5
3.0
2.5
2.0
1.5
1.0
0.5
f1 (ppm)

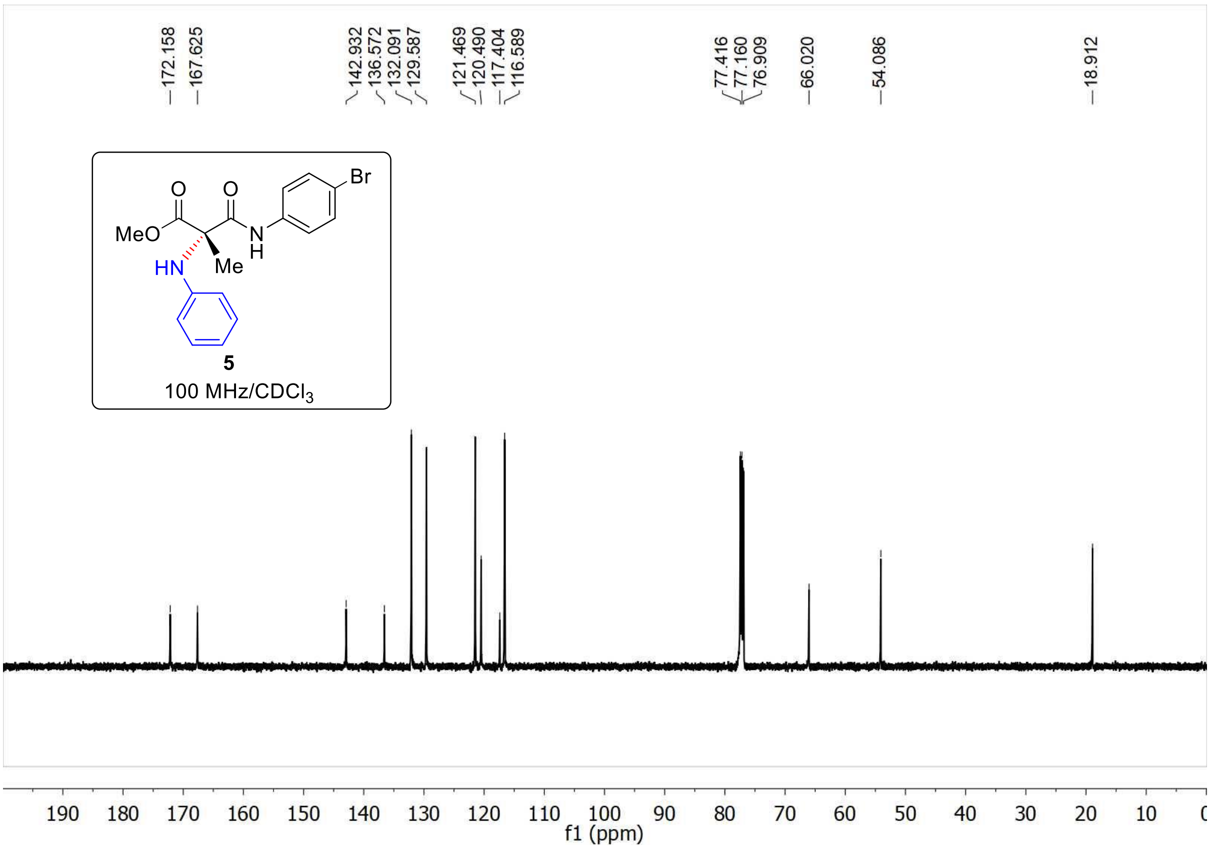
172.158
167.625
142.932
136.572
132.091
129.587
121.469
120.490
117.404
116.589
77.416
77.160
76.909
66.020
54.086
18.912
MeO
O
O
N
H
Br
HN
Me
5
100 MHz/CDCl3
190
180
170
160
150
140
130
120
110
100
90
80
70
60
50
40
30
20
10
f1 (ppm)

HPLC chromatograms of all compounds

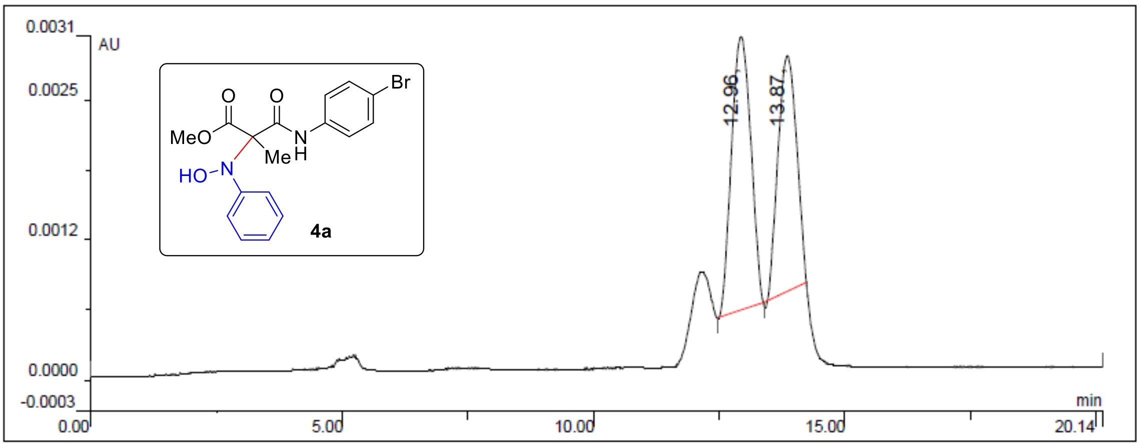

Area % Height %

Sr. No.	Component Name	Ret. Time.	Area µ volt sec	Height µ volt	Area %	Height %	ID	
1		12.96	33221.54	1217	54.82	53.65	U	M
2		13.87	27374.23	1051	45.18	46.35	U	M
			**60595.77**	**2268**	**100.00**	**100.00**		

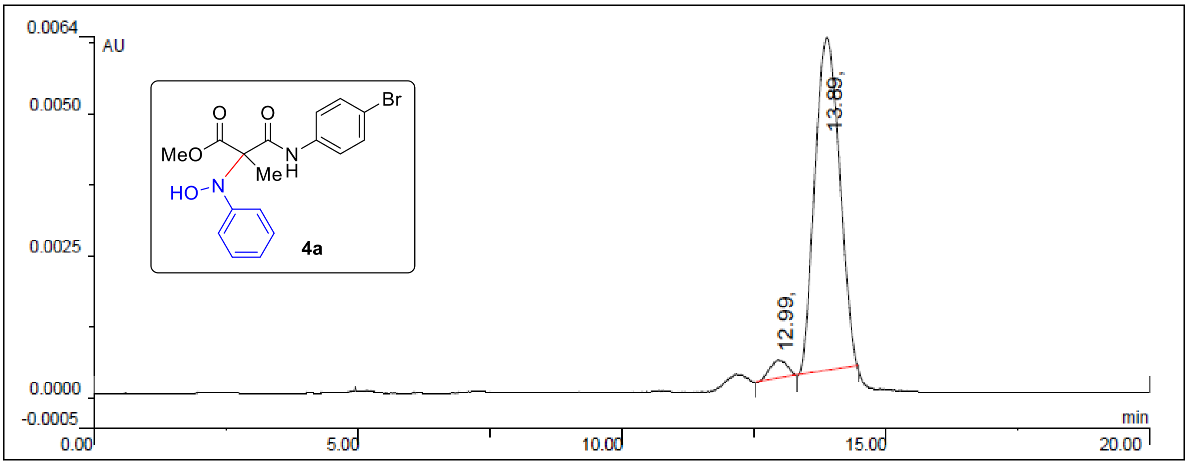

Area % Height %

Sr. No.	Component Name	Ret. Time.	Area µ volt sec	Height µ volt	Area %	Height %	ID	
1		12.99	3523.49	154	3.51	5.00	U	M
2		13.89	96969.54	2931	96.49	95.00	U	M
			**100493.03**	**3085**	**100.00**	**100.00**		

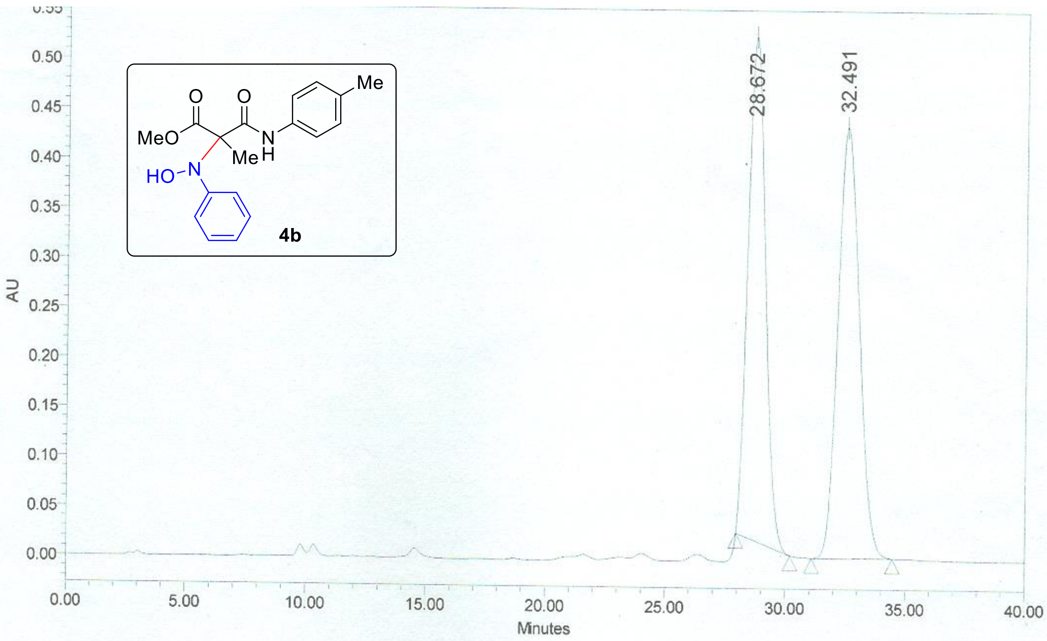

Peak Results

	Name	RT	Area	Height	% Area
1		28.672	25572363	506943	48.72
2		32.491	26916883	433982	51.28

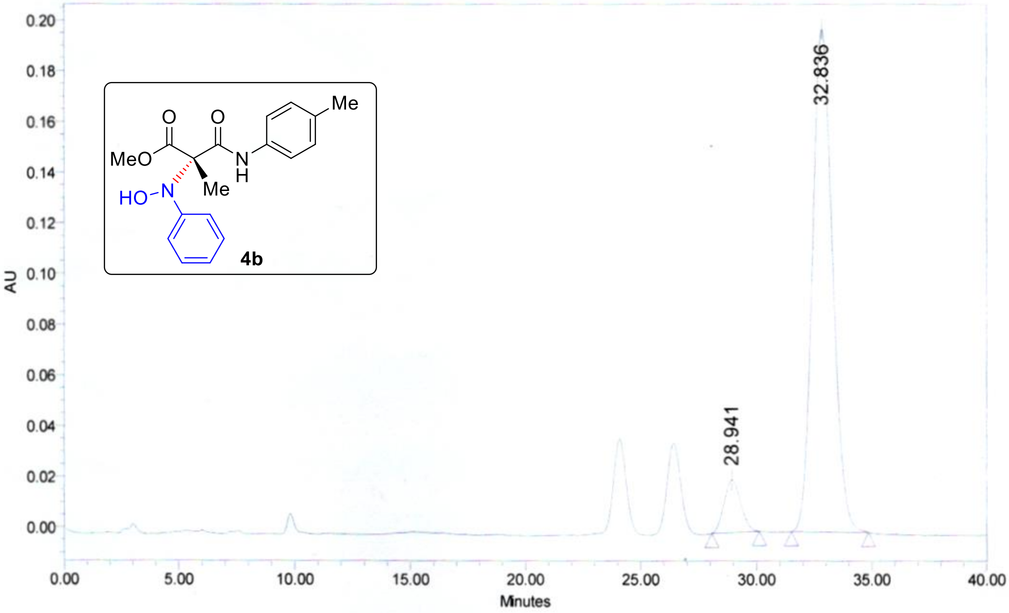

Peak Results

	Name	RT	Area	Height	% Area
1		28.941	1040913	20770	7.98
2		32.836	11996989	198252	92.02

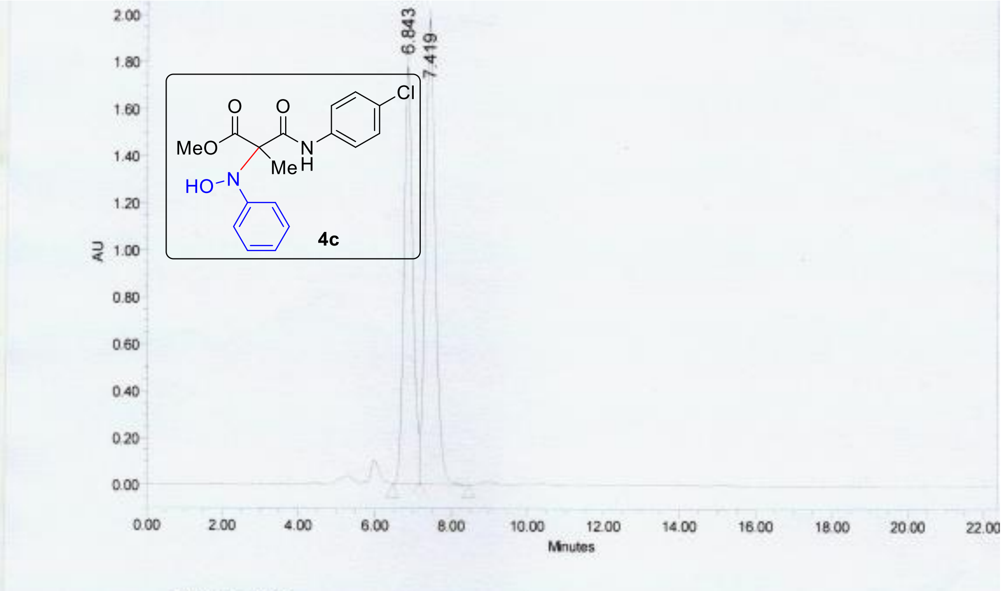

Peak Results

	Name	RT	Area	Height	% Area
1		6.843	28726070	1776853	44.28
2		7.419	36144021	1980070	55.72

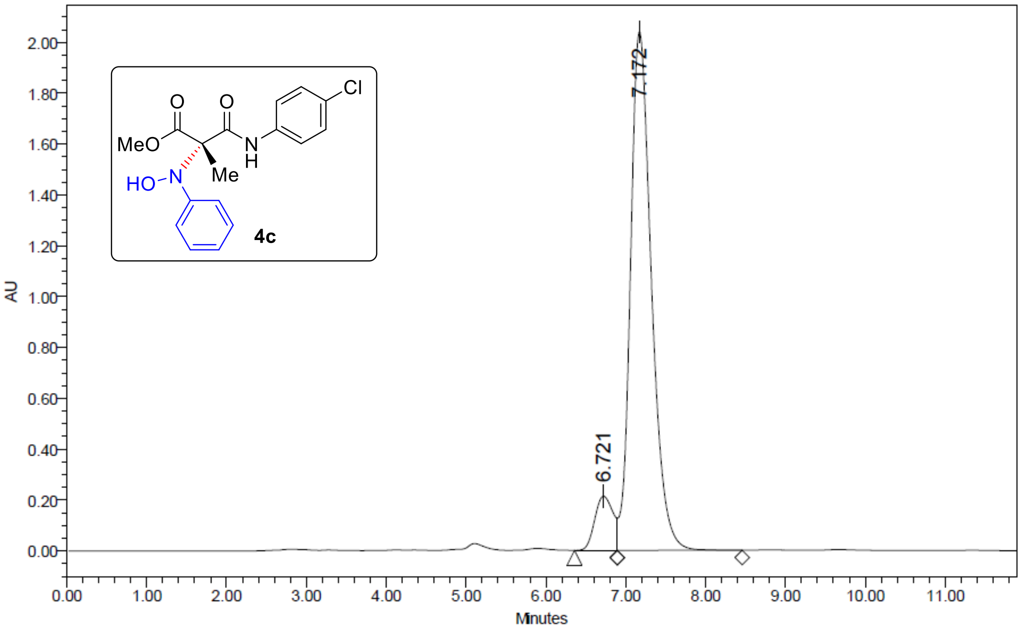

Peak Results

	Name	RT	Area	Height	% Area
1		6.721	3375825	212717	8.32
2		7.172	37218631	2039248	91.68

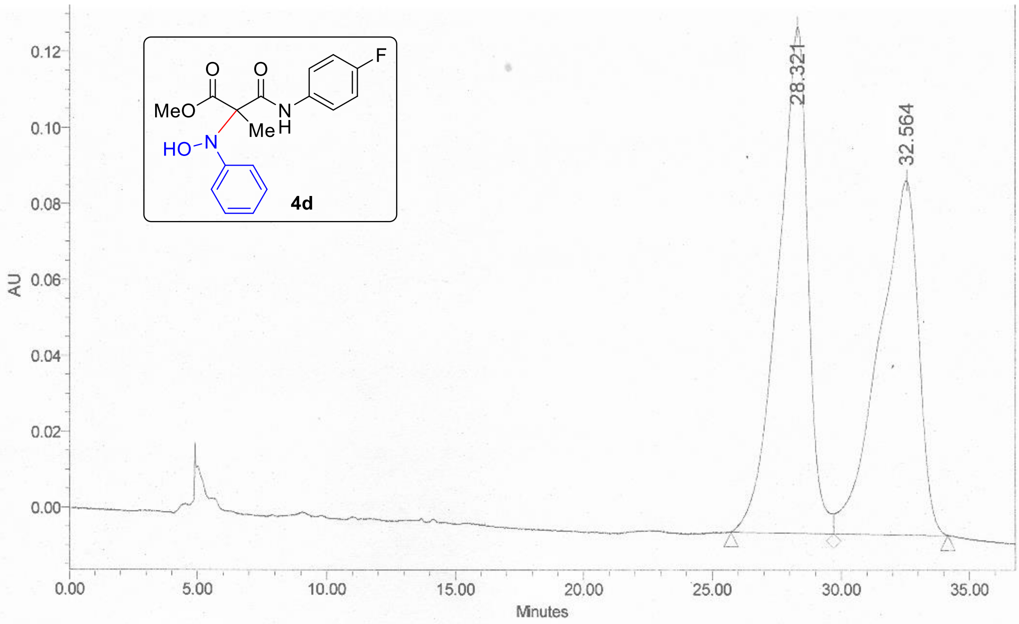

Peak Results

	Name	RT	Area	Height	% Area
1		28.321	10707784	133335	51.62
2		32.564	10036241	93421	48.38

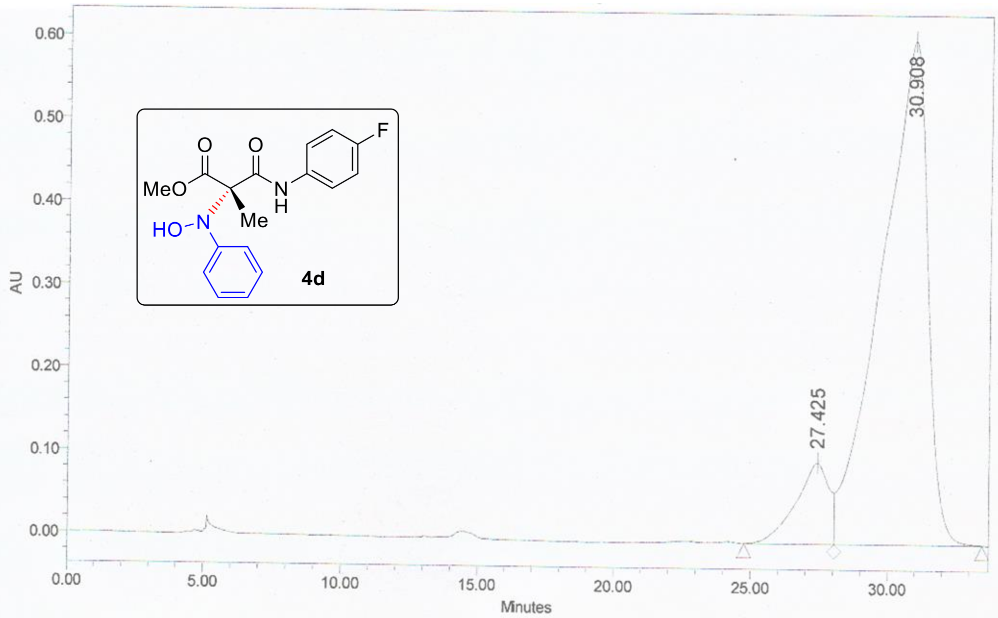

Peak Results

	Name	RT	Area	Height	% Area
1		27.425	8325398	98863	10.37
2		30.908	71947422	608787	89.63

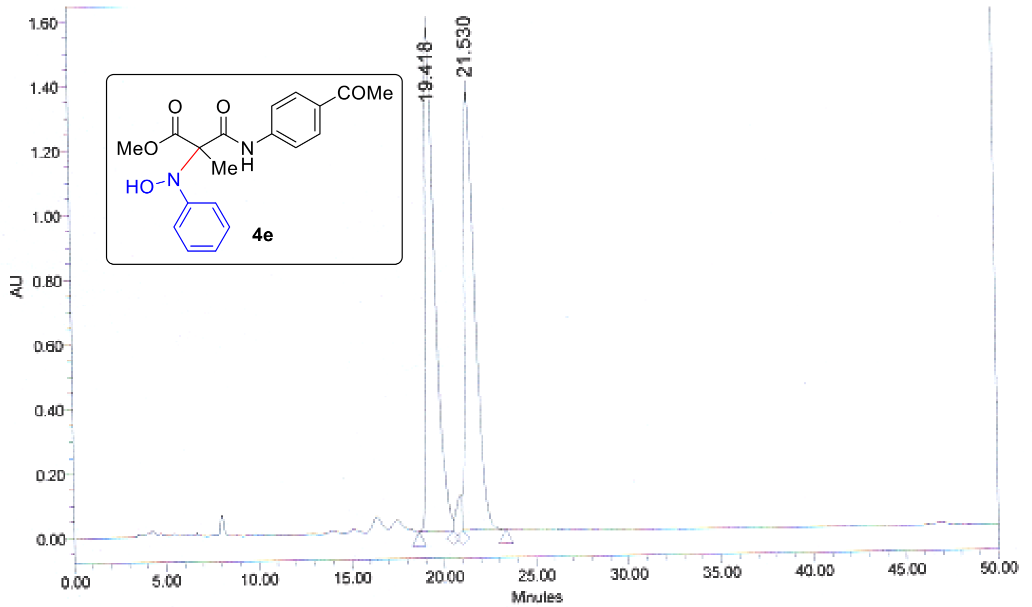

Peak Results

	Name	RT	Area	Height	% Area
1		19.418	56292755	1563268	51.24
2		21.530	53565300	1356806	48.76

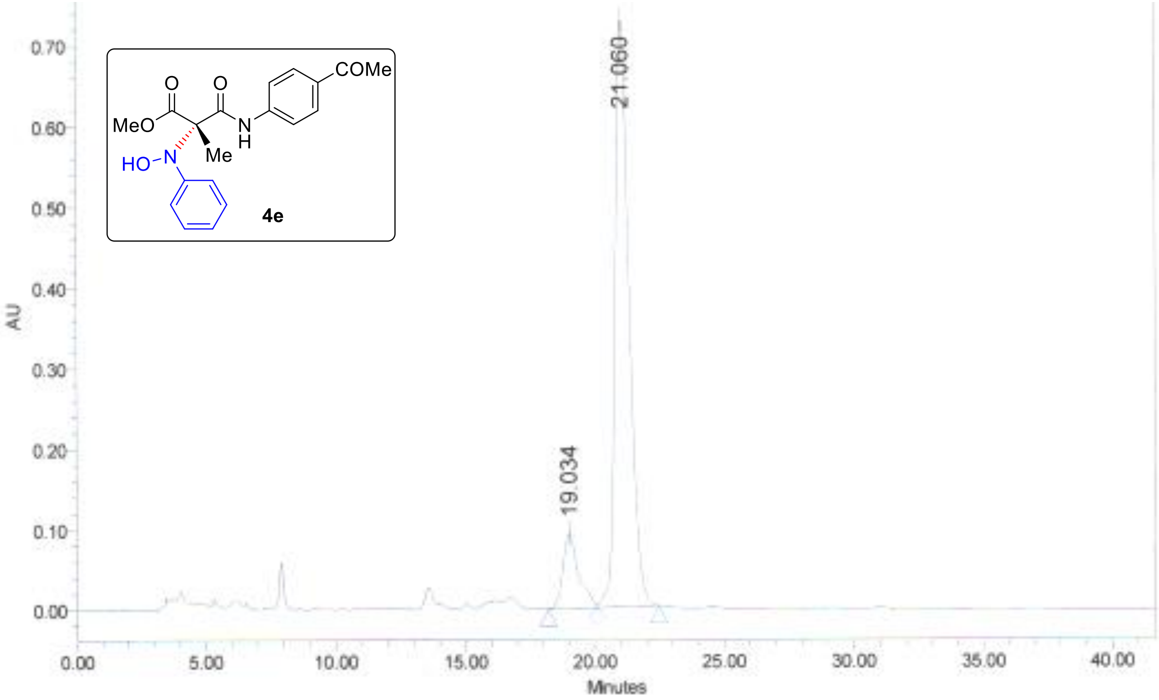

Peak Results

	Name	RT	Area	Height	% Area
1		19.034	3993166	93706	12.64
2		21.060	27595420	729079	87.36

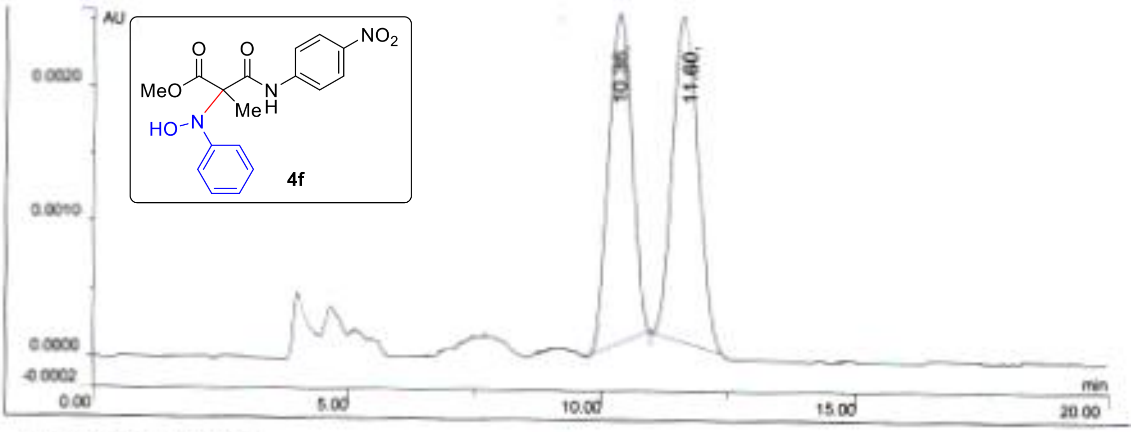

Area % Height %

Sr. No.	Component Name	Ret. Time.	Area µ volt sec	Height µ volt	Area %	Height %	ID	
1		10.36	39617.03	1217	47.63	50.24	U	M
2		11.60	43557.03	1205	52.37	49.76	U	M
			83174.06	2422	100.00	100.00		

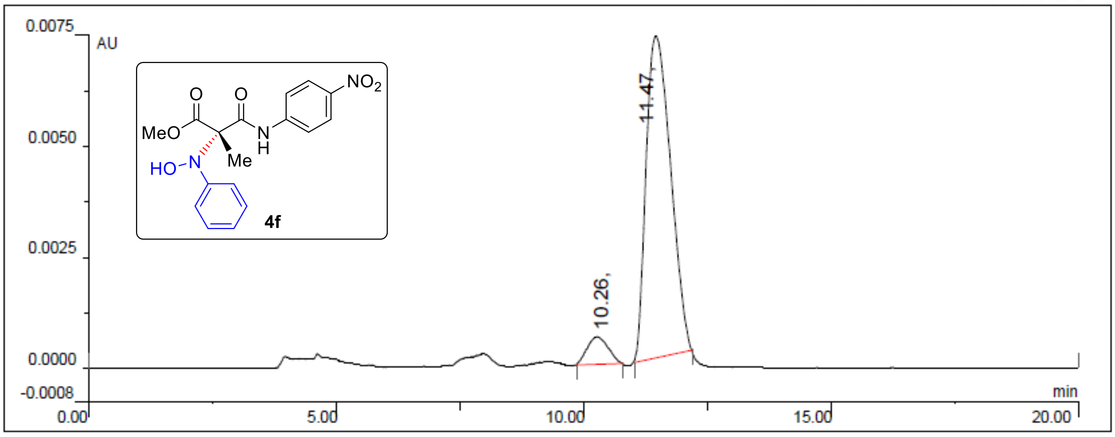

Area % Height %

Sr. No.	Component Name	Ret. Time.	Area µ volt sec	Height µ volt	Area %	Height %	ID	
1		10.26	9392.74	308	6.87	7.80	U	M
2		11.47	127287.94	3645	93.13	92.20	U	M
			136680.69	3954	100.00	100.00		

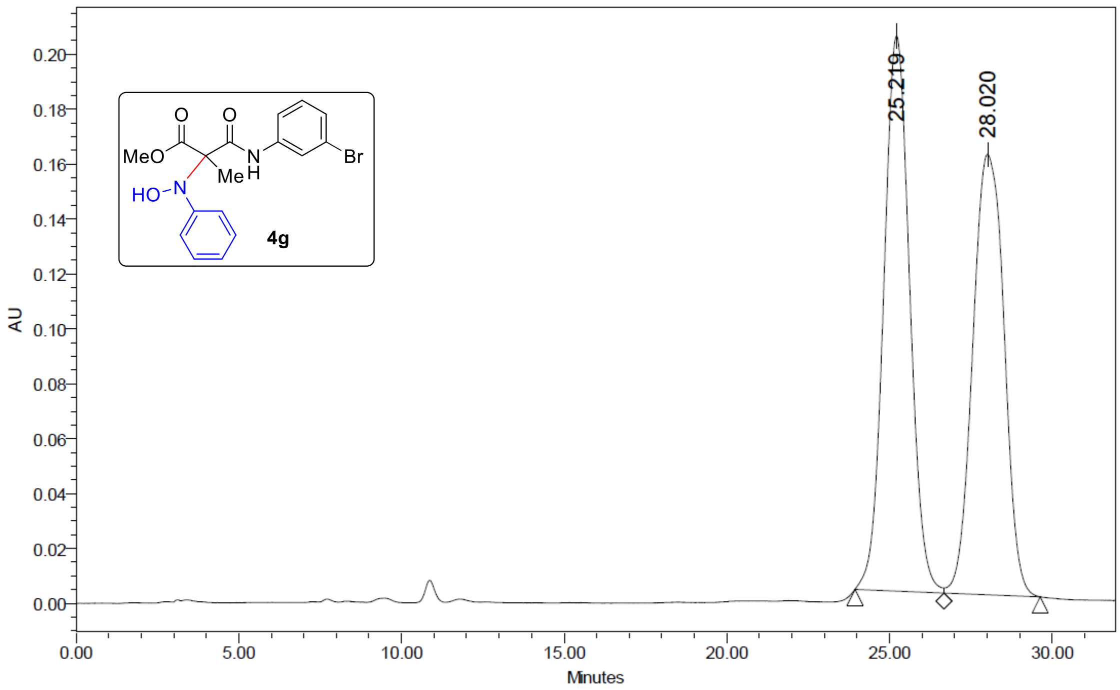

Peak Results

	Name	RT	Area	Height	% Area
1		25.219	11204993	202204	51.05
2		28.020	10745283	160409	48.95

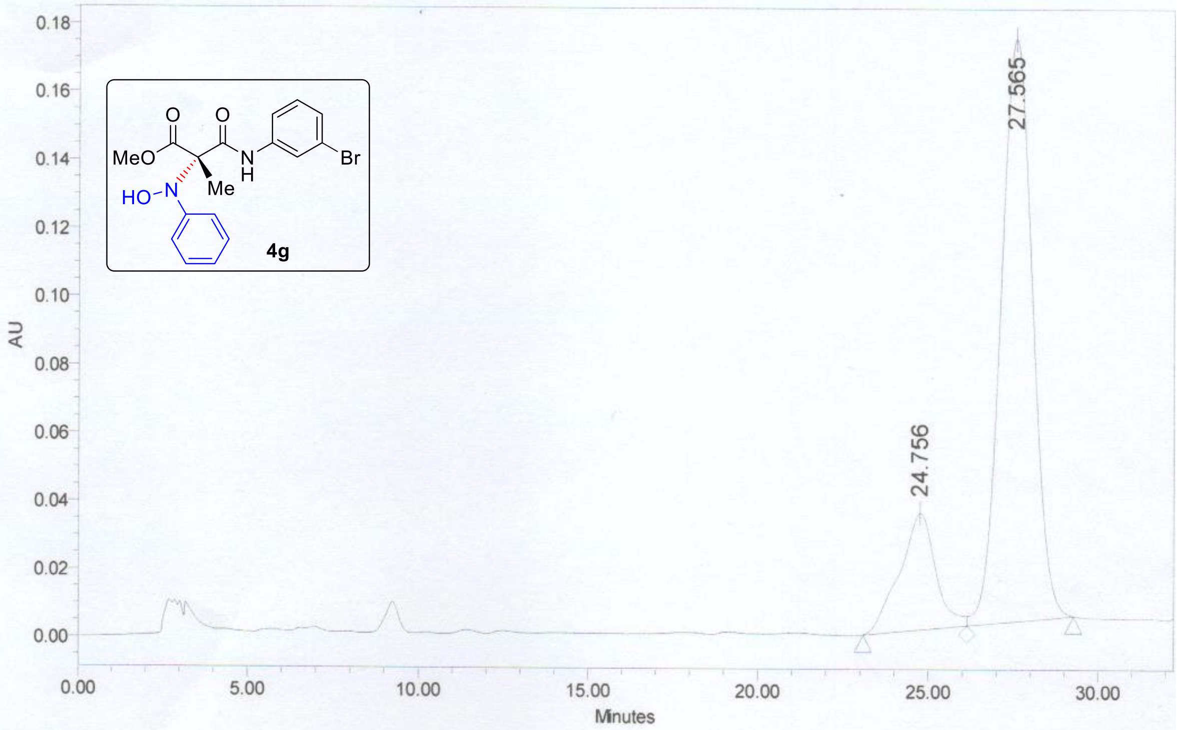

Peak Results

	Name	RT	Area	Height	% Area
1		24.756	2533821	34327	18.48
2		27.565	11180672	170961	81.52

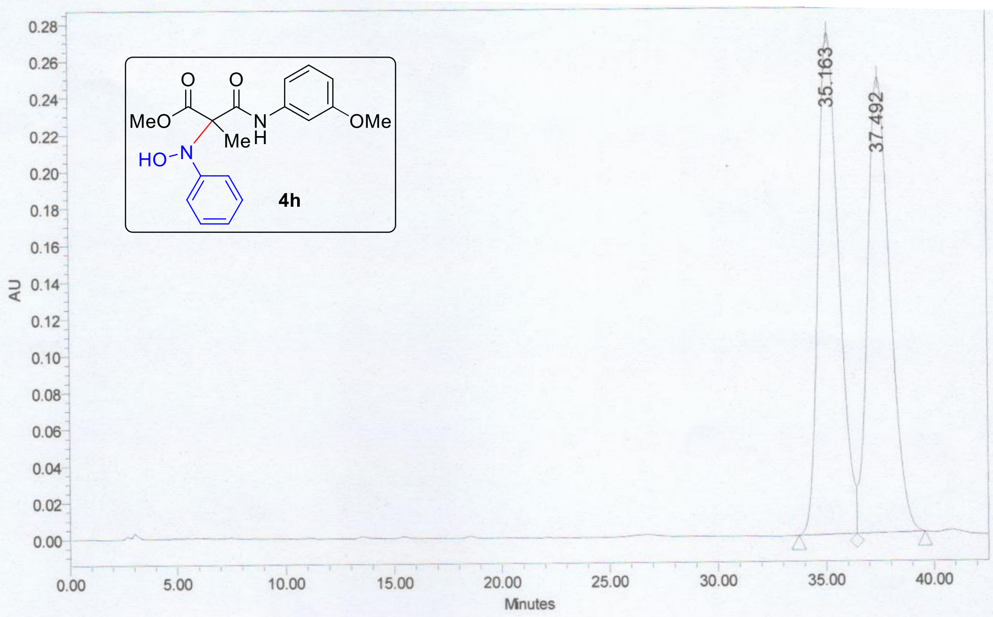

Peak Results

	Name	RT	Area	Height	% Area
1		35.163	18229198	273028	50.31
2		37.492	18002350	247786	49.69

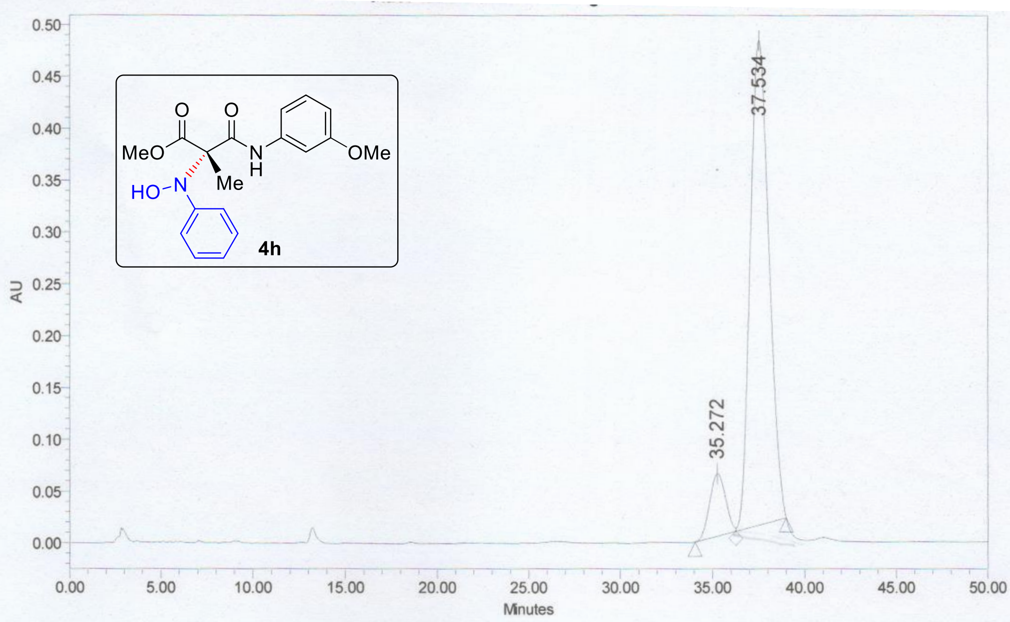

Peak Results

	Name	RT	Area	Height	% Area
1		35.272	3617777	60661	9.86
2		37.534	33074521	467553	90.14

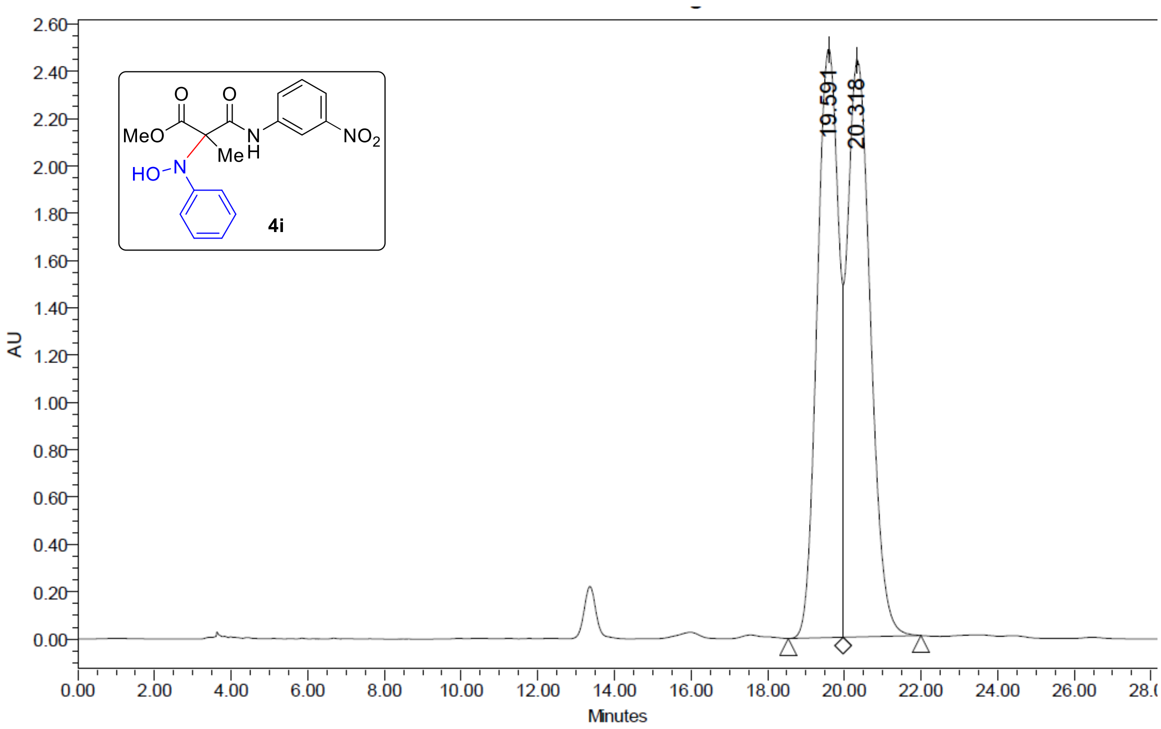

Peak Results

	Name	RT	Area	Height	% Area
1		19.591	97163026	2485510	48.41
2		20.318	103542518	2438182	51.59

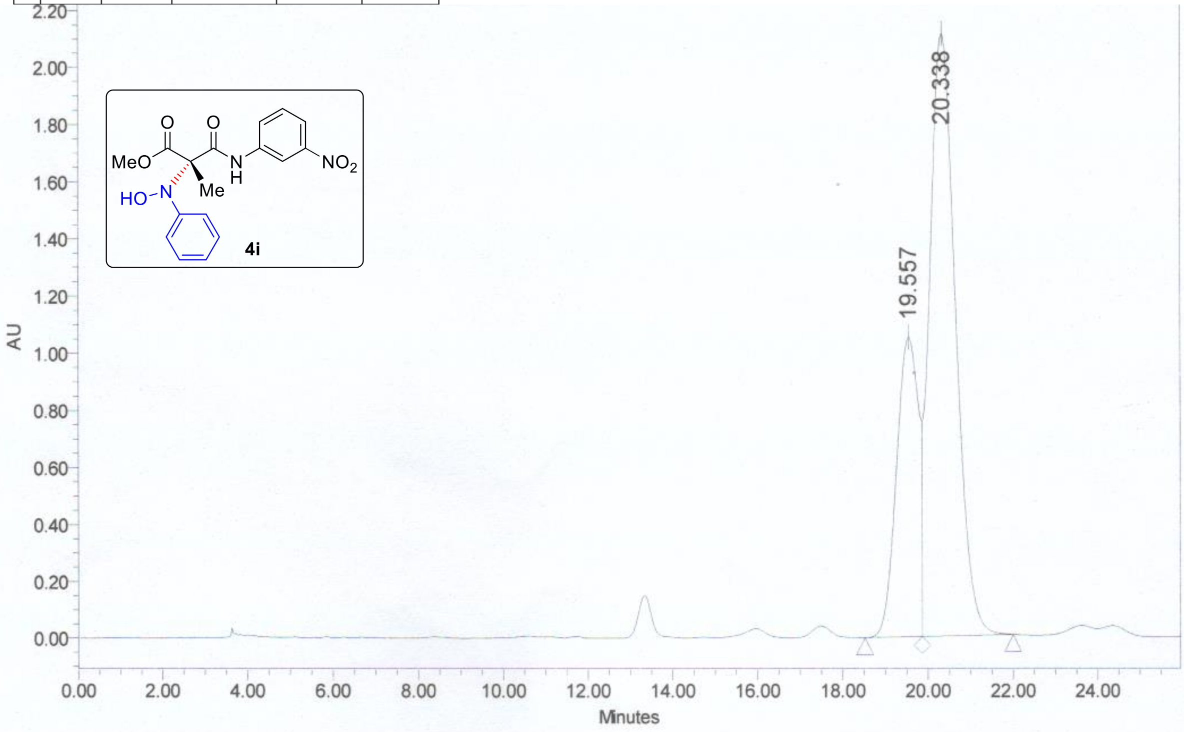

Peak Results

	Name	RT	Area	Height	% Area
1		19.557	37831371	1051532	29.71
2		20.338	89504210	2111206	70.29

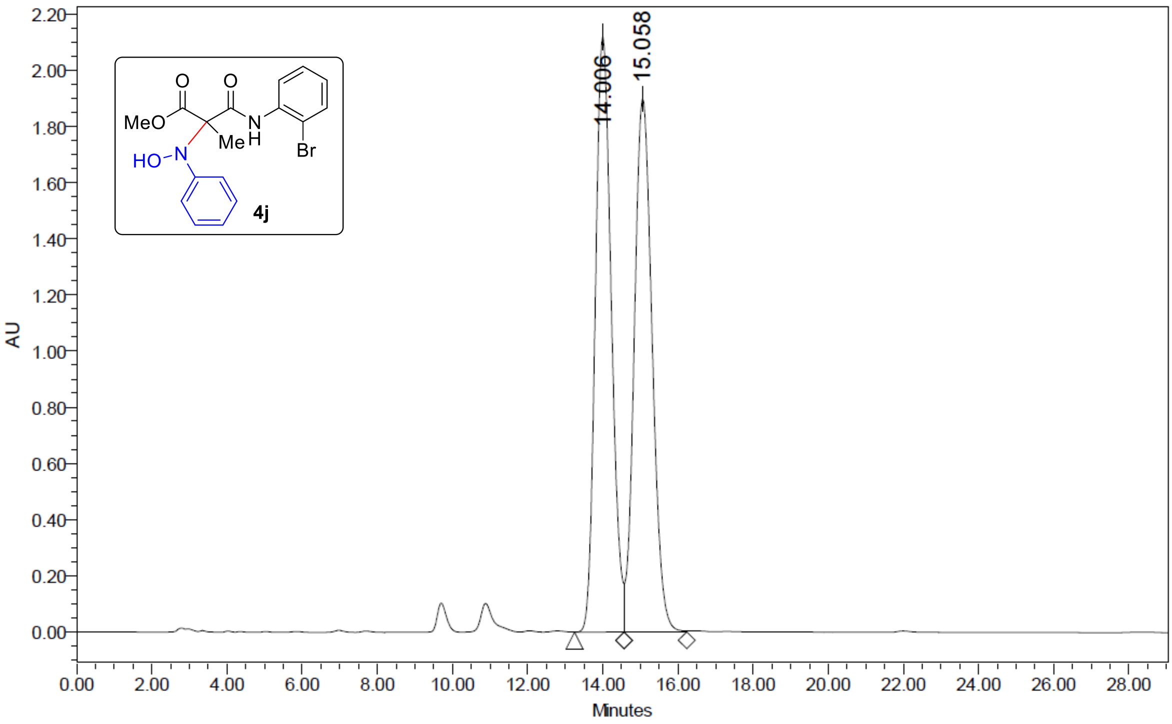

Peak Results

	Name	RT	Area	Height	% Area
1		14.006	62680567	2119760	50.00
2		15.058	62668088	1898139	50.00

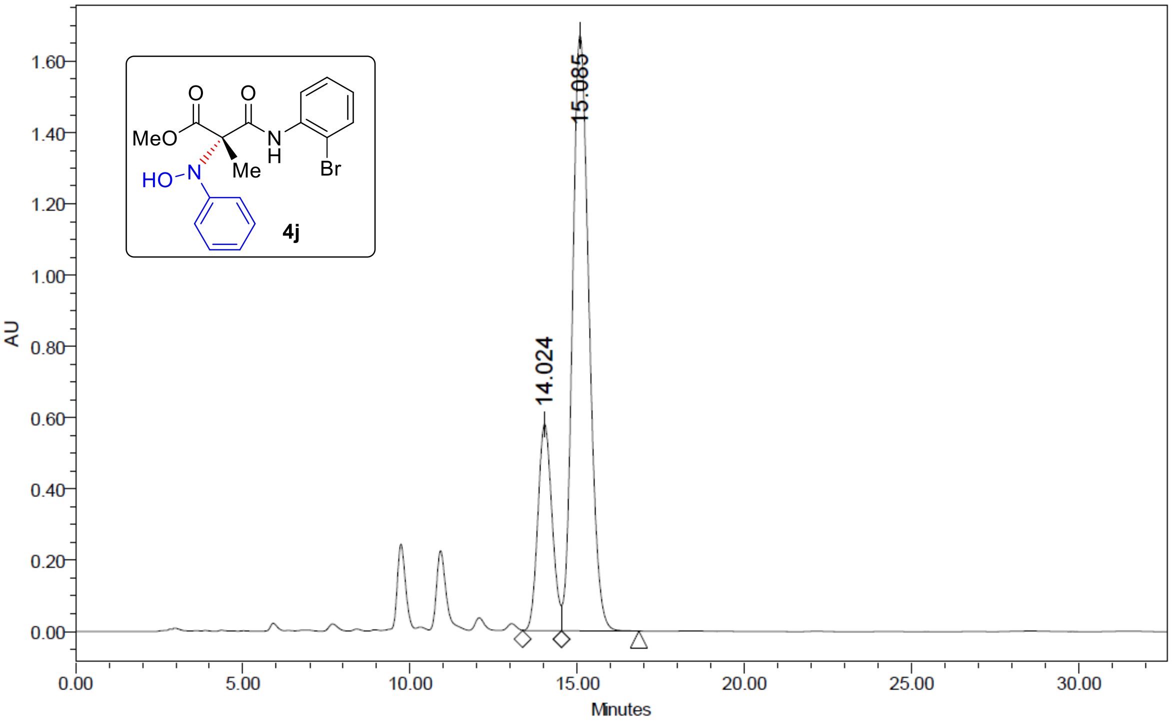

Peak Results

	Name	RT	Area	Height	% Area
1		14.024	17108258	579956	22.98
2		15.085	57354909	1670757	77.02

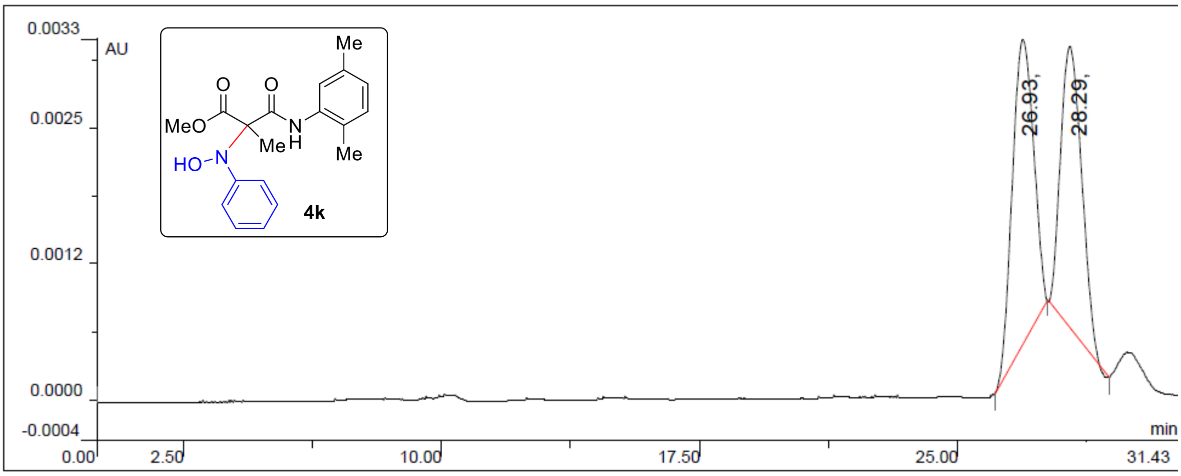

Area % Height %

Sr. No.	Component Name	Ret. Time.	Area μ volt sec	Height μ volt	Area %	Height %	ID	
1		26.93	60463.60	1394	51.31	51.80	U	M
2		28.29	57382.23	1297	48.69	48.20	U	M
			**117845.83**	**2691**	**100.00**	**100.00**		

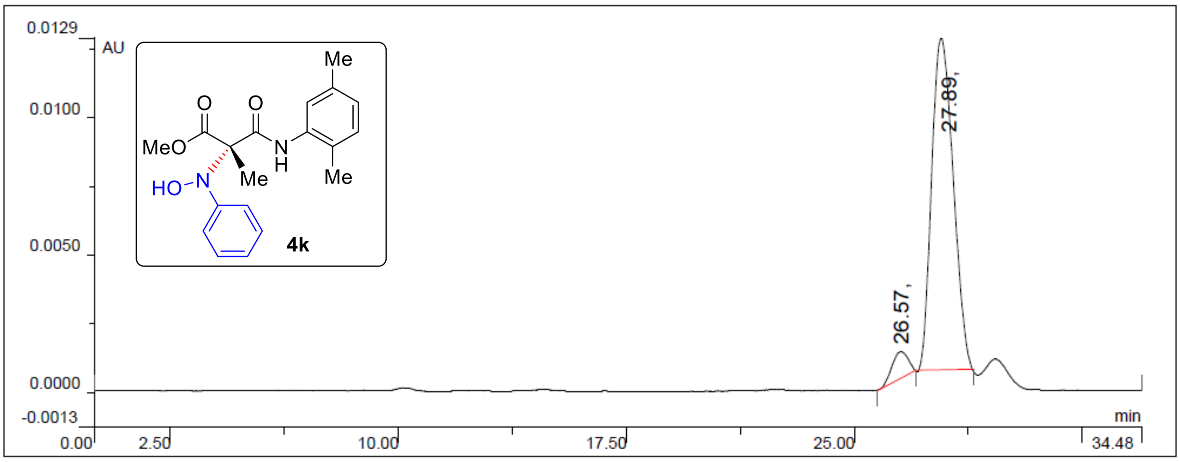

Area % Height %

Sr. No.	Component Name	Ret. Time.	Area μ volt sec	Height μ volt	Area %	Height %	ID	
1		26.57	17428.91	474	5.45	7.27	U	M
2		27.89	302651.03	6045	94.55	92.73	U	M
			**320079.94**	**6520**	**100.00**	**100.00**		

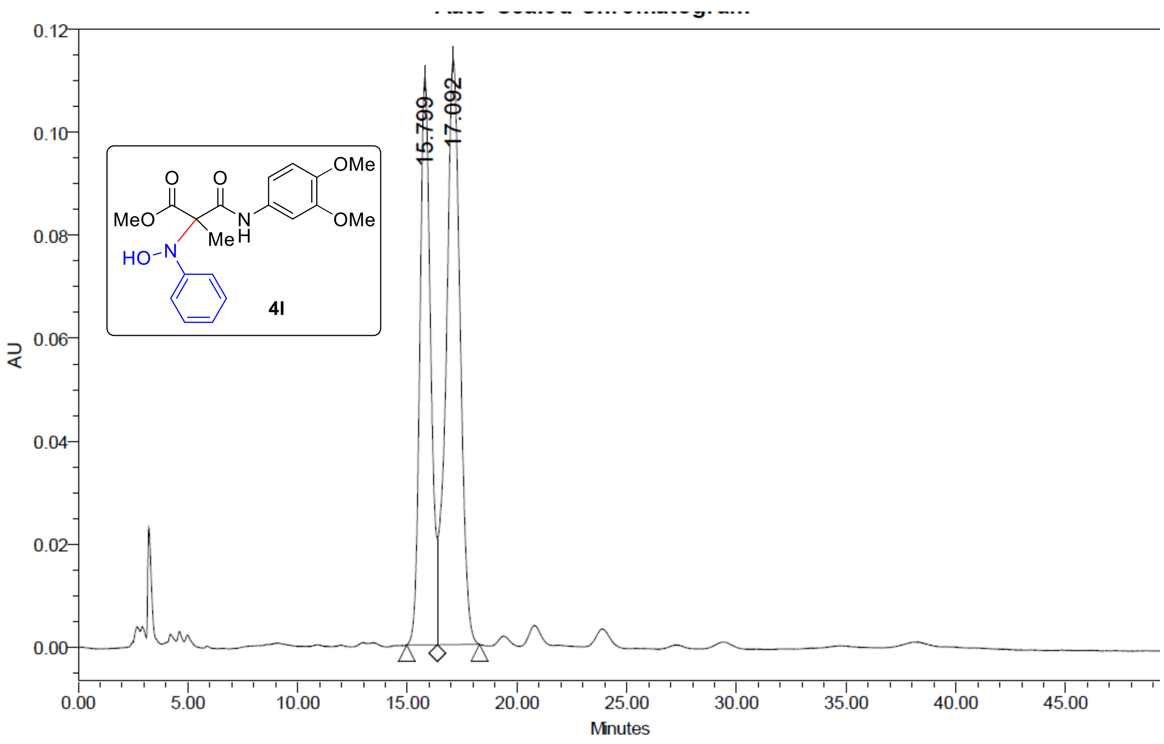

Peak Results

	Name	RT	Area	Height	% Area
1		15.799	3752918	110207	42.66
2		17.092	5043732	113771	57.34

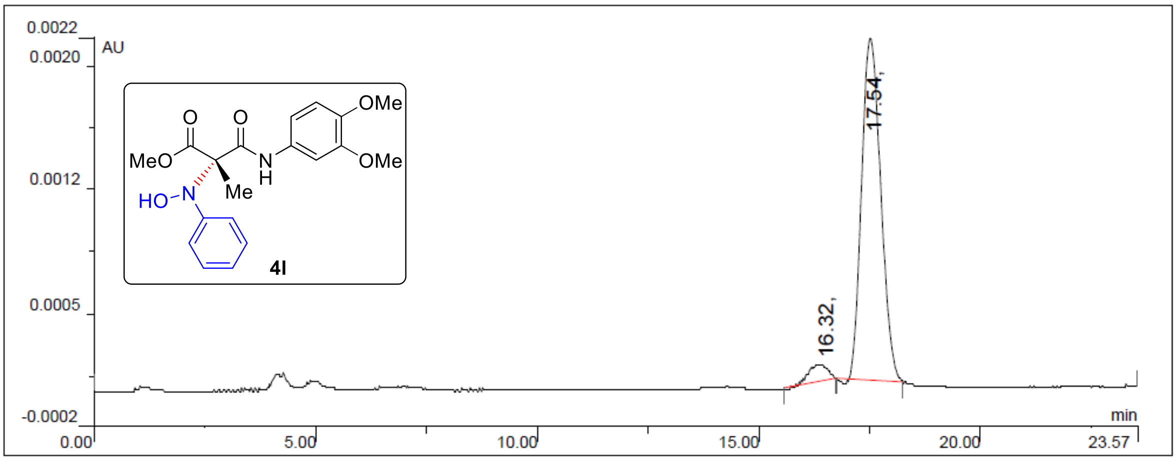

Area % Height %

Sr. No.	Component Name	Ret. Time.	Area μ volt sec	Height μ volt	Area %	Height %	ID	
1		16.32	1559.89	0	4.71	0.00	U	M
2		17.54	31578.29	1034	95.29	100.00	U	M
			**33138.17**	**1034**	**100.00**	**100.00**		

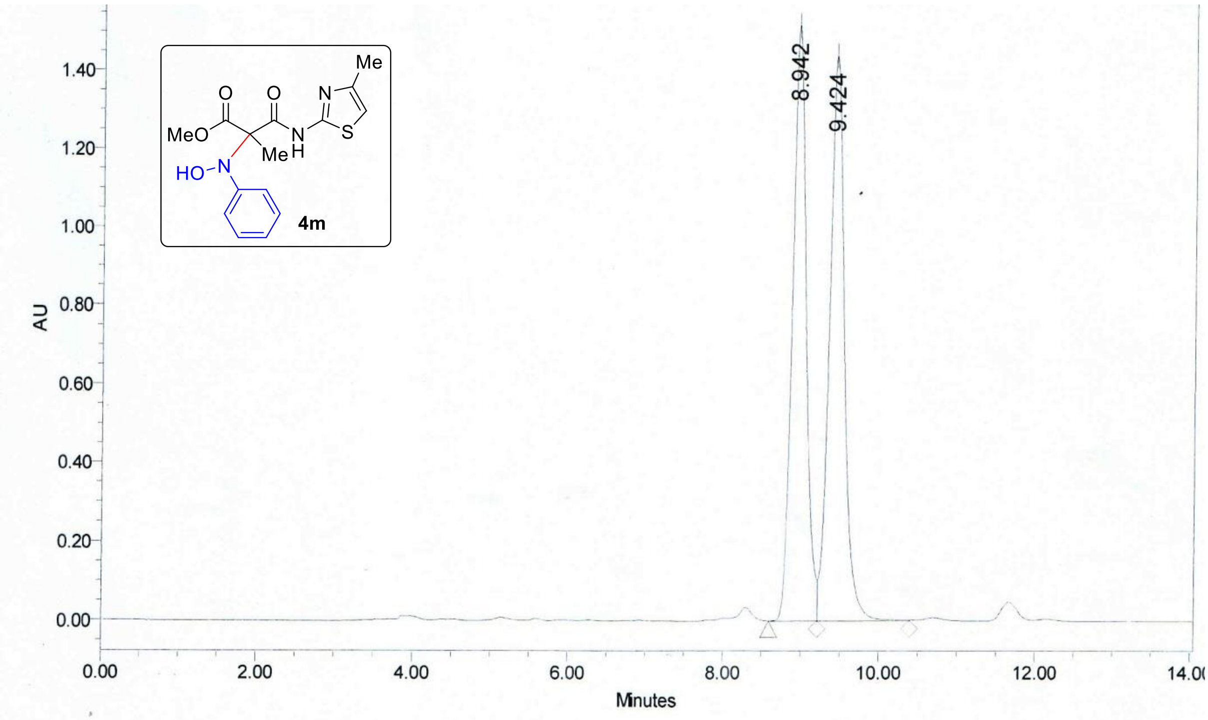

Peak Results

	Name	RT	Area	Height	% Area
1		8.942	18685037	1528419	49.08
2		9.424	19389232	1450639	50.92

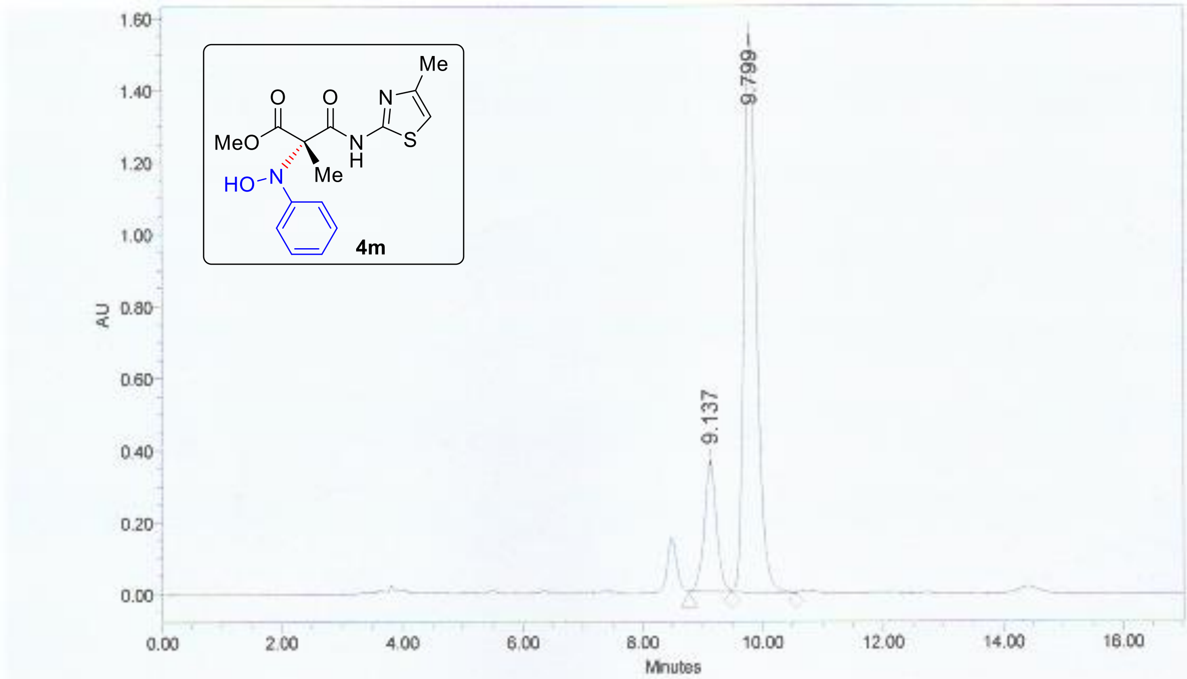

Peak Results

	Name	RT	Area	Height	% Area
1		9.137	5176118	365250	18.85
2		9.799	22285109	1551883	81.15

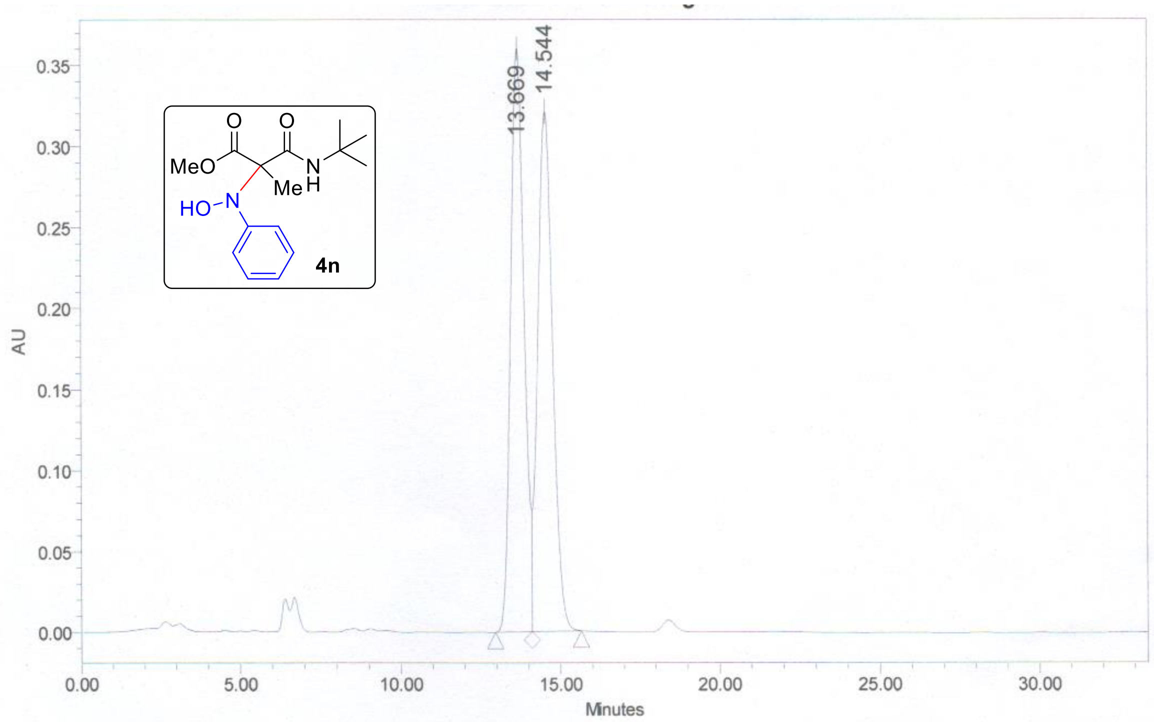

Peak Results

	Name	RT	Area	Height	% Area
1		13.669	10525643	360176	49.99
2		14.544	10529521	321051	50.01

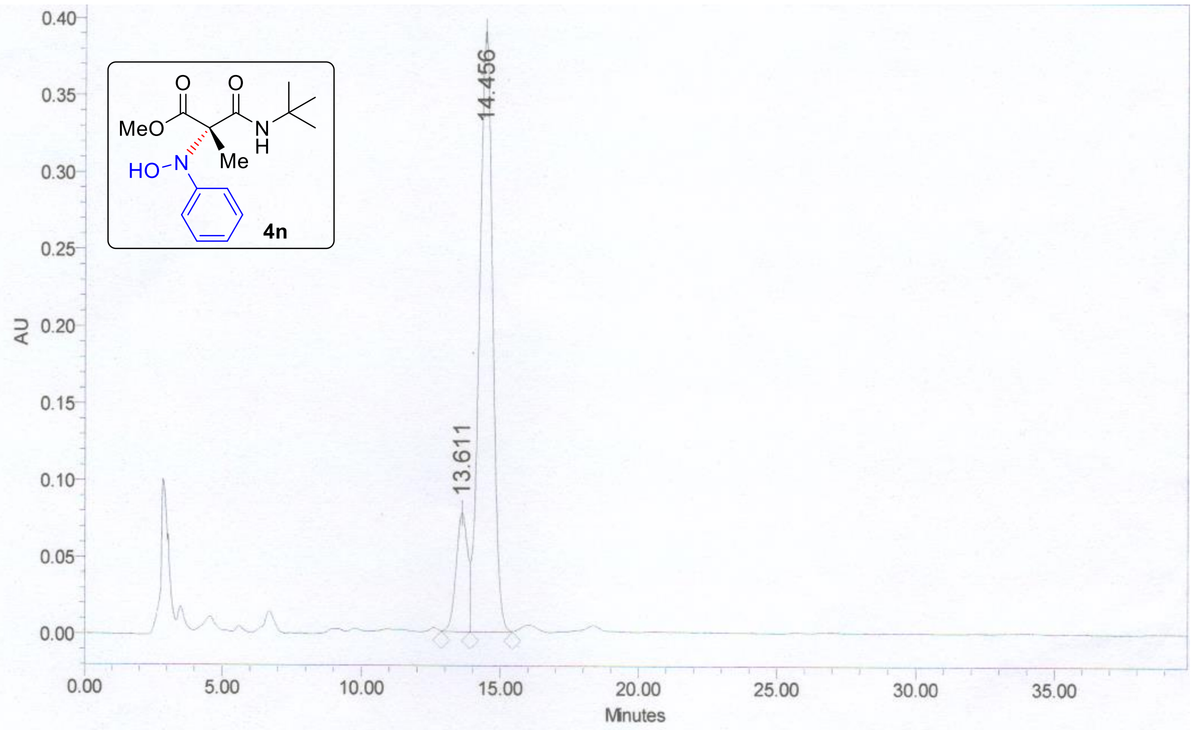

Peak Results

	Name	RT	Area	Height	% Area
1		13.611	2249004	77648	14.66
2		14.456	13092160	390351	85.34

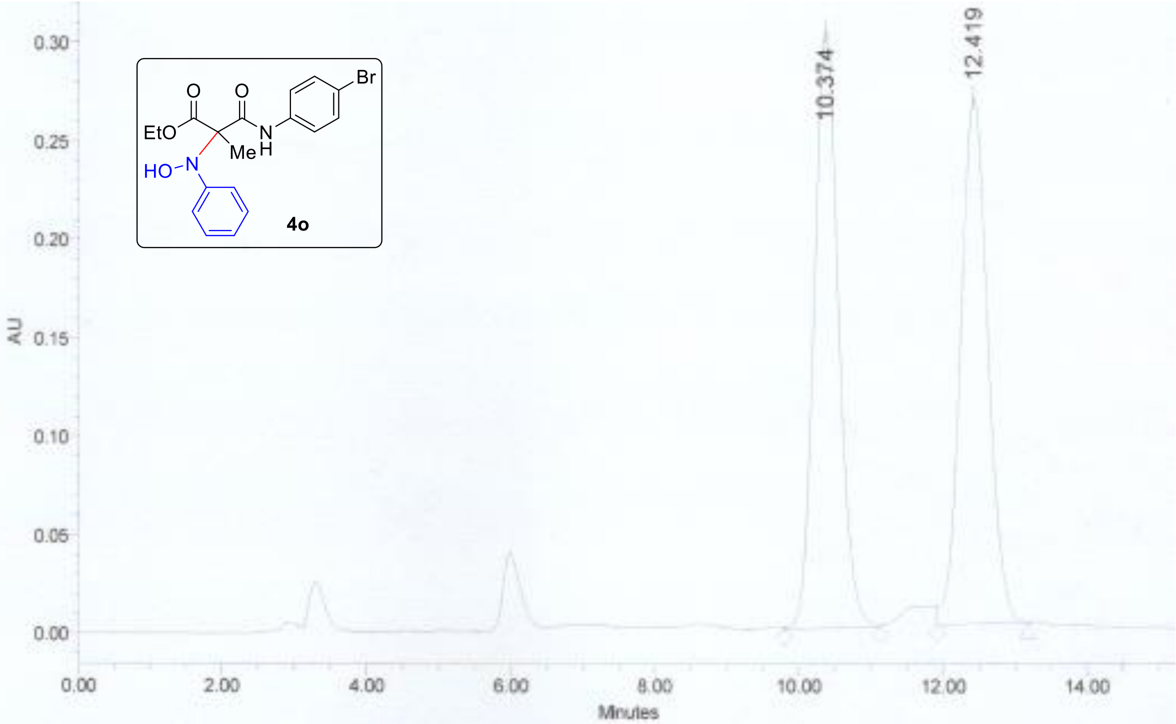

Peak Results

	Name	RT	Area	Height	% Area
1		10.374	7112897	303514	50.45
2		12.419	6986346	257246	49.55

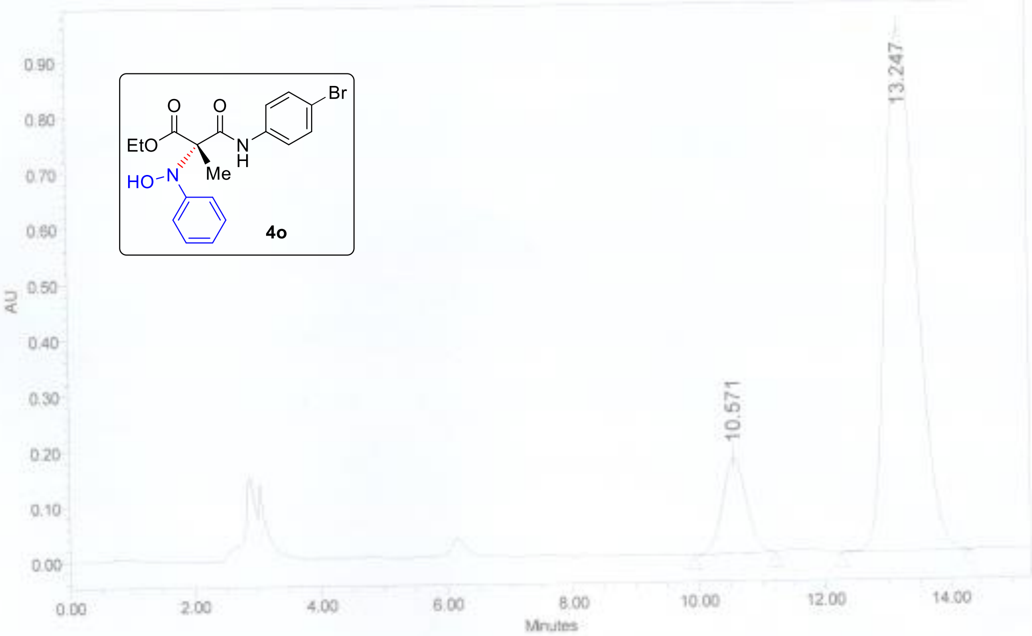

Peak Results

	Name	RT	Area	Height	% Area
1		10.571	4501259	173456	12.18
2		13.247	32462183	943147	87.82

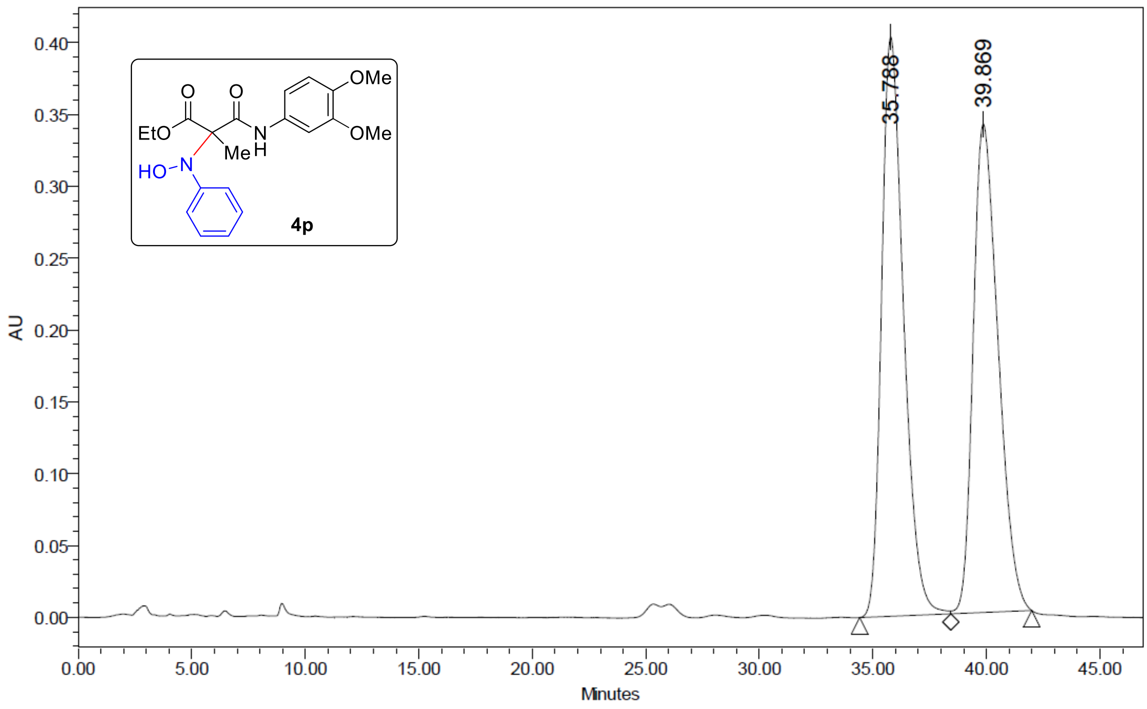

Peak Results

	Name	RT	Area	Height	% Area
1		35.788	27176289	403191	51.10
2		39.869	26007205	339869	48.90

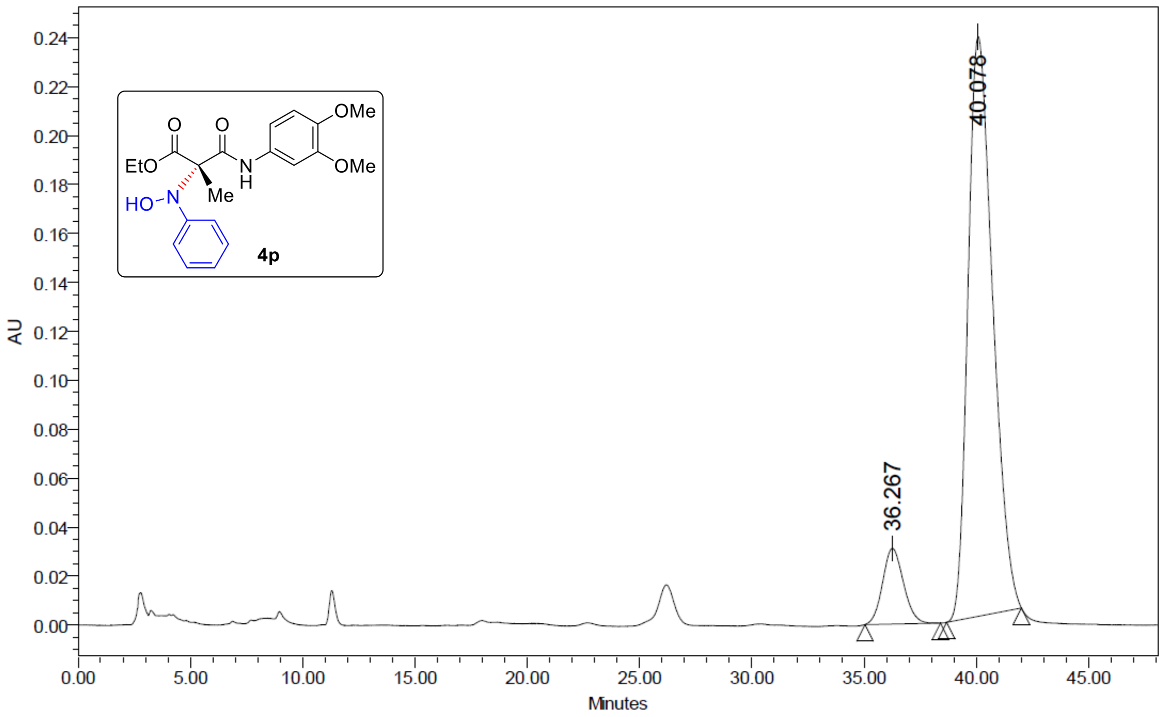

Peak Results

	Name	RT	Area	Height	% Area
1		36.267	2004120	30945	9.95
2		40.078	18135985	236805	90.05

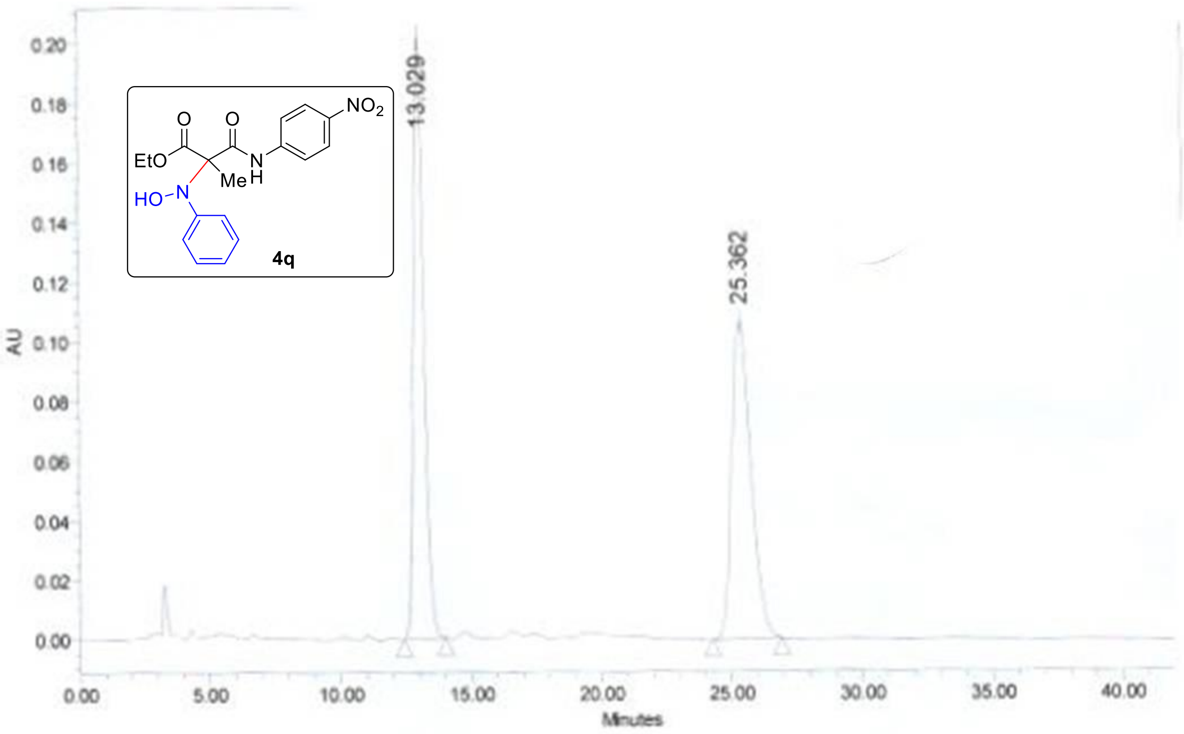

Peak Results

	Name	RT	Area	Height	% Area
1		13.029	5475531	201598	50.23
2		25.362	5426362	106569	49.77

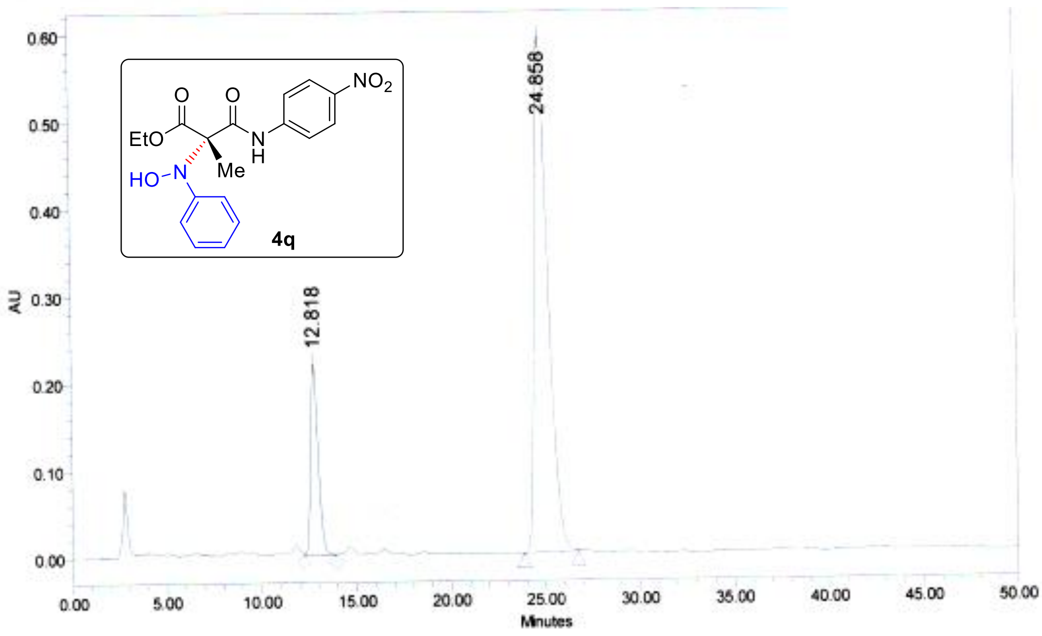

Peak Results

	Name	RT	Area	Height	% Area
1		12.818	6027961	219758	16.73
2		24.858	30013007	592173	83.27

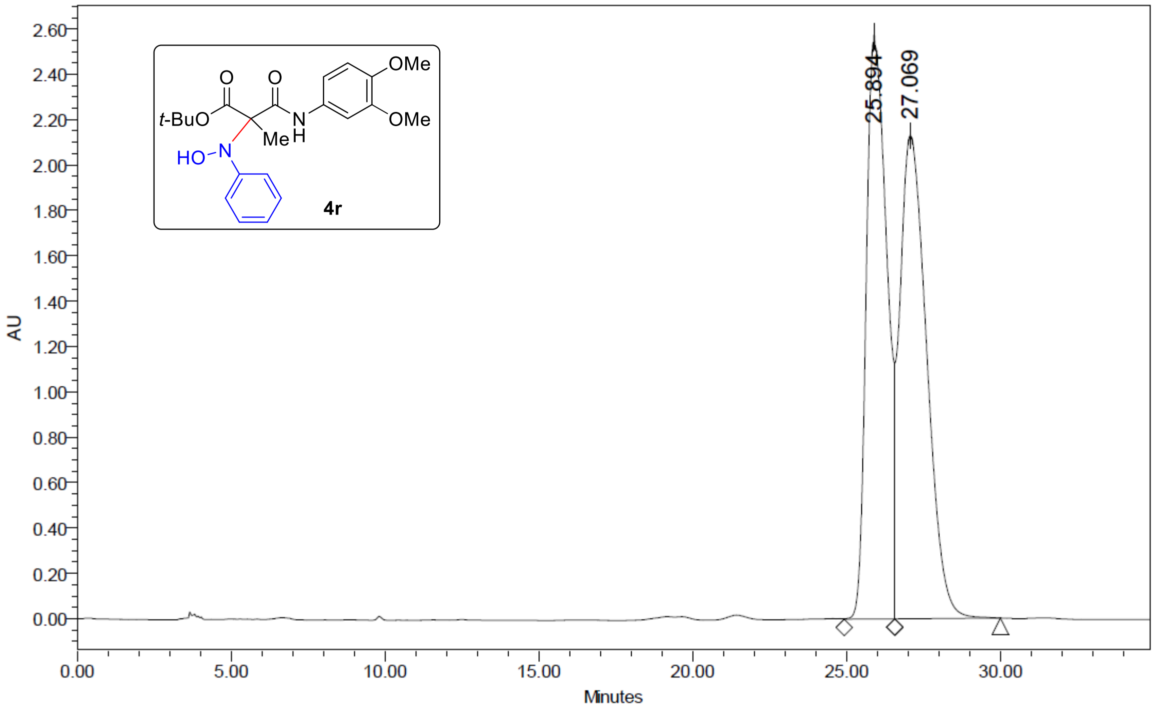

Peak Results

	Name	RT	Area	Height	% Area
1		25.894	119737181	2573806	47.96
2		27.069	129944044	2128938	52.04

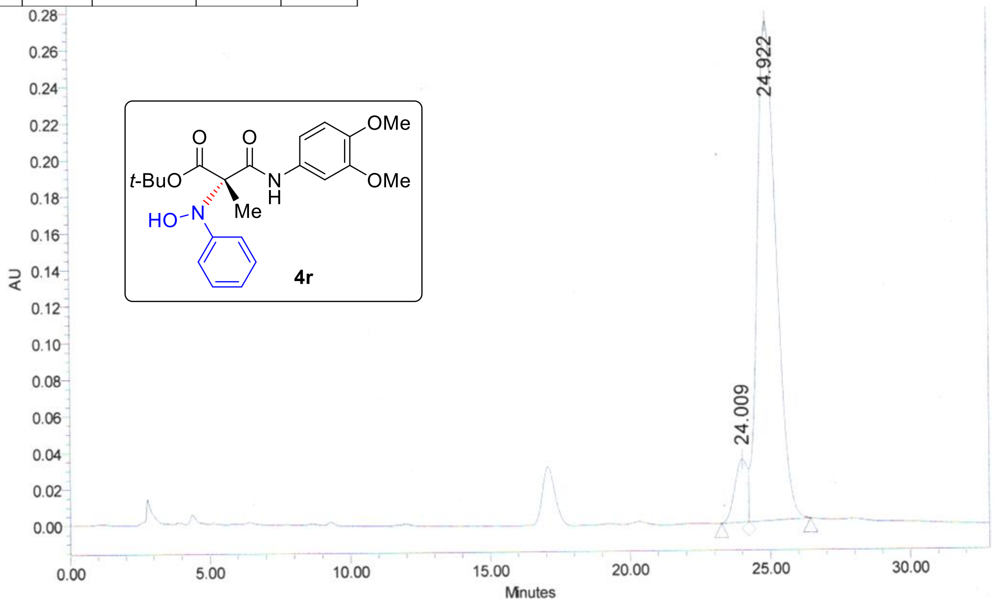

Peak Results

	Name	RT	Area	Height	% Area
1		24.009	1080063	34707	7.77
2		24.922	12817488	273358	92.23

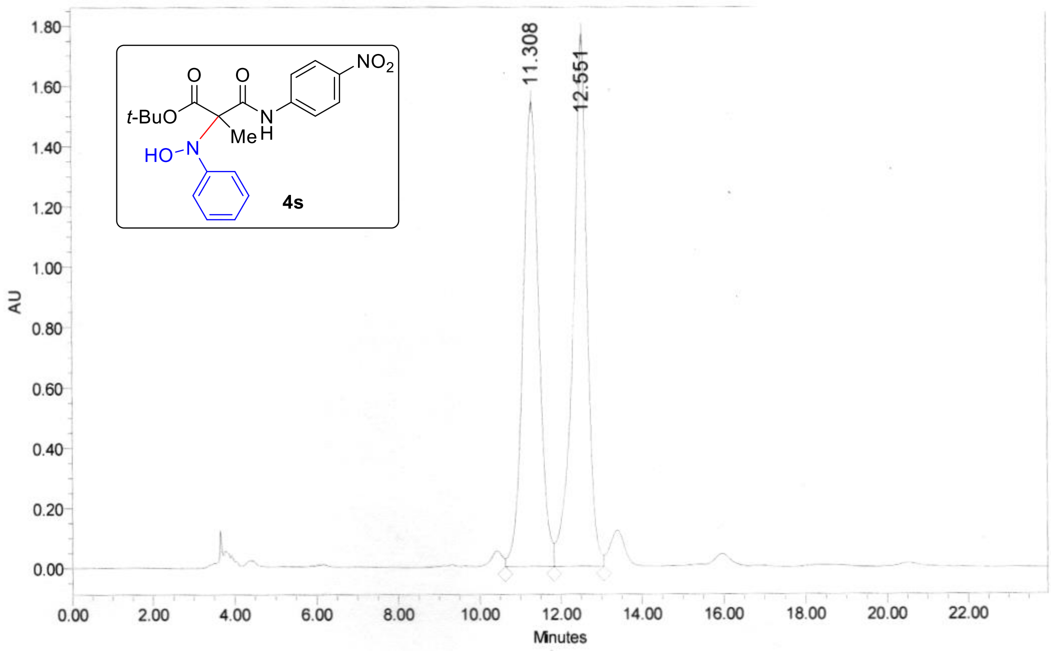

Peak Results

	Name	RT	Area	Height	% Area
1		11.308	41880293	1541295	49.57
2		12.551	42608916	1767799	50.43

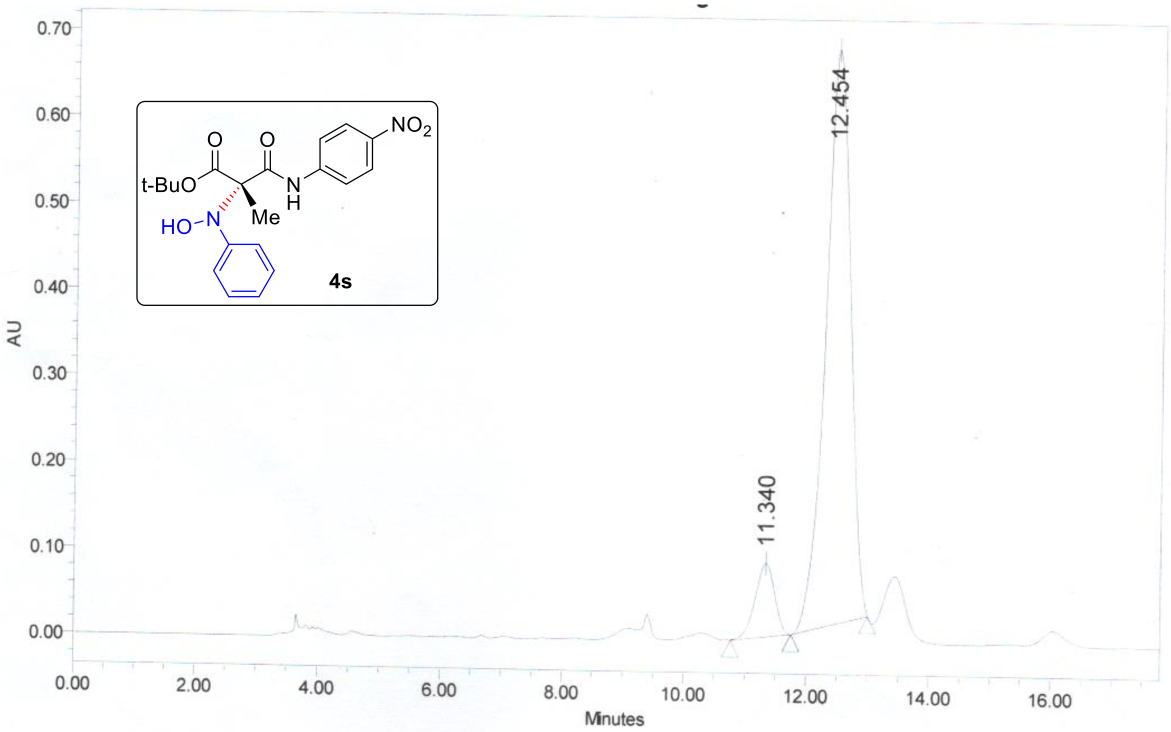

Peak Results

	Name	RT	Area	Height	% Area
1		11.340	1876471	85359	9.20
2		12.454	18519793	664953	90.80

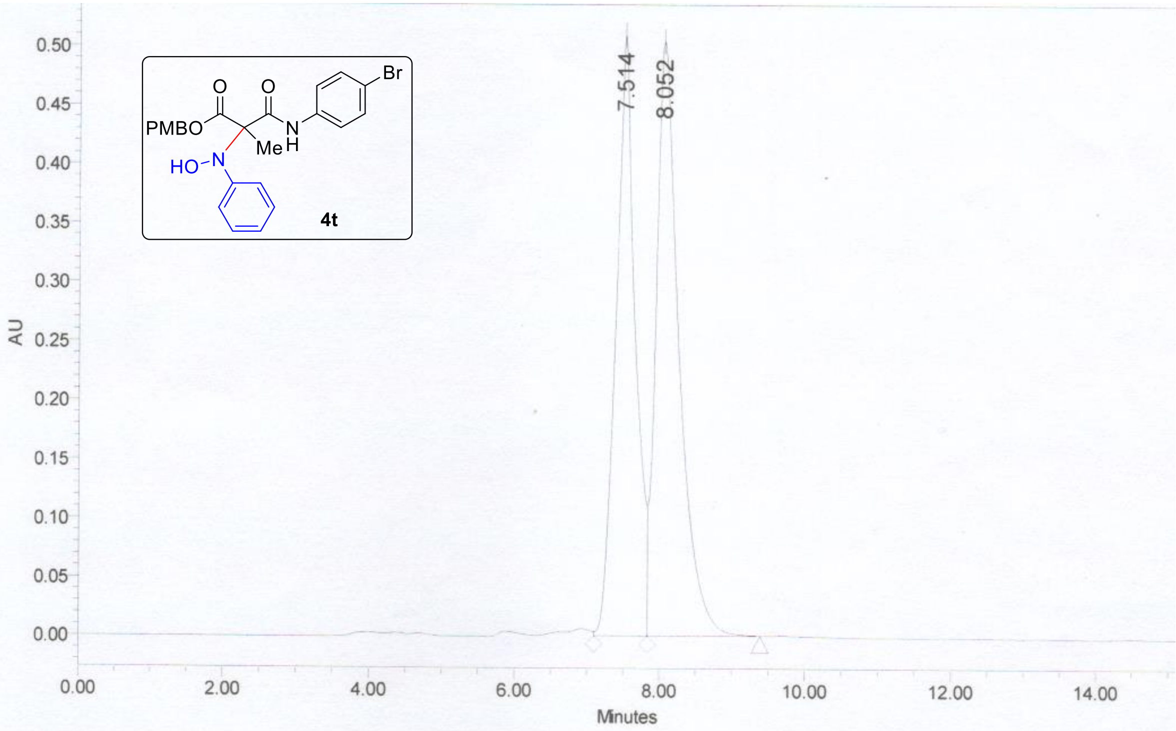

Peak Results

	Name	RT	Area	Height	% Area
1		7.514	9509216	509883	46.03
2		8.052	11147398	504245	53.97

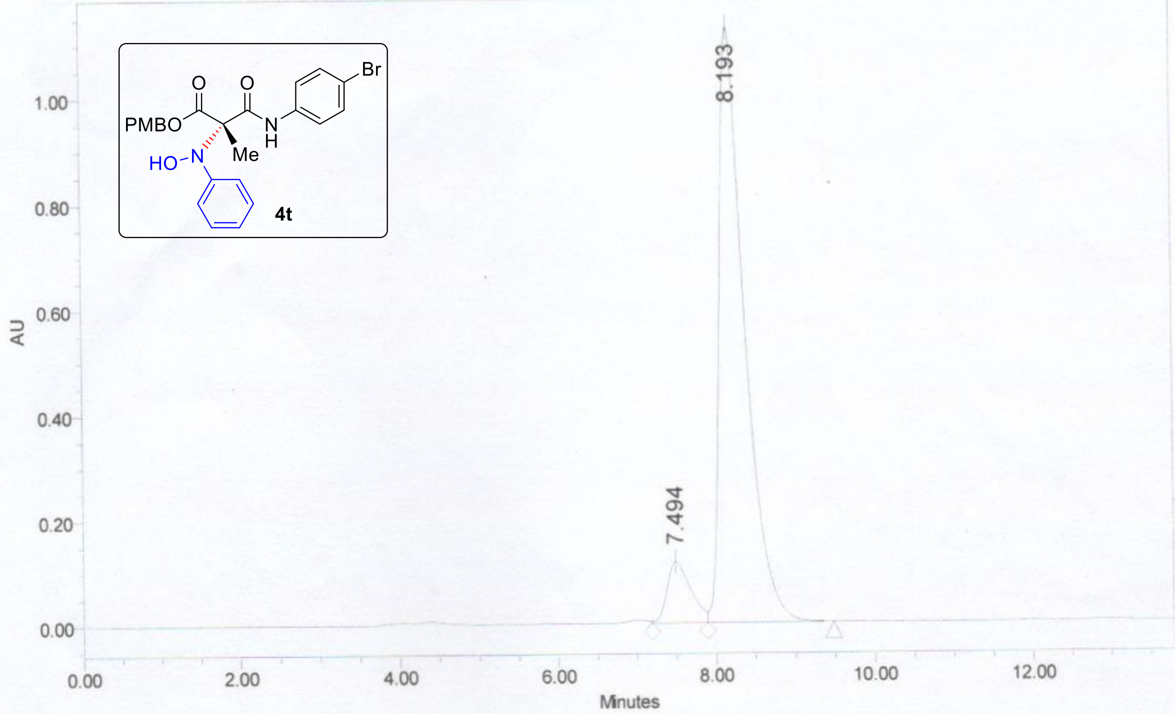

Peak Results

	Name	RT	Area	Height	% Area
1		7.494	2425287	116503	8.86
2		8.193	24937220	1128942	91.14

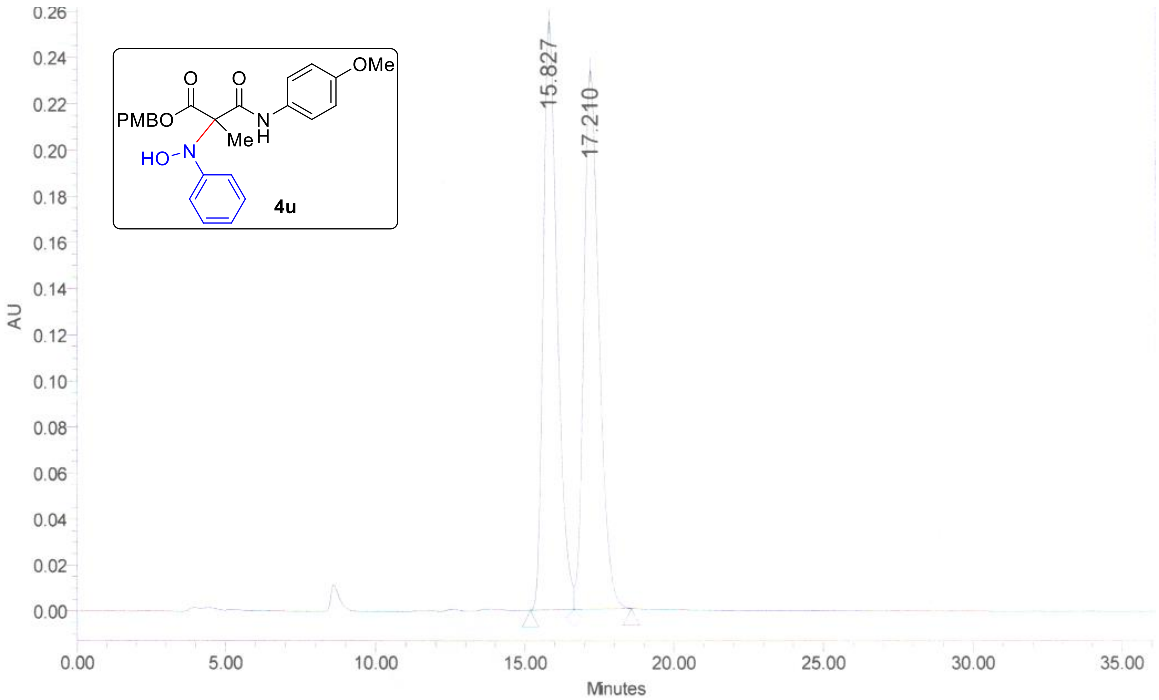

Peak Results

	Name	RT	Area	Height	% Area
1		15.827	8366672	255386	50.15
2		17.210	8317556	233791	49.85

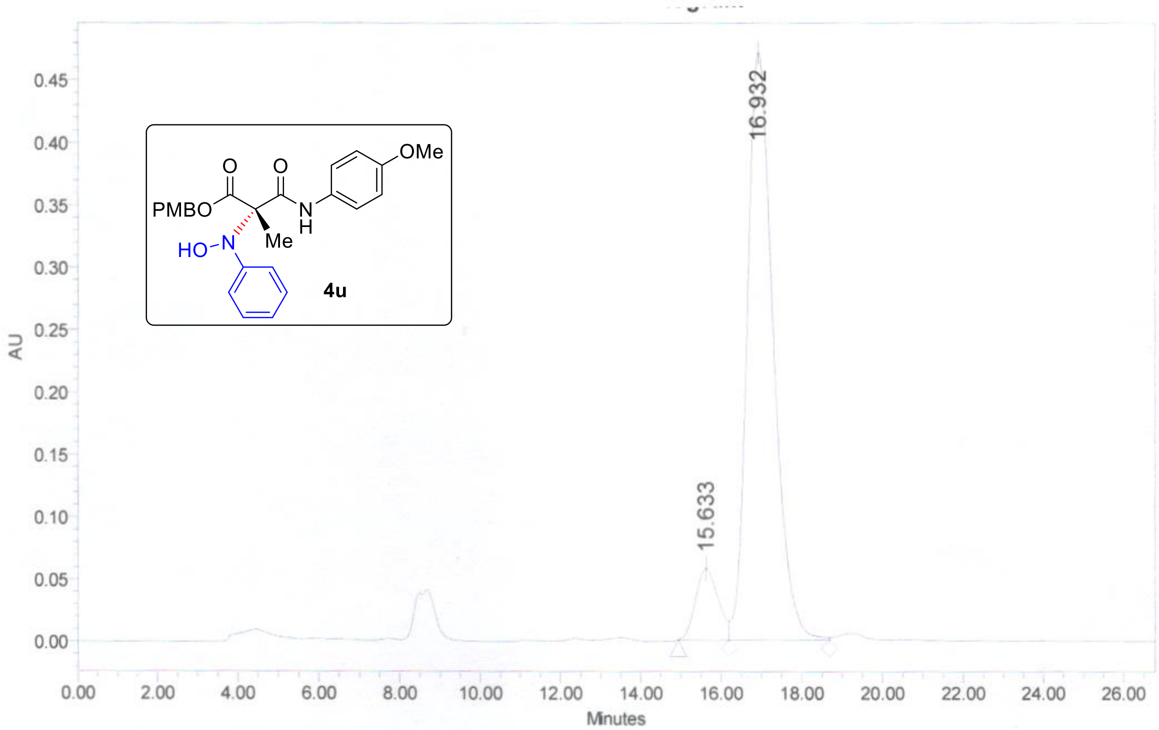

Peak Results

	Name	RT	Area	Height	% Area
1		15.633	2248592	56936	9.67
2		16.932	21013727	470780	90.33

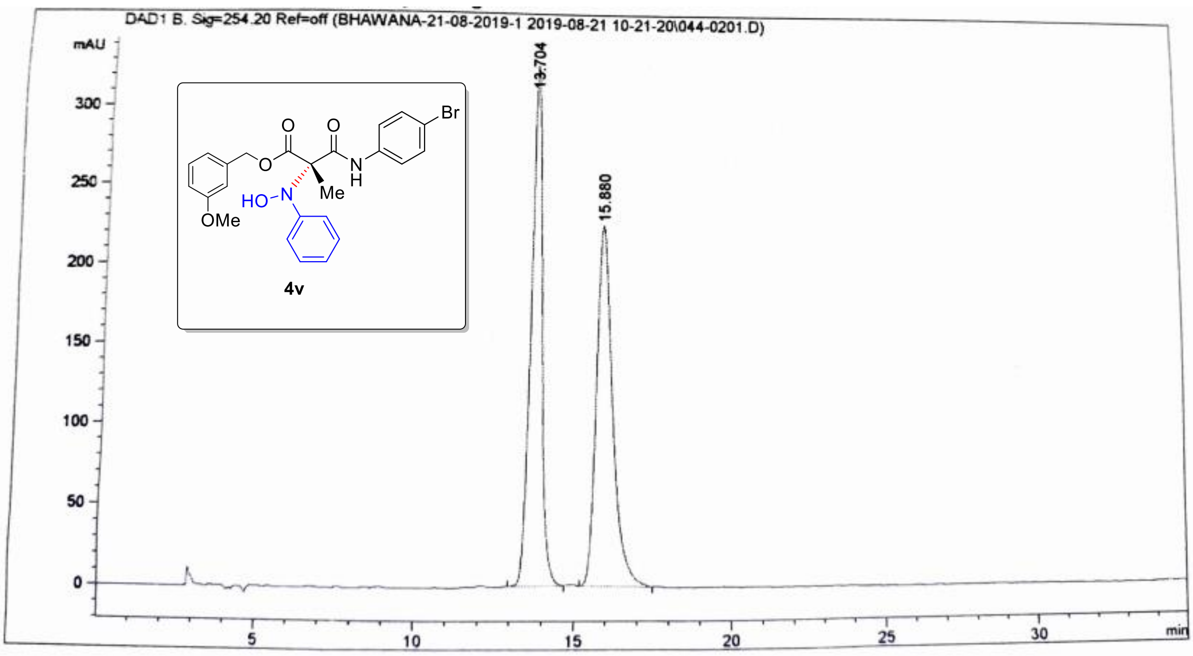

Peak #	RetTime [min]	Type	Width [min]	Area [mAU*s]	Height [mAU]	Area %
1	13.704	BB	0.3678	7876.48096	326.14255	49.9212
2	15.880	BB	0.5310	7901.35156	223.20715	50.0788

Totals : 1.57778e4 549.34970

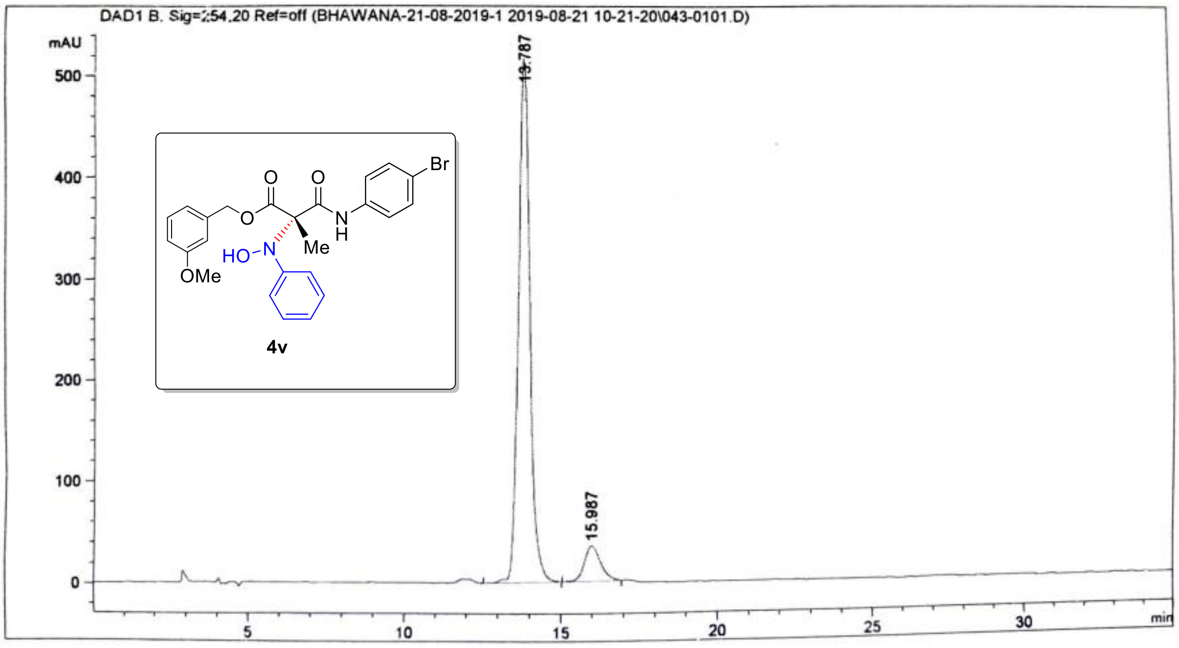

Peak #	RetTime [min]	Type	Width [min]	Area [mAU*s]	Height [mAU]	Area %
1	13.787	BB	0.3780	1.27783e4	514.14838	91.3068
2	15.987	BB	0.5348	1216.60291	34.38516	8.6932

Totals : 1.39949e4 548.53354

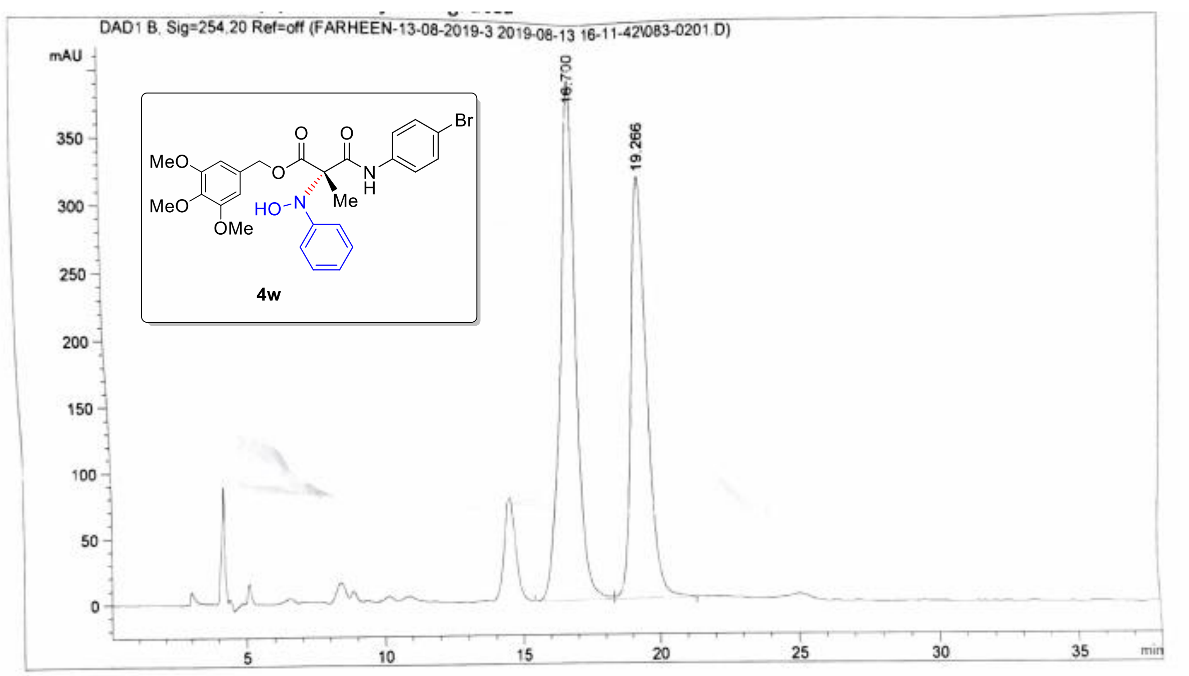

Peak #	RetTime [min]	Type	Width [min]	Area [mAU*s]	Height [mAU]	Area %
1	16.700	BV	0.5911	1.56196e4	396.91266	54.9978
2	19.266	VB	0.6136	1.27808e4	320.27298	45.0022
Totals :				2.84004e4	717.18564	

Additional Info : Peak(s) manually integrated

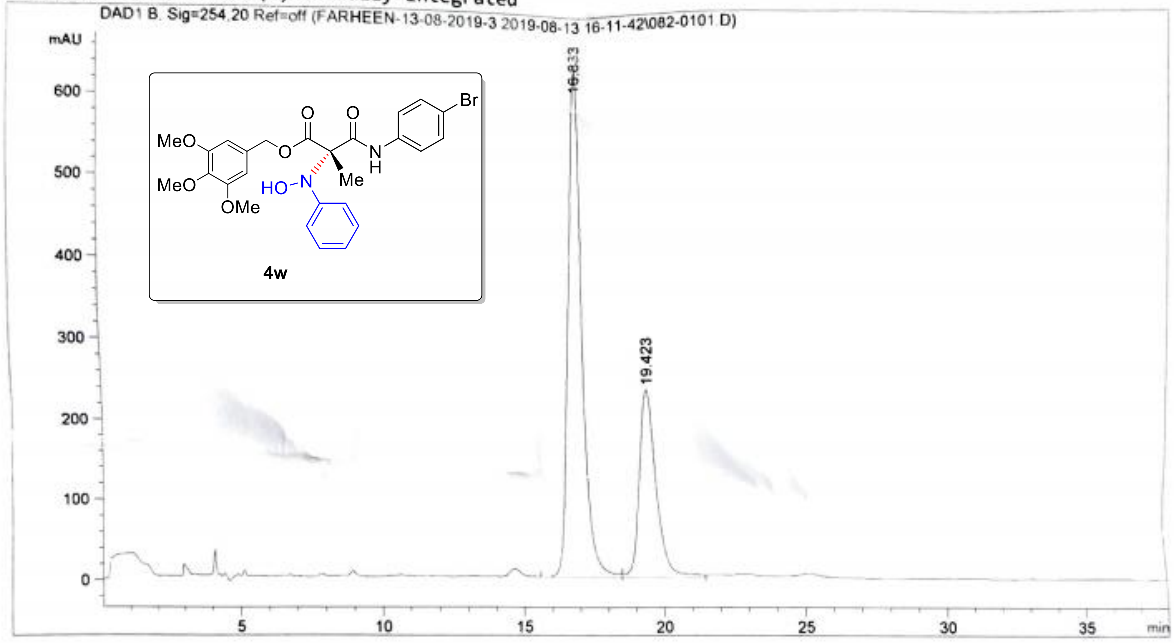

Peak #	RetTime [min]	Type	Width [min]	Area [mAU*s]	Height [mAU]	Area %
1	16.833	BV	0.5301	2.21835e4	637.23199	69.3520
2	19.423	VV	0.6299	9803.32813	235.38416	30.6480
Totals :				3.19869e4	872.61615	

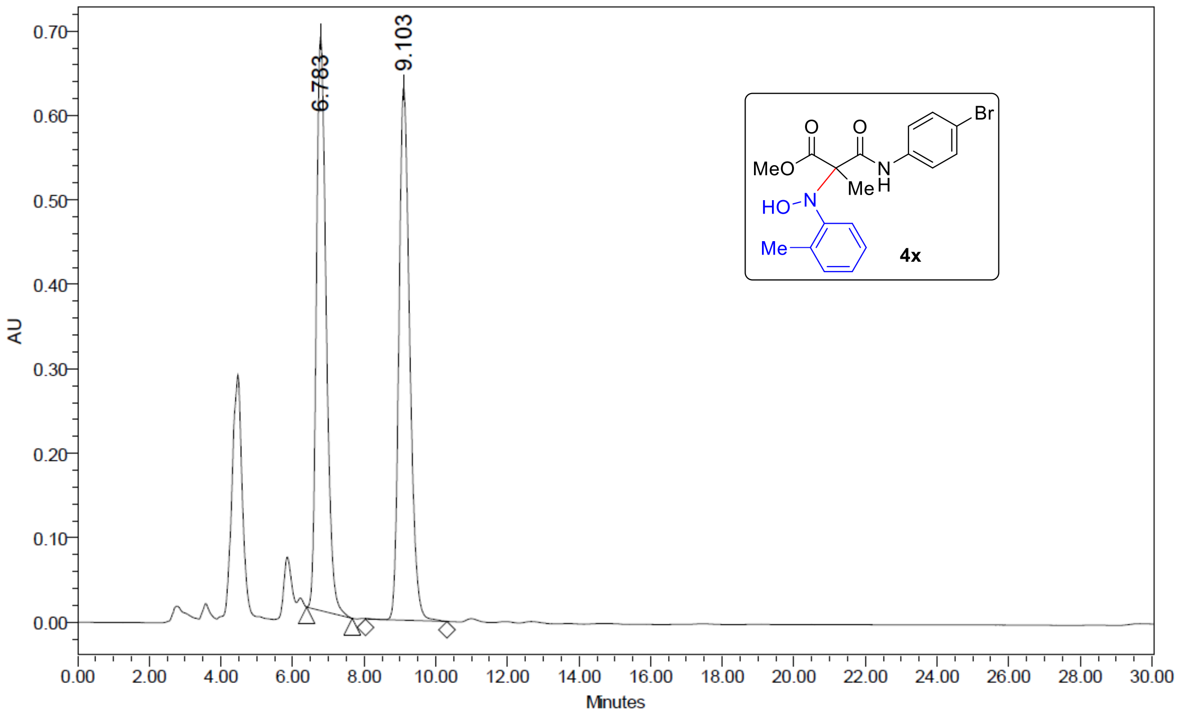

Peak Results

	Name	RT	Area	Height	% Area
1		6.783	12985981	679709	49.50
2		9.103	13249321	630724	50.50

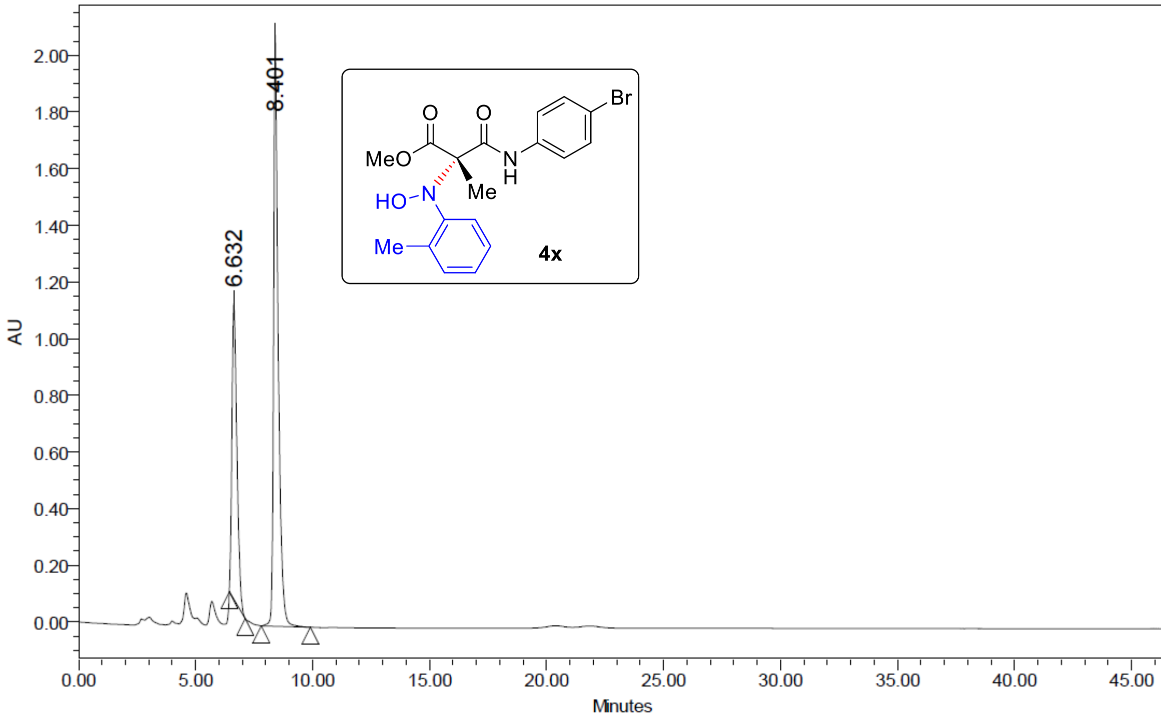

Peak Results

	Name	RT	Area	Height	% Area
1		6.632	15350541	1044740	34.84
2		8.401	28715872	2086879	65.16

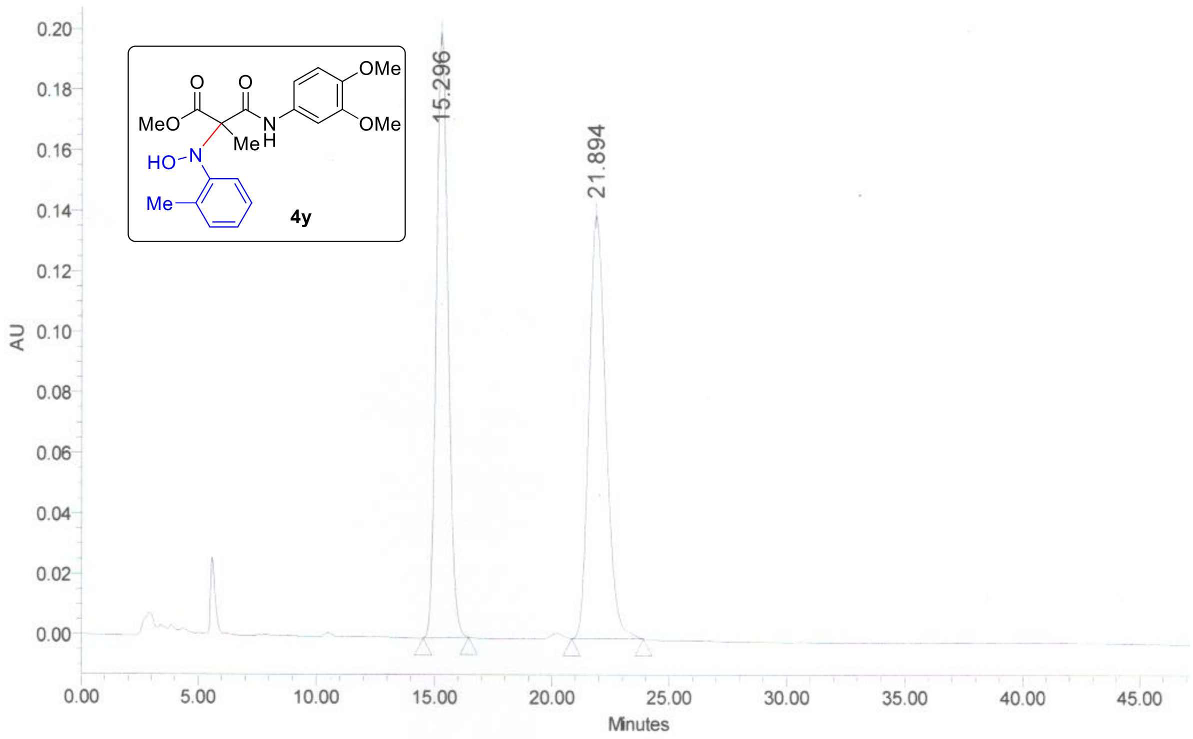

Peak Results

	Name	RT	Area	Height	% Area
1		15.296	6957461	199991	49.73
2		21.894	7033601	139914	50.27

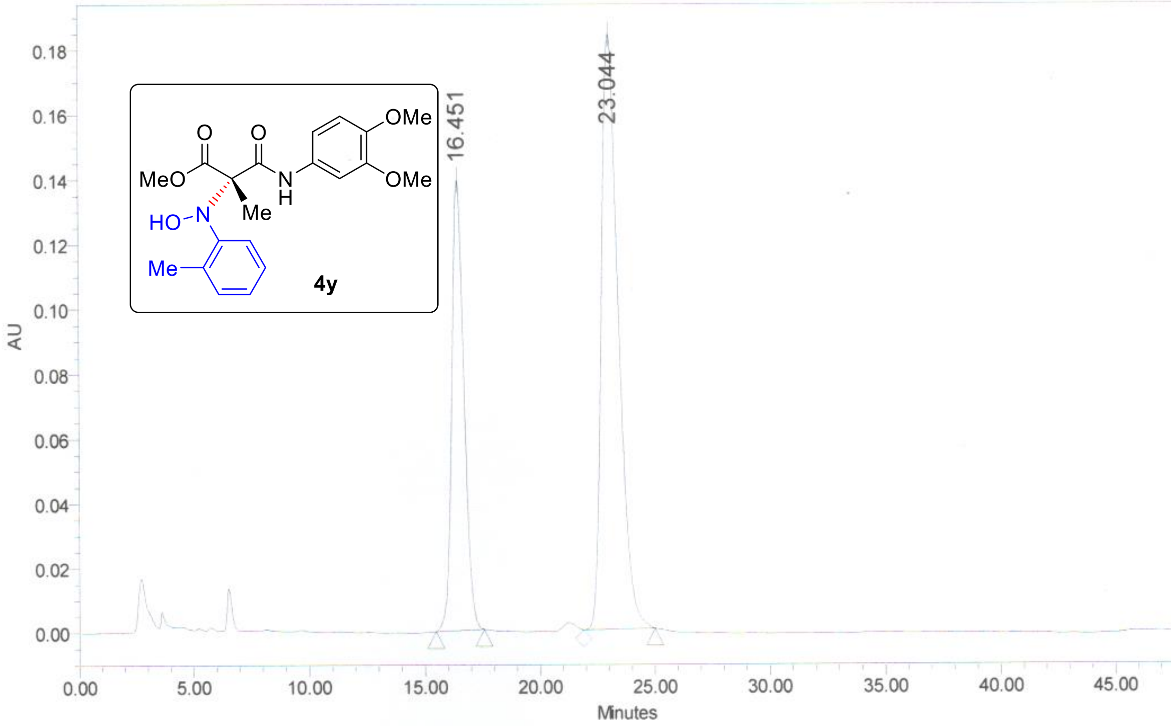

Peak Results

	Name	RT	Area	Height	% Area
1		16.451	5113176	139320	34.99
2		23.044	9499541	184048	65.01

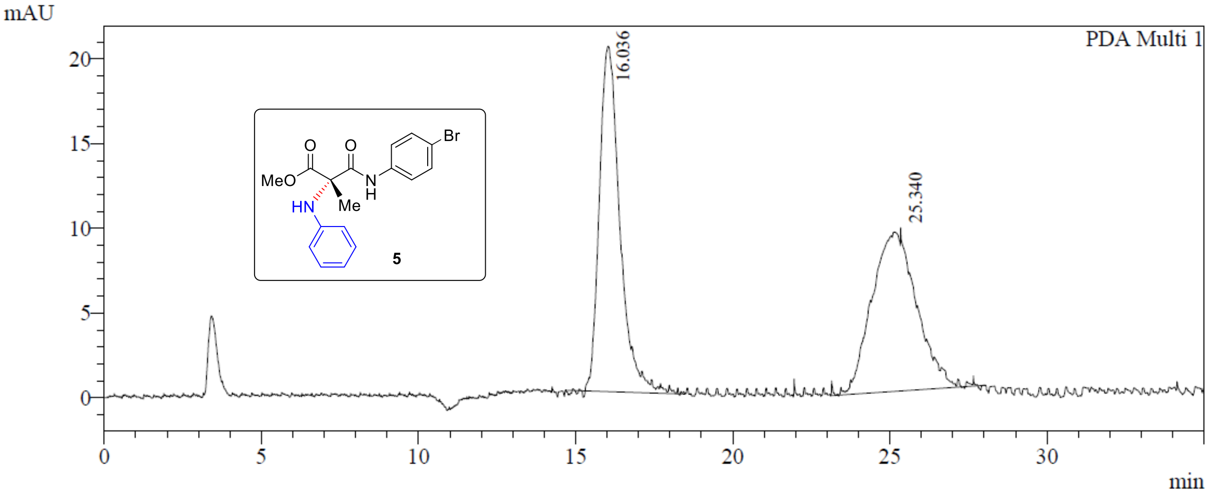

1 PDA Multi 1 / 251nm 4nm

PeakTable

Ch1 251nm 4nm

Peak#	Ret. Time	Area	Height	Area %
1	16.036	926140	20401	49.620
2	25.340	940332	9649	50.380
Total		1866472	30050	100.000

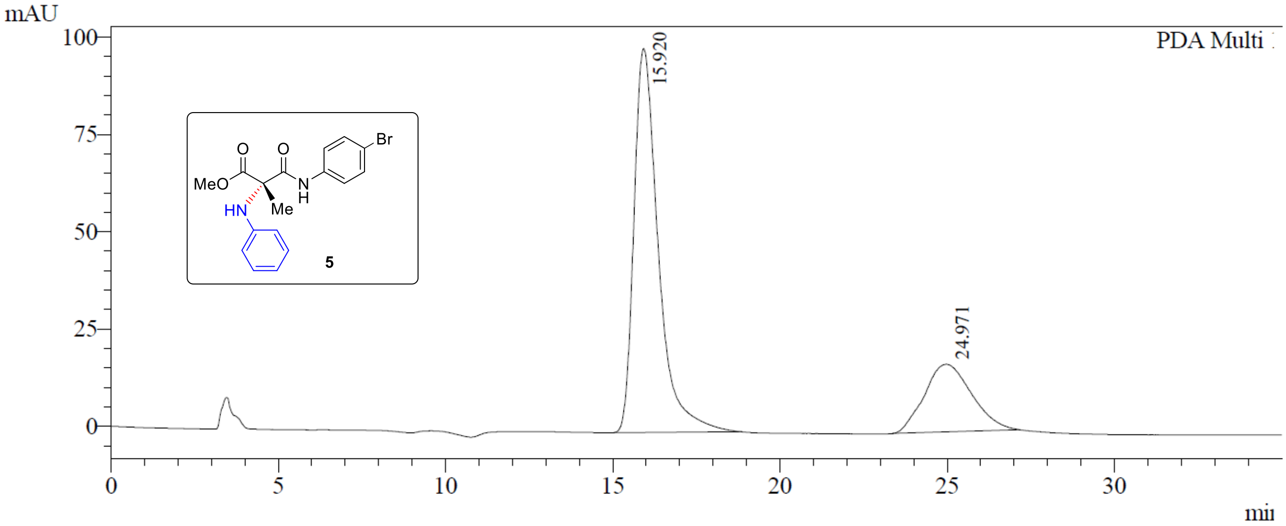

1 PDA Multi 1 / 250nm 4nm

PeakTable

Ch1 250nm 4nm

Peak#	Ret. Time	Area	Height	Area %
1	15.920	4998736	98606	74.547
2	24.971	1706783	17375	25.453
Total		6705519	115981	100.000
